# Intense interseasonal influenza outbreaks, Australia, 2018/19

**DOI:** 10.2807/1560-7917.ES.2019.24.33.1900421

**Published:** 2019-08-15

**Authors:** Ian G Barr, Yi Mo Deng, Miguel L Grau, Alvin X Han, Robin Gilmour, Melissa Irwin, Peter Markey, Kevin Freeman, Geoff Higgins, Mark Turra, Naomi Komadina, Heidi Peck, Robert Booy, Sebastian Maurer-Stroh, Vijaykrishna Dhanasekaran, Sheena Sullivan

**Affiliations:** 1 WHO Collaborating Centre for Reference and Research, Melbourne, Australia; 2 Department of Microbiology and Immunology, University of Melbourne, Melbourne, Australia; 3 Department of Microbiology, Biomedicine Discovery Institute Monash University, Clayton, Australia; 4 Bioinformatics Institute, Agency for Science, Technology and Research (A*STAR), Singapore; 5 National University of Singapore (NUS) Graduate School for Integrative Sciences and Engineering, Singapore; 6 Communicable Diseases Branch, Health Protection New South Wales, St. Leonards, Australia; 7 Rapid Surveillance, Centre for Epidemiology, New South Wales Ministry of Health, St. Leonards, Australia; 8 Centre for Disease Control, Northern Territory Department of Health, Darwin, Northern Territory, Australia; 9 Serology/Molecular Biology Territory Pathology, Royal Darwin Hospital, Northern Territory Government Health, Darwin, Australia; 10 Microbiology and Infectious Disease Directorate, SA Pathology, Adelaide, Australia; 11 National Centre for Immunisation Research and Surveillance (NCIRS), Westmead, Australia; 12 Department of Paediatrics and Adolescent Health, Faculty of Health and Medicine, University of Sydney, Sydney, Australia; 13 Department of Biological Sciences, National University of Singapore, Singapore

**Keywords:** seasonality, human, influenza, Australia

## Abstract

**Background:**

Interseasonal influenza outbreaks are not unusual in countries with temperate climates and well-defined influenza seasons. Usually, these are small and diminish before the main influenza season begins. However, the 2018/19 summer-autumn interseasonal influenza period in Australia saw unprecedented large and widespread influenza outbreaks.

**Aim:**

Our objective was to determine the extent of the intense 2018/19 interseasonal influenza outbreaks in Australia epidemiologically and examine the genetic, antigenic and structural properties of the viruses responsible for these outbreaks.

**Methods:**

This observational study combined the epidemiological and virological surveillance data obtained from the Australian Government Department of Health, the New South Wales Ministry of Health, sentinel outpatient surveillance, public health laboratories and data generated by the World Health Organization Collaborating Centre for Reference and Research on Influenza in Melbourne and the Singapore Agency for Science, Technology and Research.

**Results:**

There was a record number of laboratory-confirmed influenza cases during the interseasonal period November 2018 to May 2019 (n= 85,286; 5 times the previous 3-year average) and also more institutional outbreaks, hospitalisations and deaths, than what is normally seen.

**Conclusions:**

The unusually large interseasonal influenza outbreaks in 2018/19 followed a mild 2018 influenza season and resulted in a very early start to the 2019 influenza season across Australia. The reasons for this unusual event have yet to be fully elucidated but are likely to be a complex mix of climatic, virological and host immunity-related factors. These outbreaks reinforce the need for year-round surveillance of influenza, even in temperate climates with strong seasonality patterns.

## Introduction

**Figure 1:**
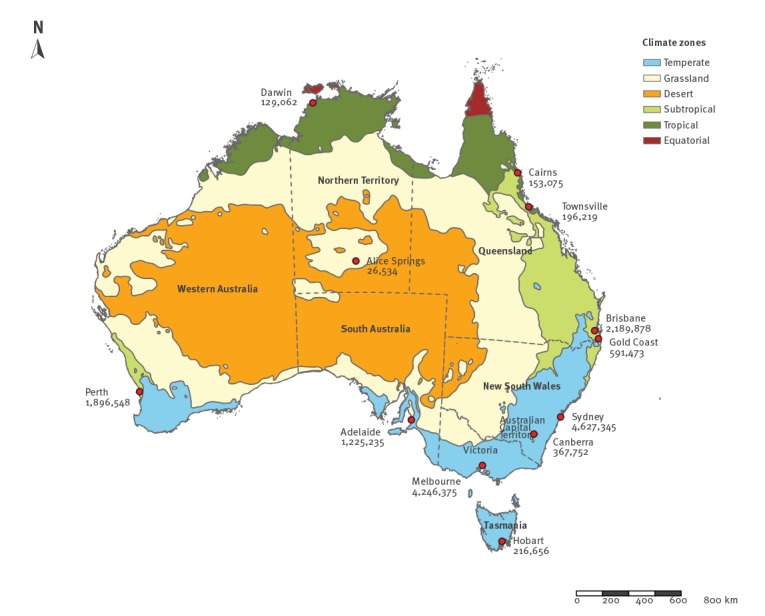
Climatic map of Australia, 2018

In 2018, the Australian influenza season was late and progressed with such minimal activity that it barely registered as a season by several surveillance indicators [1]. This was in stark contrast to the 2017 season, when Australia’s highest levels of influenza activity were recorded [2]. However, several surveillance indicators suggested that the influenza activity seen in 2018, while low, never really stopped, as it was expected to, at the end of the southern hemisphere spring (November). Instead, Australia experienced an upsurge in influenza cases with a large wet-season outbreak in the tropical north (see Figure 1), while southern Australia saw record numbers of laboratory-confirmed influenza notifications, increased hospitalisations and dozens of influenza-related deaths in late summer and early autumn, resulting in an early start to the 2019 influenza season throughout the country. Here we summarise the available epidemiological surveillance indicators along with a virological analysis of the influenza viruses collected during these 2018/19 interseasonal influenza outbreaks.

The plot shows the different climatic regions of Australia. The climatic regions of Australia are drawn according to the Köppen classification (Australian Bureau of Meteorology – obtained with permission – see: http://www.bom.gov.au/jsp/ncc/climate_averages/climate-classifications/index.jsp) with the populations of the main Australian cities in each state as of June 2018 (from the Australian Bureau of Statistics) added [32].

## Methods

Both epidemiological and virological surveillance data were used in this analysis and were derived from a number of established surveillance systems.

### Epidemiological data

The monthly count of laboratory-confirmed cases of influenza for each jurisdiction was obtained from the National Notifiable Diseases Surveillance System website [3] for the period January 2014 to May 2019. Laboratory data based on real-time PCR detection of influenza A and B as a proportion of total respiratory samples tested were provided by SA Pathology and the New South Wales (NSW) Ministry of Health. The weekly percentage of influenza-like illness (ILI) presentations at emergency departments (based on ICD-9, ICD-10 and SNOMED-CT codes) was provided by the Public Health Rapid, Emergency, Disease and Syndromic Surveillance (PHREDSS) system for the state of NSW. The weekly proportion of patients seen with ILI (fever/history of fever, cough and fatigue) at general practices was obtained from the Australian Sentinel Practices Research Network. Both raw data and the 5-week moving average for each of these data sources were plotted.

Other data cited in this paper (e.g. deaths, aged care outbreaks) were obtained from Australian influenza surveillance reports [4] and NSW influenza surveillance reports [5].

### Antigenic analysis of influenza viruses

Viruses received at the World Health Organization (WHO) Collaborating Centre for Reference and Research on Influenza (CCRRI) from Australian laboratories were inoculated into Madin-Darby Canine Kidney (MDCK) cells to obtain virus isolates. Haemagglutination inhibition (HI) assays were performed as previously described [6]. Focus reduction assays were used to determine influenza A(H3N2) antigenic characteristics using the same ferret antisera used in the HI assays basically as described previously [7] but with 1.2% Avicell RC591 (IMCD Mulgrave, Australia) replacing the carboxymethyl cellulose. Isolates were identified as antigenically similar to the reference viruses if the test samples had a titre that was no more than fourfold different from the homologous reference strain. Results were reported against reference antisera for influenza A/Michigan/45/2015 (2018/19 H1N1pdm09 vaccine virus), A/Singapore/INFIMH16–0019/2016 (2018 and 2018/19 H3N2 vaccine virus) and A/Switzerland/8060/2017 (2019 H3N2 vaccine virus)

### Phylogenetic analysis of influenza viruses

The haemagglutinin (HA) and neuraminidase (NA) gene sequences of all globally sampled influenza H1N1pdm09 and H3N2 viruses collected during the period from January 2018 to July 2019 were obtained from the Global Initiative on Sharing All Influenza (GISAID) [8] (these sequences and their origins are listed in Supplementary Table S1) and compared to the Australian seasonal influenza sequences. Sequence alignments were prepared using MAFFT v7 [9]. Following manual corrections, phylogenies were inferred using the maximum likelihood (ML) method in RaxML v8 [10] using the general time reversible nucleotide substitution model with gamma rate heterogeneity (GTR + Γ). We inferred the amino acid changes that occur along the branches of ML phylogenies using the ancestral sequence reconstruction method in Treetime v0.5 [11].

### Structural analysis of influenza viruses

FoldX (http://foldxsuite.crg.eu/) was used to estimate changes to structural stability of each amino acid substitution. The HA structure (protein data bank (PDB): 3UBQ (A(H1N1)pdm09 [12]) and 4O5N (A(H3N2) [13]) was first repaired by the software to remove any potential steric clashes before estimating the difference in free energy changes between the mutant and wild-type protein (i.e. ΔΔG = ΔG_mutant-ΔG_(wild type)) using default parameters (298K, ionic strength = 0.05M, pH = 7.0). Estimation of ΔΔG values were repeated five times for each substitution and the average resulting value (ΔΔG_mean) was taken. As the empirical force field model used by FoldX has a reported standard deviation of 0.46 kcal/mol between computed and experimental values, the tested substitution was assumed to have a destabilising effect if ΔΔG_mean > 0.46 kcal/mol and a stabilising effect if ΔΔG_mean < −0.46 kcal/mol. Amino acid substitutions relative to the closest vaccine precursor were identified using FluSurver (https://flusurver.bii.a-star.edu.sg/). The haemagglutinin structures were visualised with YASARA (v18.2.7; https://www.yasara.org).

### Ethical statement

This study used de-identified surveillance data available either publicly or on request, and their use did not require review by an ethics committee.

## Results

### Epidemiology of the influenza outbreaks

**Figure 2:**
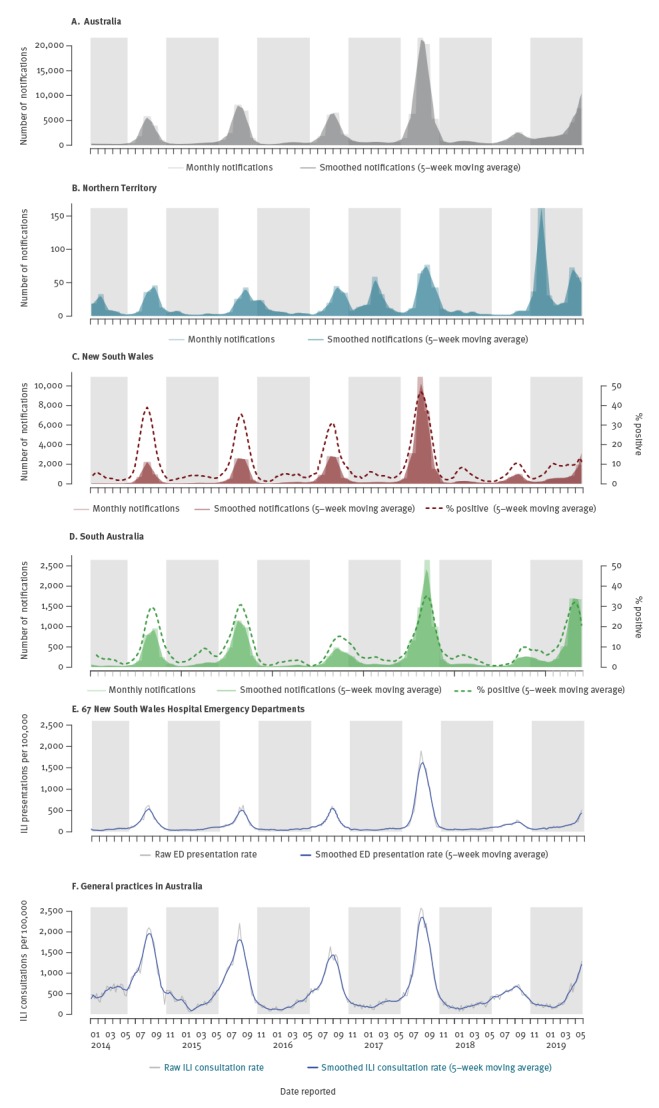
Selected influenza surveillance data, Australia, 2014–2019

ED: emergency department; ILI: influenza-like illness.

Laboratory-confirmed notifications of influenza for the whole of Australia from the National Notifications Disease Surveillance System (NNDSS [3]) showed above-average interseasonal (November to May) activity in 2018/19 (Figure 2A). Australia’s Northern Territory (NT) often experiences two influenza epidemics, the main one during the tropical dry season (June to August) that coincides with the southern temperate winter, and a smaller epidemic during the tropical wet season (November to April) which coincides with the southern temperate summer-autumn seasons. Unusually in 2018, there was minimal mid-year activity and an early wet-season, along with a large influenza epidemic (especially in and around Darwin - the capital city of the NT) that unusually peaked in December (Figure 2B). Following this severe outbreak, early and elevated influenza activity was also noted in most jurisdictions in the southern states of Australia, especially in South Australia during the first quarter of 2019 (Figure 2C, 2F).

Influenza laboratory-confirmed influenza notifications. Dotted line in panels C and D: percentage of influenza-positive samples. Light grey bars indicate the interseasonal period 1 November–31 May for each year. All plots show raw data plus 5-week moving averages. Note that the y-axes of the different panels are not set at the same scale.

The large number of notifications of laboratory-confirmed influenza for Australia that was seen in early 2019 continued to rise each month, with total notifications in March 2019 already comparable to peak notifications for 2018 (11,114 in March 2019 compared with 11,509 in September 2018). By the end of May, notifications were roughly 12-fold higher than the average number of notifications made in May during the previous 3 years [3], and the cumulative number of notifications made for the period 1 January to 31 May 2019 had surpassed the total notifications seen in 2018 (73,351 vs 58,868 respectively). Weekly rates of influenza positive tests between January and May 2019 ranged from 8.6% to 12.2% in NSW and from 4.8% to 35.4% in South Australia (denominator data was not available for the NT), (Figures 2B–D).

Most other jurisdictions (Queensland, Victoria, Tasmania) experienced increased influenza activity during the first 3–4 months of 2019 with only Western Australia and Australian Capital Territory (ACT) having lower levels [4]. All states showed an early start to the 2019 season and reached a peak weeks earlier than normal (ACT started in week 19 and peaked in week 28; NSW weeks 20 and 28; NT weeks 11 and 17; Queensland weeks 22 and 27; South Australia weeks 9 and 20; Tasmania week 8 with twin peaks in weeks 16 and 27; Victoria weeks 18 and 27 and Western Australia weeks 18 and 27) [14]. 

Hospital and General Practice ILI surveillance also indicated early influenza activity with emergency department presentations in NSW exceeding seasonal threshold in late April (Figures 2E, 2F). Also concerning in 2019 has been the number of deaths among confirmed influenza cases during the interseasonal period, which stood at 147 deaths for the period 1 January 2019 to 31 May, compared with only 23 deaths in 2018 and 19 deaths in 2017 over the same time period [4]. The number of influenza outbreaks in residential aged care facilities in Australia’s largest state NSW was also well above average, with 50 facilities reporting outbreaks between 1 January and 31 May 2019 compared with 11 outbreaks in 2018 and 14 outbreaks in 2017 over the same time period [15]. Limited Australian hospitalisation data available at the time of writing for the period 1 April to 2 June 2019 (weeks 14–22), showed substantially higher hospital admissions, with 589 admissions (adults and children), compared with 48 admissions in weeks 14–22, 2018 and 99 admissions in weeks 14–21, 2017 [4]. The proportion of patients admitted directly to intensive care units (ca 5% of cases) was similar to previous years [4].

Notably, notifications have been elevated in children in 2019. In the state of NSW, 33% of influenza notifications by 31 May were in children aged 0–16 years, compared with 22% in previous years. In NSW emergency departments, in the week ending 2 June 2019 for example, 29.8% of ILI presentations were in children aged 0–16 years, whereas in 2018 and 2017 children comprised around 19% of emergency presentations. Presentations of school-aged children to emergency departments also increased sharply 2 weeks after children returned to school after the Easter holidays (10–14 April 2019).

### Virology of the influenza outbreak viruses 

**Figure 3:**
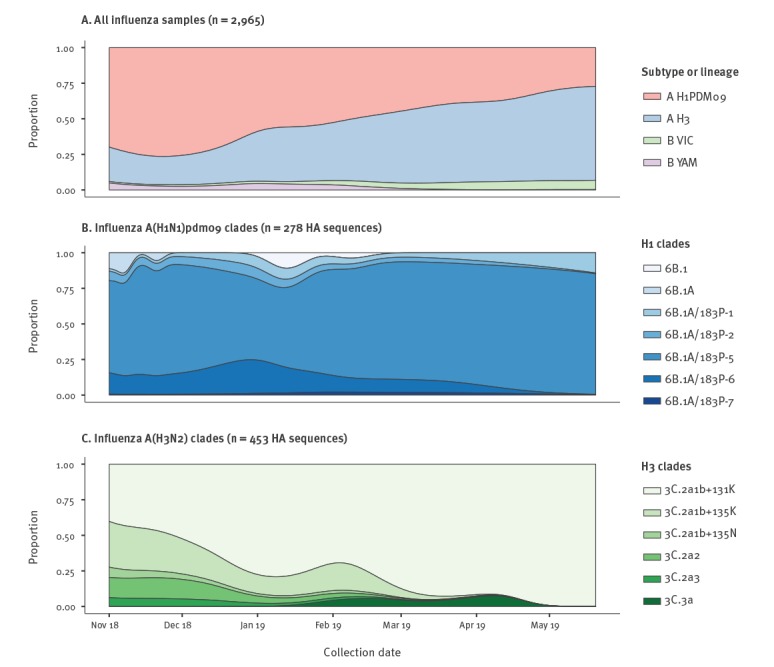
Virological influenza data for the interseasonal period, Australia, November 2018–May 2019 (n = 2,965)

Both influenza A subtypes circulated in Australia before November 2018, with influenza A(H1N1)pdm09 predominating throughout 2018 and into early 2019, while influenza A(H3N2) viruses increased rapidly in 2019 (Figure 3A). Influenza B also circulated at low levels, with both B/Victoria-lineage and B/Yamagata-lineage viruses (Figure 3A).

Panel A: Density plot showing the proportion of samples received at the World Health Organization Collaborating Centre for Reference and Research on Influenza (WHO CCRRI), Melbourne, by type, subtype and lineage. The chart shows the changing predominance of influenza A(H3N2) over A(H1N1)pdm09 and influenza B/Victoria over B/Yamagata circulation as time progresses from November 2018 to May 2019.

Panels B and C: Density plots showing the relative proportion of various genetic clades of viruses sequenced during this period by the WHO CCRRI as determined by their haemagglutinin gene phylogenetic associations.

### Phylogenetic and structural analysis of the influenza outbreak viruses

**Figure 4:**
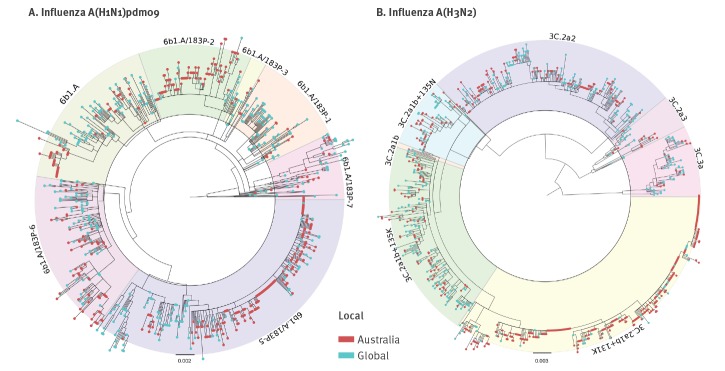
Evolutionary relationships of the haemagglutinin genes (maximum likelihood) of influenza A(H1N1)pdm09 (n = 835) and A(H3N2) viruses (n = 954), Australian haemagglutinin sequences shown from 2018/19 (n = 422 H1; n = 544 H3)

**Figure 5:**
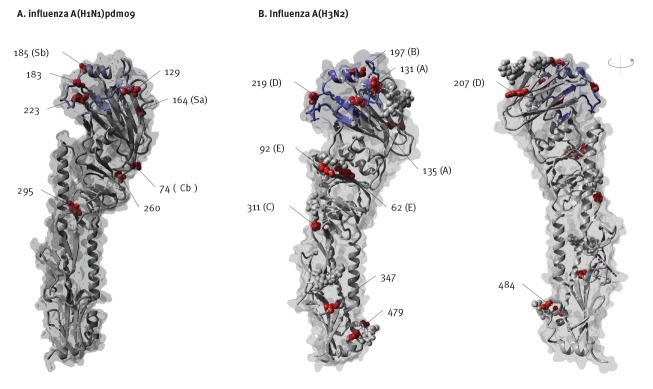
Structural images of influenza A(H1N1)pdm09 and A(H3N2) haemagglutinin molecules showing changes in the most commonly circulating clades in Australia, 2018/19

Phylogenetically there were dominant HA clades of viruses for both influenza A(H1N1)pdm09 and A(H3N2) viruses during the period 1 November 2018 to 31 May 2019 in Australia. For influenza A(H1N1)pdm09, the predominant clade was 6B1A 183P-5 (Nextstrain nomenclature [16]), which accounted for 201/278 (72.3%) of A(H1N1)pdm09 viruses sequenced by the WHO CCRRI during this period. A large majority of these viruses formed a subclade with further signature amino acid substitutions in the HA (N129D, T185I). This HA subclade was first detected in Darwin (NT) on 30 October 2018, spreading across the country between January and May 2019 (Figure 3B and Figure 4A). These N129D and T185I substitutions are structurally located in antigenic regions surrounding the receptor-binding pocket but not close enough to affect receptor binding (Figure 5A). Importantly, they are located at opposite sides of the pocket in distinct epitopes and hence do not have a cumulative effect on altering antigenicity. It should be noted that T185I is near the recently acquired S183P mutation that is one of the differing sites between older A/Michigan/45/2015 (clade 6B1) and new A/Brisbane/02/2018 (clade 6B1A 183P-1) vaccine strains and combination of the two could increase the antigenic distance to older strains. Using FoldX structural stability calculations [17], T185I and S183P have a significant stabilising effect while N129D has a predicted weakly destabilising effect (Supplementary Table S2).

Tip branches representing individual viruses collected since September 2018 in Australia are shown in red and those from overseas locations are shown in green. Clade designations are indicated on the circumference and separated by the coloured sections. For a full list of sequences used to generate these trees see the Global Initiative on Sharing All Influenza Data (GISAID) listing in Supplementary Table 1.

Receptor binding regions are shown in blue.

Panel A: Positions with amino acid changes in the haemagglutinin (HA) of a 6B1A 183-P5 A(H1N1)pdm09 clade virus relative to A/Michigan/45/2015 (H1N1pdm09; PDB: 4LXV) are shown in red and labelled according to A(H1N1)pdm09 position numbering without signal peptide; letters in brackets indicate if this change was in a known antigenic site.

Panel B: Positions with amino acid changes in the HA of recent 3C2a1b + 131K A(H3N2) clade viruses relative to A/SingaporeINFIMH-16–0019/2016 (H3N2; PDB: 4WEA) are shown in red and labelled according to A(H3N2) position numbering without signal peptide; letters in brackets indicate if this change was in a known antigenic site. We also indicate site 135 that controls removal of a glycosylation site in distinct subclade 3C2a1b + 135K. Supplementary Figure S5C shows the amino acid differences in recent HA of the recent 3C2a1b + 131K A(H3N2) clade viruses relative to the 2019 Australian and southern hemisphere vaccine A/Switzerland/8060/2017 (H3N2).

When we compared these influenza A(H1N1)pdm09 virus HA sequences phylogenetically to global influenza sequences collected during 2018/19 (see GISAID Supplementary Table S1), the predominant 6B1A 183P-5 clade viruses formed four independent clusters showing that they were introduced on at least four occasions into Australia during 2018 and 2019 (Figure 4A). One of the clusters contained a large number of samples collected around Darwin, NT, during November and December 2018 and included a smaller number of non-NT samples collected subsequently from across Australia, suggesting that the amplification of this lineage occurred in NT, followed by subsequent spread to other parts of the country. 

An influenza A(H3N2) HA clade which was in the minority in Australia in 2018, grew rapidly in proportion in early 2019 and represented 373 of 453 (82.3%) of influenza A(H3N2) viruses sequenced by the WHO CCRRI between 1 November 2018 and 31 May 2019 (Figure 3C). This clade has been designated as 3C.2a1b based on the HA gene phylogeny (Nextstrain nomenclature [16]) with a distinctive T131K change (i.e. 3C.2a1b-131K) in the HA along with other amino acid changes (K135T, S219F, V347M, V529I). In January 2019, further substitutions were prevalent at Q197R and E484G and most of these later viruses had K207R (Figure 3b). Beside the dominant subclade of 3C.2a1b-131K viruses in the current Australian season, it should be noted that there is a separate subclade of 3C.2a1b viruses with a distinctive T135K substitution (i.e. 3C.2a1b-135K) co-circulating globally (see GISAID Supplementary Table S1).

Both T131K and T135K mutations are structurally located at the fringe of the influenza A(H3N2) receptor-binding pocket (Figure 5B) which can potentially weakly influence binding properties through altered interactions with the galactose units of the human host receptor. Mutations in this ring around the binding pocket are also important for antibody recognition and can explain differences in antigenicity between clades with multiple substitutions in the same epitope region. Important in this context is that T135K removes a known N-glycosylation site at position 133 (antigenic site A) which, in principle, exposes the antigenic site to antibody access in subclade of 3C.2a1b-135K viruses. However, position 133 continue to retain N-linked glycosylation for the 3C.2a1b-131K viruses that are of interest here. Based on a recent glycan study, position 133 was detected to be glycosylated with high-mannose glycans along with other complex hybrid glycan forms [13]. Along with other glycosylated sites in the head region such as Asn122 which remains conserved in subclade 3C.2a1b-131K and is also decorated with branched, complex glycans [13], it is highly likely that there remains a high degree of antibody shielding within antigenic site A of these viruses. FoldX calculations [17] suggest T131K, together with other mutations in the clade, could have stabilising effects on the structure (Supplementary Table S2). 

Similar to the influenza A(H1N1)pdm09 6B1A 183P-5 clade, the source population of the dominant influenza A(H3N2) 3C.2a1b + 131K could not be discerned as viruses from multiple continents (including Asia and Europe) clustered with the Australian viruses. Both the Australian influenza A(H1N1)pdm09 and A(H3N2) HA sequence phylogeny plots showed large groups of monophyletic sequences, indicating the local spreading of viruses. However, in contrast to influenza A(H1N1)pdm09 where a large number of samples were first detected in Darwin, there was extensive nationwide detection of the 3C2a1b + 131K clade viruses within Australia during their amplification period in November and December 2018. Interestingly there was a large cluster of these viruses in South Australia (mainly in and around Adelaide, the capital) during March 2019, suggesting some regionally specific epidemic circulation. The influenza A(H3N2) 3C2a1b + 131K clade viruses had also circulated widely in Japan and China during 2018/19 and to a lesser extent in Europe and less so in the United States (US) where the 3C3a clade viruses predominated [16].

### Antigenic analysis of viruses

Influenza A(H1N1)pdm09 virus isolates generated from viruses that circulated between 1 November 2018 and 31 May 2019 appeared to be antigenically similar to the 2019 Australian and southern hemisphere egg-grown vaccine virus, A/Michigan/45/2015, as only 7.2% exhibited low reactivity (≥ 8-fold titre reductions compared with the homologous titre) with post-infection ferret reference antisera raised against egg-grown virus when tested by HI assay. In contrast, most of the influenza A(H3N2) virus isolates from this period (mostly clade 3C2a1b + 131K viruses) exhibited low reactivity (≥ 8-fold titre reductions) with post-infection ferret antisera raised to egg-grown viruses A/Singapore/INFIMH16–0019/2016 (the 2018 southern hemisphere influenza vaccine and the 2018/19 northern hemisphere vaccine; a clade 3C2a1 virus) and to the 2019 Australian and southern hemisphere A(H3N2) vaccine virus A/Switzerland/8060/2017 (a clade 3C2a2 virus) by virus neutralisation (74.2% and 89.5% respectively). 

## Discussion

The 2018/19 interseasonal period (November to May) in Australia has been exceptional. Not only have notifications of laboratory-confirmed cases been high, but testing positivity rates, institutional outbreaks, deaths, and emergency department and general practice presentations have all also been high. Together, these indicators signalled an early start to the 2019 Australian influenza season. It remains to be seen if this will result in extended influenza circulation or if the season will end prematurely compared with a normal season. By early July, it appeared that peak notifications had been reached in most states of Australia – which is at least a month before the normal seasonal influenza peak, with cases thereafter declining [3,14]. However, 2019 is already the second biggest influenza season in the last 20 years in Australia, with some 197,768 laboratory-confirmed cases up to 7 August, second only to the 2017 season when there were 251,159 notifications [3]. The total notifications reported between January and February 2019 was roughly 2.6-fold higher than the average number reported for this period during the previous 3 years. This is not unprecedented; for example in 2011, there was a roughly sixfold increase in notifications detected between January and March compared with the average of the 3 previous years. However, that high activity declined thereafter and by a number of surveillance measures, 2011 was overall considered a mild year. At that time, this increase in notifications was attributed to increased use of PCR testing, and without denominator data it can be difficult to interpret laboratory-confirmed influenza notifications [18,19]. However, the elevated activity in 2019 cannot be fully attributed to changes in testing practices because the percentage of tests positive for influenza has also been higher than expected in at least two jurisdictions, NSW and South Australia. 

While Australia has a widely varied climate, the majority (over 70%) of the Australian population live in the temperate climatic region. Consequently, influenza seasonality largely follows a winter-spring pattern (June to October) that usually peaks around early August. It is not uncommon to see late summer outbreaks in tropical and subtropical regions of Australia (e.g. in Darwin) but their contribution to the total burden is usually minor owing to the small populations in these regions. Although reporting of interseasonal influenza has been increasing in recent years in Australia, possibly due to wider availability of testing [19], a comparison of the summer-to-winter ratio of laboratory-confirmed influenza cases for 2005/16 suggested no significant increase in interseasonal influenza in that period. However, during the 2018/19 interseasonal period, influenza activity was truly exceptional and cannot be accounted for by merely increased availability of testing. Other southern hemisphere countries (Argentina, Chile, New Zealand and South Africa) showed few or no laboratory-confirmed influenza cases from 1 January to 31 March 2019 as reported via WHO FluNet (https://www.who.int/influenza/gisrs_laboratory/flunet/charts/en/) and their seasonal influenza peaks also fell within their expected timespans. Normally, Australian influenza seasons begin between weeks 20.6 and 30.9 [20] and have peaked over the past 5 years (2014–19) from weeks 33–37 [4]. However, in 2019, most states started their influenza season before this earlier date with some starting very early (South Australia week 9 and Tasmania week 8) and all reached their peaks much earlier than in recent years [4].

The role of vaccination in driving influenza seasonality remains poorly understood. Vaccine is usually delivered from March to May in Australia each year and is not expected to continue to provide strong, residual protection after 9 months or more [21]. In response to the 2017 season, several changes were made to publicly funded vaccination programmes in Australia in 2018. This included funded vaccine for children younger than 5 years in most jurisdictions, which saw vaccine uptake increase substantially from around 5% to 30% (reaching a figure comparable with the uptake in adults aged 18–65 years). Yet, the high interseasonal activity has disproportionately affected children. This fits with modelling data by Mossong et al. that showed that in a fully susceptible population, 5–19-year-olds would be expected to suffer the highest incidence during the initial epidemic phase of an emerging infection transmitted through social contacts [22]. For adults 65 years and older, among whom vaccine uptake normally exceeds 80% in Australia, high dose and adjuvanted vaccines replaced standard dose vaccines in the national immunisation programme during 2018. It is difficult to quantify the impact changes to vaccine policy and uptake had on transmission in 2018 and subsequent population-level susceptibility by the end of the year. The early and severe start to the season in 2019 has prompted distribution of a record 12.5 million doses (enough for ca 50% of the entire eligible population) compared with 9.6 million distributed doses in 2018 and 8.3 million doses in 2017 [23]. It is still unclear by how much protection the 2018 vaccine will have provided and similarly if the 2019 vaccine will have mitigated transmission at all, given that it was being rolled out as the season was beginning.

Interseasonal or summer outbreaks of influenza have been reported previously in several places such as Taiwan [24], Hong Kong [25] and Okinawa [26] and are often due to A(H3N2) viruses. The reasons for the unusual and widespread interseasonal 2018/19 outbreaks in Australia are likely to be complex and due to multiple factors. The weather during this period was generally hotter than average, reached record high temperatures in many regions across Australia and was also drier than average, although in the tropical north-east (Queensland), there was wide spread flooding in early 2019 [27]. The mild 2018 influenza season may also have contributed as it meant a larger susceptible population. In 2019 (up to 31 May), notification rates were highest in those aged 80 years or older (517/100,000), mainly caused by influenza A(H3N2) viruses, and second highest in children (< 5 years; 492/100,000), mainly caused by influenza A(H1N1)pdm09 viruses [4].

The co-circulation of both influenza A subtypes especially in these groups and emerging clades that did not circulate widely during the normal influenza winter season in 2018 in Australia, may also be responsible for the higher rates of infection. The influenza A(H1N1)pdm09 6B1A 183P-5 clade viruses made up nearly 75% of the viruses sequenced during this period (1 November 2018 to 31 May 2019), first occurring in Darwin in northern Australia, then spreading to other states; similarly, the A(H3N2) viruses were dominated by the 3C2a1b + 131K clade with over 80% of the viruses sequenced during this period belonging to this clade. Viruses that were part of this 6B1A 183P-5 clade were also the major clade seen in the European 2018/19 influenza season where influenza A(H1N1)pdm09 viruses predominated, although other clades (6B1A 183P-7, 6B1A 183P-5 P6, 6B1A) also co-circulated [16,28]. In Canada, the 2018/19 season was also dominated by influenza A(H1N1)pdm09 (more than 90% of influenza viruses tested) where there was also genetic heterogeneity in the HA genes of circulating viruses with six clades detected and the 6B1A 183P-5 clade in minority (12% of viruses sequenced) [29]. Importantly this heterogeneity did not appear to affect the vaccine effectiveness (VE) for influenza A(H1N1)pdm09 which was estimated to be 72% (95%CI; 60,81) [29]. 

This implies that the A(H1N1)pmd09 and A(H3N2) viruses circulating in Australia either had some viral fitness advantage or were able to evade existing immunity better than other previously circulating virus clades and may have sufficient antigenic changes to also reduce vaccine effectiveness. The 2018 vaccine may also have been suboptimal against these circulating strains given that the period of peak coverage had probably been exceeded when these outbreaks occurred, some 7–11 months following vaccination [21]. The influenza A(H3N2) component of the southern hemisphere vaccine for 2019 (a 3C.2a2re* virus) may also be suboptimal in terms of its VE against recently circulating viruses, given the poor inhibition in HI assays of circulating Australian A(H3N2) viruses by ferret sera raised to the 2019 H3N2 vaccine virus, that has also been reported in another recent publication [30]. In contrast, the good inhibition of circulating Australian A(H1N1)pdm09 viruses in HI assays by ferret sera raised to the 2019 H1N1pdm09 vaccine virus suggests that these viruses will be well covered by the 2019 influenza vaccine (assuming the same viruses continue to circulate through the winter and spring).

These outbreaks in Australia reinforce the need for year-round surveillance of influenza even in regions with temperate climates with strong seasonality patterns such as Europe and North and South America. Early identification of major outbreaks can forewarn primary practitioners, aged care institutions and at-risk groups to consider bringing forward vaccination programmes (if feasible) or being alert to respiratory outbreaks and ensuring stocks of antiviral medications are on hand. It would also help alert hospitals and clinics of possible increased attendances and the possible cause of people presenting with ILI. With high levels of summer tourists coming to Australia each year from the northern hemisphere winter (such as the US, Japan and China) or from tropical regions near Australia where influenza circulates all year (such as Singapore, Indonesia and Malaysia), there will inevitably be further introductions of influenza in the future, as we have noted previously [31]. A better understanding of the reasons why the summer-autumn influenza outbreaks in Australia in 2018/19 were so prevalent may help to mitigate their impact on the population in the future.

## *Authors’ correction

The influenza A(H3N2) component of the southern hemisphere vaccine for 2019 was corrected from ‘3C.2a1’ to ‘3C.2a2re’. This correction was made on 16 August 2019, upon request of the authors.

## Supplementary Data

193000 Supplementary Tables

## Supplementary Data

584000 Supplementary Figure
