# Authors’ correction for Euro Surveill. 2019;24(33)

**DOI:** 10.2807/1560-7917.ES.2019.24.34.1908221

**Published:** 2019-08-22

**Authors:** Eurosurveillance editorial team

**Affiliations:** 1 European Centre for Disease Prevention and Control (ECDC), Stockholm, Sweden

For the article ‘Intense interseasonal influenza outbreaks, Australia, 2018/19’ by Ian G Barr et al., published on 15 August 2019, the influenza A(H3N2) component of the southern hemisphere vaccine for 2019 was corrected from ‘3C.2a1’ to ‘3C.2a2re’. This correction was made on 16 August 2019, upon request of the authors.

