# Supplementary material for: Intense interseasonal influenza outbreaks, Australia, 2018/19
Source: Euro Surveill. 2019 Aug 15;24(33):1900421. doi: 10.2807/1560-7917.ES.2019.24.33.1900421 (PMC6702793; doi:10.2807/1560-7917.ES.2019.24.33.1900421)
Supplement: Supplementary Tables [file 19-00421_BARR_SupplementaryTables.pdf]

Supplementary Table 1

A(H1N1)pdm09 HA sequences used in Figure 4a obtained from GISAID https://www.gisaid.org/

This supplementary material is hosted by *Eurosurveillance* as supporting information alongside the article "Intense interseasonal influenza outbreaks in Australia in 2018-9" on behalf of the authors who remain responsible for the accuracy and appropriateness of the content. The same standards for ethics, copyright, attributions and permissions as for the article apply. *Eurosurveillance* is not responsible for the maintenance of any links or email addresses provided therein

strain	country	date	subtype	australia	GISAID
A/Sudan/1/2018|Africa|02/27/2019	Africa	02/27/2019	6b1.A/183P5	Global	EPI_ISL_361988
A/Mauritius/I-14/2019|Africa|01/07/2019	Africa	01/07/2019	6b1.A/183P5	Global	EPI_ISL_355409
A/Mauritius/I-1092/2018|Africa|12/16/2018	Africa	12/16/2018	6b1.A/183P5	Global	EPI_ISL_355407
A/Mauritius/I-02/2019|Africa|01/03/2019	Africa	01/03/2019	6b1.A/183P5	Global	EPI_ISL_355402
A/Oujda/11116/2019|Africa|02/04/2019	Africa	02/04/2019	6b1.A/183P5	Global	EPI_ISL_355426
A/BeniMellal/1405/2019|Africa|02/01/2019	Africa	02/01/2019	6b1.A/183P5	Global	EPI_ISL_355284
A/Sierra_Leone/129/2018|Africa|09/19/2018	Africa	09/19/2018	6b1.A/183P5	Global	EPI_ISL_336637
A/Sierra_Leone/105/2018|Africa|09/12/2018	Africa	09/12/2018	6b1.A/183P5	Global	EPI_ISL_336635
A/Nigeria/3626/2018|Africa|11/07/2018	Africa	11/07/2018	6b1.A/183P5	Global	EPI_ISL_353346
A/South_Africa/VW0379/2018|Africa|05/02/2018	Africa	05/02/2018	6b1.A/183P5		EPI_ISL_320602
A/Mauritius/I-545/2018|Africa|07/08/2018	Africa	07/08/2018	6b1.A/183P5		EPI_ISL_332834
A/Dakar/30/2018|Africa|09/24/2018	Africa	09/24/2018	6b1.A/183P5	Global	EPI_ISL_355307
A/Dakar/33/2018|Africa|10/31/2018	Africa	10/31/2018	6b1.A/183P5	Global	EPI_ISL_355309
A/Burkina_Faso/835/2018|Africa|02/07/2018	Africa	02/07/2018	6b1.A/183P5		EPI_ISL_330377
A/Ghana/3693/2018|Africa|11/01/2018	Africa	11/01/2018	6b1.A/183P5	Global	EPI_ISL_347357
A/Ghana/3699/2018|Africa|11/01/2018	Africa	11/01/2018	6b1.A/183P5	Global	EPI_ISL_347358
A/Ghana/2345/2018|Africa|07/09/2018	Africa	07/09/2018	6b1.A/183P5		EPI_ISL_332824
A/Ghana/3865/2018|Africa|10/02/2018	Africa	10/02/2018	6b1.A/183P5	Global	EPI_ISL_338049
A/Ghana/594/2018|Africa|08/23/2018	Africa	08/23/2018	6b1.A/183P5		EPI_ISL_332829
A/Ghana/2597/2018|Africa|07/30/2018	Africa	07/30/2018	6b1.A/183P5		EPI_ISL_332826

A/Agadir/1414/2019|Africa|02/08/2019	Africa	02/08/2019	6b1.A/183P6	Global	EPI_ISL_355258
A/Fes/1380/2019|Africa|02/05/2019	Africa	02/05/2019	6b1.A/183P6	Global	EPI_ISL_355322
A/Fes/416/2019|Africa|01/15/2019	Africa	01/15/2019	6b1.A/183P6	Global	EPI_ISL_355325
A/Fes/506/2019|Africa|01/17/2019	Africa	01/17/2019	6b1.A/183P6	Global	EPI_ISL_355326
A/South_Africa/7423/2018|Africa|05/28/2018	Africa	05/28/2018	6b1.A/183P6		EPI_ISL_330843
A/Antsirabe/2784/2018|Africa|06/18/2018	Africa	06/18/2018	6b1.A/183P6		EPI_ISL_321825
A/Antsirabe/2475/2018|Africa|05/30/2018	Africa	05/30/2018	6b1.A/183P6		EPI_ISL_321823
A/Uganda/6825/2018|Africa|04/19/2018	Africa	04/19/2018	6b1.A/183P6		EPI_ISL_319839
A/Antsirabe/1679/2018|Africa|04/24/2018	Africa	04/24/2018	6b1.A/183P6		EPI_ISL_321822
A/Antsirabe/4203/2018|Africa|09/17/2018	Africa	09/17/2018	6b1.A/183P6	Global	EPI_ISL_355270
A/Kenya/133/2018|Africa|04/09/2018	Africa	04/09/2018	6b1.A/183P6		EPI_ISL_331951
A/Kenya/104/2018|Africa|03/01/2018	Africa	03/01/2018	6b1.A/183P6		EPI_ISL_319798
A/Kenya/127/2018|Africa|03/09/2018	Africa	03/09/2018	6b1.A/183P6		EPI_ISL_331953
A/Egypt/458/2018|Africa|01/15/2018	Africa	01/15/2018	6b1.A/183P6		EPI_ISL_315705
A/SouthAfrica/VW0412/2018|Africa|06/06/2018	Africa	06/06/2018	6b1.A/183P6		EPI_ISL_329770
A/South_Africa/R08871/2018|Africa|06/18/2018	Africa	06/18/2018	6b1.A/183P6		EPI_ISL_330852
A/SouthAfrica/R10112/2018|Africa|07/04/2018	Africa	07/04/2018	6b1.A/183P6		EPI_ISL_321836
A/South_Africa/R07337/2018|Africa|05/25/2018	Africa	05/25/2018	6b1.A/183P6		EPI_ISL_321485
A/Algeria/248/2018|Africa|01/03/2018	Africa	01/03/2018	6b1.A/183P6		EPI_ISL_298997
A/Antsirabe/4579/2018|Africa|10/01/2018	Africa	10/01/2018	6b1.A/183P6	Global	EPI_ISL_355272
A/Antsirabe/4399/2018|Africa|09/27/2018	Africa	09/27/2018	6b1.A/183P6	Global	EPI_ISL_355271
A/Dakar/26/2018|Africa|01/08/2018	Africa	01/08/2018	6b1.A/183P6		EPI_ISL_355306
A/Mauritius/I-1077/2018|Africa|12/12/2018	Africa	12/12/2018	6b1.A/183P1	Global	EPI_ISL_355406
A/SouthAfrica/R05547/2018|Africa|04/23/2018	Africa	04/23/2018	6b1.A/183P1		EPI_ISL_329762
A/South_Africa/6597/2018|Africa|05/14/2018	Africa	05/14/2018	6b1.A/183P1		EPI_ISL_330836
A/Kenya/139/2018|Africa|07/23/2018	Africa	07/23/2018	6b1.A		EPI_ISL_335881
A/Kenya/117/2018|Africa|03/20/2018	Africa	03/20/2018	6b1.A		EPI_ISL_331958
A/Meknes/217/2018|Africa|12/13/2018	Africa	12/13/2018	6b1.A	Global	EPI_ISL_355415
A/Meknes/387/2019|Africa|01/14/2019	Africa	01/14/2019	6b1.A	Global	EPI_ISL_355416
A/Meknes/1070/2018|Africa|02/01/2018	Africa	02/01/2018	6b1.A		EPI_ISL_308459
A/Tunis/418/2018|Africa|01/06/2018	Africa	01/06/2018	6b1.A		EPI_ISL_309979
A/Burkina_Faso/906/2018|Africa|02/26/2018	Africa	02/26/2018	6b1.A		EPI_ISL_330390
A/Dakar/01/2018|Africa|02/07/2018	Africa	02/07/2018	6b1.A		EPI_ISL_321826
A/Algeria/386/2018|Africa|01/14/2018	Africa	01/14/2018	6b1.A		EPI_ISL_308510

A/Nigeria/3399/2018|Africa|02/27/2018	Africa	02/27/2018	6b1.A		EPI_ISL_330825
A/Nigeria/3632/2018|Africa|11/08/2018	Africa	11/08/2018	6b1.A	Global	EPI_ISL_355508
A/Nigeria/3691/2018|Africa|11/27/2018	Africa	11/27/2018	6b1.A	Global	EPI_ISL_351848
A/Nigeria/3577/2018|Africa|10/05/2018	Africa	10/05/2018	6b1.A	Global	EPI_ISL_351874
A/Nigeria/3586/2018|Africa|10/10/2018	Africa	10/10/2018	6b1.A	Global	EPI_ISL_355491
A/South_Africa/09664/2018|Africa|06/26/2018	Africa	06/26/2018	6b1.A		EPI_ISL_330857
A/South_Africa/VW0449/2018|Africa|06/21/2018	Africa	06/21/2018	6b1.A		EPI_ISL_330302
A/South_Africa/R05200/2018|Africa|04/13/2018	Africa	04/13/2018	6b1.A		EPI_ISL_321482
A/Mozambique/7118/2018|Africa|03/20/2018	Africa	03/20/2018	6b1.A		EPI_ISL_320257
A/Mozambique/6218/2018|Africa|03/16/2018	Africa	03/16/2018	6b1.A		EPI_ISL_320260
A/Taiwan/81946/2019|Asia|03/07/2019	Asia	03/07/2019	6b1.A/183P5	Global	EPI_ISL_352136
A/YOKOHAMA/141/2019|Asia|03/08/2019	Asia	03/08/2019	6b1.A/183P5	Global	EPI_ISL_350494
A/Kuwait/447/2019|Asia|01/17/2019	Asia	01/17/2019	6b1.A/183P5	Global	EPI_ISL_349873
A/SAPPORO/63/2018|Asia|12/17/2018	Asia	12/17/2018	6b1.A/183P5	Global	EPI_ISL_340039
A/YOKOHAMA/98/2019|Asia|02/08/2019	Asia	02/08/2019	6b1.A/183P5	Global	EPI_ISL_356744
A/KANAGAWA/IC1873/2019|Asia|01/24/2019	Asia	01/24/2019	6b1.A/183P5	Global	EPI_ISL_356418
A/Taiwan/81950/2019|Asia|03/08/2019	Asia	03/08/2019	6b1.A/183P5	Global	EPI_ISL_352137
A/KANAGAWA/AC1837/2019|Asia|01/18/2019	Asia	01/18/2019	6b1.A/183P5	Global	EPI_ISL_356415
A/Bursa/1874/2018|Asia|12/12/2018	Asia	12/12/2018	6b1.A/183P5	Global	EPI_ISL_355295
A/Xinjiang-Kelamayi/1142/2019|Asia|02/13/2019	Asia	02/13/2019	6b1.A/183P5	Global	EPI_ISL_344253
A/KANAGAWA/ZC1807/2019|Asia|01/09/2019	Asia	01/09/2019	6b1.A/183P5	Global	EPI_ISL_342369
A/MIE/16/2019|Asia|03/15/2019	Asia	03/15/2019	6b1.A/183P5	Global	EPI_ISL_356756
A/Nonthaburi/96/2018|Asia|12/15/2018	Asia	12/15/2018	6b1.A/183P5	Global	EPI_ISL_339877
A/Oman/6335/2018|Asia|11/19/2018	Asia	11/19/2018	6b1.A/183P5	Global	EPI_ISL_332864
A/Iran/138156/2018|Asia|09/25/2018	Asia	09/25/2018	6b1.A/183P5	Global	EPI_ISL_338059
A/Georgia/365/2018|Asia|04/10/2018	Asia	04/10/2018	6b1.A/183P5		EPI_ISL_315964
A/Oman/2723/2019|Asia|03/20/2019	Asia	03/20/2019	6b1.A/183P6	Global	EPI_ISL_357921
A/Bangladesh/291/2018|Asia|08/09/2018	Asia	08/09/2018	6b1.A/183P6		EPI_ISL_333518
A/Shanghai-Jingan/SWL13362/2018|Asia|07/23/2018	Asia	07/23/2018	6b1.A/183P6		EPI_ISL_334941
A/Bangladesh/237/2018|Asia|09/18/2018	Asia	09/18/2018	6b1.A/183P6	Global	EPI_ISL_333528
A/Bangladesh/03242/2018|Asia|08/13/2018	Asia	08/13/2018	6b1.A/183P6		EPI_ISL_333529
A/Saudi_Arabia/458398/2018|Asia|08/07/2018	Asia	08/07/2018	6b1.A/183P6		EPI_ISL_355450
A/Bangladesh/976/2018|Asia|06/24/2018	Asia	06/24/2018	6b1.A/183P6		EPI_ISL_330415
A/Macau/601567/2018|Asia|02/28/2018	Asia	02/28/2018	6b1.A/183P6		EPI_ISL_312225

A/Surat_Thani/61/2018|Asia|03/06/2018	Asia	03/06/2018	6b1.A/183P6		EPI_ISL_322767
A/Maldives/483/2018|Asia|05/19/2018	Asia	05/19/2018	6b1.A/183P6		EPI_ISL_330840
A/HK/2869/2018|Asia|11/25/2018	Asia	11/25/2018	6b1.A/183P6	Global	EPI_ISL_337476
A/Astana/ZVL329/2018|Asia|02/20/2018	Asia	02/20/2018	6b1.A/183P6		EPI_ISL_303788
A/Singapore/GP0429/2018|Asia|02/13/2018	Asia	02/13/2018	6b1.A/183P6		EPI_ISL_321718
A/Neimenggu-Donghe/SWL1514/2018|Asia|11/12/2018	Asia	11/12/2018	6b1.A/183P6	Global	EPI_ISL_336908
A/Fujian-Gulou/1884/2018|Asia|11/05/2018	Asia	11/05/2018	6b1.A/183P6	Global	EPI_ISL_353415
A/Macau/600196/2019|Asia|01/10/2019	Asia	01/10/2019	6b1.A/183P6	Global	EPI_ISL_351750
A/shenzhen/2019-14147/2018|Asia|07/30/2018	Asia	07/30/2018	6b1.A/183P1		EPI_ISL_344301
A/Laos/F3052/2018|Asia|10/23/2018	Asia	10/23/2018	6b1.A/183P1	Global	EPI_ISL_360182
A/Laos/F3029/2018|Asia|10/22/2018	Asia	10/22/2018	6b1.A/183P1	Global	EPI_ISL_360175
A/Laos/2173/2018|Asia|09/05/2018	Asia	09/05/2018	6b1.A/183P1	Global	EPI_ISL_354345
A/Singapore/TT0488/2018|Asia|04/26/2018	Asia	04/26/2018	6b1.A/183P1		EPI_ISL_321743
A/Hong_Kong/1114/2018|Asia|06/13/2018	Asia	06/13/2018	6b1.A/183P1		EPI_ISL_320829
A/HK/1026/2018|Asia|05/31/2018	Asia	05/31/2018	6b1.A/183P1		EPI_ISL_319850
A/Dundgobi/1558/2018|Asia|05/16/2018	Asia	05/16/2018	6b1.A/183P1		EPI_ISL_320183
A/Xinjiang-Midong/SWL1558/2018|Asia|10/31/2018	Asia	10/31/2018	6b1.A/183P1	Global	EPI_ISL_334575
A/Hong_Kong/645/2018|Asia|04/08/2018	Asia	04/08/2018	6b1.A/183P1		EPI_ISL_314199
A/Guangxi-Yuzhou/SWL1612/2018|Asia|06/16/2018	Asia	06/16/2018	6b1.A/183P1		EPI_ISL_334972
A/Fujian-Siming/SWL1582/2018|Asia|07/23/2018	Asia	07/23/2018	6b1.A/183P1		EPI_ISL_334939
A/Fujian-Siming/SWL1332/2018|Asia|04/23/2018	Asia	04/23/2018	6b1.A/183P1		EPI_ISL_334929
A/Singapore/KK0353/2018|Asia|04/23/2018	Asia	04/23/2018	6b1.A/183P2		EPI_ISL_321746
A/Pakistan/1341/2019|Asia|02/12/2019	Asia	02/12/2019	6b1.A/183P2	Global	EPI_ISL_351825
A/Yunnan-Linxiang/1769/2018|Asia|07/20/2018	Asia	07/20/2018	6b1.A/183P2		EPI_ISL_334926
A/HK/2374/2018|Asia|10/17/2018	Asia	10/17/2018	6b1.A/183P2	Global	EPI_ISL_332122
A/Guangdong-Xiangzhou/SWL8296/2018|Asia|06/05/2018	Asia	06/05/2018	6b1.A/183P2		EPI_ISL_334964
A/HK/1822/2018|Asia|09/03/2018	Asia	09/03/2018	6b1.A/183P2	Global	EPI_ISL_331323
A/SAITAMA-C/13/2019|Asia|02/01/2019	Asia	02/01/2019	6b1.A/183P2	Global	EPI_ISL_346651
A/HIROSHIMA/71/2018|Asia|10/10/2018	Asia	10/10/2018	6b1.A/183P2	Global	EPI_ISL_336220
A/SAITAMA/158/2018|Asia|12/15/2018	Asia	12/15/2018	6b1.A/183P7	Global	EPI_ISL_344508
A/Kazakhstan/013/2019|Asia|02/05/2019	Asia	02/05/2019	6b1.A/183P7	Global	EPI_ISL_355519
A/Kyrgyzstan/127/2018|Asia|12/12/2018	Asia	12/12/2018	6b1.A/183P7	Global	EPI_ISL_347660
A/Viet_Nam/SARI02CC18014/2018|Asia|02/03/2018	Asia	02/03/2018	6b1.A/183P7		EPI_ISL_327878
A/Nakhonphanom/1308/2018|Asia|07/16/2018	Asia	07/16/2018	6b1.A/183P7		EPI_ISL_322741

A/Laos/F2185/2018|Asia|09/03/2018	Asia	09/03/2018	6b1.A/183P7	Global	EPI_ISL_352373
A/Guangdong-Zhongshan/SWL1551/2018|Asia|06/06/2018	Asia	06/06/2018	6b1.A/183P7		EPI_ISL_334979
A/NAGANO/2658/2018|Asia|11/27/2018	Asia	11/27/2018	6b1.A	Global	EPI_ISL_336225
A/Lebanon/12/2018|Asia|03/17/2018	Asia	03/17/2018	6b1.A		EPI_ISL_330827
A/Jordan/245/2018|Asia|01/09/2018	Asia	01/09/2018	6b1.A		EPI_ISL_314240
A/Jordan/225/2018|Asia|01/08/2018	Asia	01/08/2018	6b1.A		EPI_ISL_314239
A/Philippines/0008/2018|Asia|01/04/2018	Asia	01/04/2018	6b1.A		EPI_ISL_319845
A/Bahrain/362/2018|Asia|05/13/2018	Asia	05/13/2018	6b1.A		EPI_ISL_315807
A/Jordan/20271/2018|Asia|01/23/2018	Asia	01/23/2018	6b1.A		EPI_ISL_315708
A/Saudi_Arabia/463062/2018|Asia|08/26/2018	Asia	08/26/2018	6b1.A		EPI_ISL_355454
A/Bangkok/96/2018|Asia|05/15/2018	Asia	05/15/2018	6b1.A		EPI_ISL_322769
A/Seoul/1288/2018|Asia|03/07/2018	Asia	03/07/2018	6b1.A		EPI_ISL_321832
A/Pakistan/1531/2018|Asia|02/19/2018	Asia	02/19/2018	6b1.A		EPI_ISL_331427
A/shenzhen/2019-14228/2018|Asia|03/06/2018	Asia	03/06/2018	6b1.A		EPI_ISL_344377
A/shenzhen/2019-14202/2018|Asia|08/14/2018	Asia	08/14/2018	6b1.A		EPI_ISL_344353
A/Zhejiang-Yiwu/SWL128/2018|Asia|01/10/2018	Asia	01/10/2018	6b1.A		EPI_ISL_309776
A/Bangkok/77/2018|Asia|03/27/2018	Asia	03/27/2018	6b1.A		EPI_ISL_329890
A/Sydney/732/2019|Australia|02/21/2019	Australia	02/21/2019	6b1.A/183P5	Australia	EPI_ISL_354700
A/South_Australia/127/2019|Australia|03/03/2019	Australia	03/03/2019	6b1.A/183P5	Australia	EPI_ISL_354705
A/Darwin/92/2018|Australia|11/26/2018	Australia	11/26/2018	6b1.A/183P5	Australia	EPI_ISL_334013
A/Townsville/42/2018|Australia|11/25/2018	Australia	11/25/2018	6b1.A/183P5	Australia	EPI_ISL_341430
A/Darwin/123/2018|Australia|11/25/2018	Australia	11/25/2018	6b1.A/183P5	Australia	EPI_ISL_341405
A/Darwin/117/2018|Australia|11/25/2018	Australia	11/25/2018	6b1.A/183P5	Australia	EPI_ISL_334017
A/Darwin/80/2018|Australia|11/21/2018	Australia	11/21/2018	6b1.A/183P5	Australia	EPI_ISL_334008
A/Darwin/68/2018|Australia|11/21/2018	Australia	11/21/2018	6b1.A/183P5	Australia	EPI_ISL_334073
A/Darwin/66/2018|Australia|11/20/2018	Australia	11/20/2018	6b1.A/183P5	Australia	EPI_ISL_334007
A/Darwin/55/2018|Australia|11/19/2018	Australia	11/19/2018	6b1.A/183P5	Australia	EPI_ISL_334071
A/Darwin/21/2018|Australia|11/18/2018	Australia	11/18/2018	6b1.A/183P5	Australia	EPI_ISL_333986
A/Darwin/22/2018|Australia|11/18/2018	Australia	11/18/2018	6b1.A/183P5	Australia	EPI_ISL_333987
A/Darwin/26/2018|Australia|11/18/2018	Australia	11/18/2018	6b1.A/183P5	Australia	EPI_ISL_333990
A/Darwin/20/2018|Australia|11/17/2018	Australia	11/17/2018	6b1.A/183P5	Australia	EPI_ISL_333985
A/Darwin/37/2018|Australia|11/16/2018	Australia	11/16/2018	6b1.A/183P5	Australia	EPI_ISL_333995
A/Darwin/14/2018|Australia|11/06/2018	Australia	11/06/2018	6b1.A/183P5	Australia	EPI_ISL_332064
A/Darwin/12/2018|Australia|10/23/2018	Australia	10/23/2018	6b1.A/183P5	Australia	EPI_ISL_332063

A/Darwin/13/2018|Australia|10/22/2018	Australia	10/22/2018	6b1.A/183P5	Australia	EPI_ISL_332061
A/Victoria/910/2019|Australia|03/01/2019	Australia	03/01/2019	6b1.A/183P5	Australia	EPI_ISL_354482
A/Newcastle/27/2019|Australia|03/09/2019	Australia	03/09/2019	6b1.A/183P5	Australia	EPI_ISL_354687
A/South_Australia/29/2019|Australia|02/06/2019	Australia	02/06/2019	6b1.A/183P5	Australia	EPI_ISL_351748
A/Canberra/7/2019|Australia|01/07/2019	Australia	01/07/2019	6b1.A/183P5	Australia	EPI_ISL_354779
A/Brisbane/2/2019|Australia|01/04/2019	Australia	01/04/2019	6b1.A/183P5	Australia	EPI_ISL_339342
A/Victoria/21/2019|Australia|04/15/2019	Australia	04/15/2019	6b1.A/183P5	Australia	EPI_ISL_355188
A/Newcastle/29/2019|Australia|03/11/2019	Australia	03/11/2019	6b1.A/183P5	Australia	EPI_ISL_354688
A/Sydney/30/2019|Australia|03/10/2019	Australia	03/10/2019	6b1.A/183P5	Australia	EPI_ISL_354473
A/Townsville/12/2019|Australia|02/19/2019	Australia	02/19/2019	6b1.A/183P5	Australia	EPI_ISL_354469
A/Sydney/730/2019|Australia|02/17/2019	Australia	02/17/2019	6b1.A/183P5	Australia	EPI_ISL_354699
A/Canberra/346/2018|Australia|12/03/2018	Australia	12/03/2018	6b1.A/183P5	Australia	EPI_ISL_334078
A/South_Australia/95/2019|Australia|03/01/2019	Australia	03/01/2019	6b1.A/183P5	Australia	EPI_ISL_352132
A/South_Australia/1005/2019|Australia|03/07/2019	Australia	03/07/2019	6b1.A/183P5	Australia	EPI_ISL_354703
A/Sydney/55/2019|Australia|03/11/2019	Australia	03/11/2019	6b1.A/183P5	Australia	EPI_ISL_355185
A/Darwin/1830/2018|Australia|12/02/2018	Australia	12/02/2018	6b1.A/183P5	Australia	EPI_ISL_339180
A/Darwin/141/2018|Australia|12/01/2018	Australia	12/01/2018	6b1.A/183P5	Australia	EPI_ISL_339179
A/Sydney/711/2019|Australia|02/02/2019	Australia	02/02/2019	6b1.A/183P5	Australia	EPI_ISL_354471
A/Darwin/60/2018|Australia|11/21/2018	Australia	11/21/2018	6b1.A/183P5	Australia	EPI_ISL_334003
A/Darwin/88/2019|Australia|02/26/2019	Australia	02/26/2019	6b1.A/183P5	Australia	EPI_ISL_354497
A/Darwin/48/2018|Australia|11/19/2018	Australia	11/19/2018	6b1.A/183P5	Australia	EPI_ISL_334067
A/Sydney/51/2019|Australia|03/08/2019	Australia	03/08/2019	6b1.A/183P5	Australia	EPI_ISL_355184
A/Victoria/918/2019|Australia|03/20/2019	Australia	03/20/2019	6b1.A/183P5	Australia	EPI_ISL_354697
A/Sydney/752/2019|Australia|01/25/2019	Australia	01/25/2019	6b1.A/183P5	Australia	EPI_ISL_354694
A/South_Australia/133/2019|Australia|03/05/2019	Australia	03/05/2019	6b1.A/183P5	Australia	EPI_ISL_354707
A/Sydney/29/2019|Australia|03/03/2019	Australia	03/03/2019	6b1.A/183P5	Australia	EPI_ISL_354478
A/Newcastle/34/2019|Australia|03/13/2019	Australia	03/13/2019	6b1.A/183P5	Australia	EPI_ISL_354689
A/Newcastle/41/2019|Australia|03/18/2019	Australia	03/18/2019	6b1.A/183P5	Australia	EPI_ISL_354691
A/Sydney/32/2019|Australia|03/18/2019	Australia	03/18/2019	6b1.A/183P5	Australia	EPI_ISL_354447
A/Sydney/50/2019|Australia|03/07/2019	Australia	03/07/2019	6b1.A/183P5	Australia	EPI_ISL_355183
A/Sydney/34/2019|Australia|03/19/2019	Australia	03/19/2019	6b1.A/183P5	Australia	EPI_ISL_354708
A/Brisbane/11/2019|Australia|02/28/2019	Australia	02/28/2019	6b1.A/183P5	Australia	EPI_ISL_354698
A/Perth/3/2019|Australia|01/05/2019	Australia	01/05/2019	6b1.A/183P5	Australia	EPI_ISL_339281
A/South_Australia/100/2019|Australia|02/27/2019	Australia	02/27/2019	6b1.A/183P5	Australia	EPI_ISL_354704

A/Newcastle/78/2018|Australia|12/29/2018	Australia	12/29/2018	6b1.A/183P5	Australia	EPI_ISL_339050
A/Darwin/53/2018|Australia|11/19/2018	Australia	11/19/2018	6b1.A/183P5	Australia	EPI_ISL_334069
A/Darwin/52/2018|Australia|11/19/2018	Australia	11/19/2018	6b1.A/183P5	Australia	EPI_ISL_334001
A/Darwin/18/2018|Australia|11/18/2018	Australia	11/18/2018	6b1.A/183P5	Australia	EPI_ISL_333983
A/Darwin/176/2018|Australia|11/30/2018	Australia	11/30/2018	6b1.A/183P5	Australia	EPI_ISL_336007
A/Victoria/2083/2019|Australia|02/16/2019	Australia	02/16/2019	6b1.A/183P5	Australia	EPI_ISL_354446
A/Darwin/201/2018|Australia|12/06/2018	Australia	12/06/2018	6b1.A/183P5	Australia	EPI_ISL_339188
A/Darwin/126/2018|Australia|11/25/2018	Australia	11/25/2018	6b1.A/183P5	Australia	EPI_ISL_333982
A/Darwin/188/2018|Australia|12/04/2018	Australia	12/04/2018	6b1.A/183P5	Australia	EPI_ISL_338247
A/Darwin/143/2018|Australia|11/30/2018	Australia	11/30/2018	6b1.A/183P5	Australia	EPI_ISL_338246
A/Darwin/171/2018|Australia|11/29/2018	Australia	11/29/2018	6b1.A/183P5	Australia	EPI_ISL_336006
A/Darwin/86/2018|Australia|11/22/2018	Australia	11/22/2018	6b1.A/183P5	Australia	EPI_ISL_338258
A/Darwin/90/2018|Australia|11/22/2018	Australia	11/22/2018	6b1.A/183P5	Australia	EPI_ISL_334012
A/Perth/10/2019|Australia|01/20/2019	Australia	01/20/2019	6b1.A/183P5	Australia	EPI_ISL_355189
A/Sydney/193/2018|Australia|12/08/2018	Australia	12/08/2018	6b1.A/183P5	Australia	EPI_ISL_339217
A/Darwin/93/2019|Australia|03/08/2019	Australia	03/08/2019	6b1.A/183P5	Australia	EPI_ISL_354481
A/Victoria/2075/2019|Australia|02/11/2019	Australia	02/11/2019	6b1.A/183P5	Australia	EPI_ISL_354504
A/Townsville/8/2019|Australia|02/10/2019	Australia	02/10/2019	6b1.A/183P5	Australia	EPI_ISL_354467
A/Victoria/2030/2019|Australia|01/17/2019	Australia	01/17/2019	6b1.A/183P5	Australia	EPI_ISL_354421
A/Newcastle/38/2019|Australia|03/18/2019	Australia	03/18/2019	6b1.A/183P5	Australia	EPI_ISL_354690
A/Brisbane/1001/2019|Australia|01/06/2019	Australia	01/06/2019	6b1.A/183P5	Australia	EPI_ISL_339341
A/South_Australia/120/2019|Australia|03/08/2019	Australia	03/08/2019	6b1.A/183P5	Australia	EPI_ISL_354710
A/Darwin/95/2019|Australia|03/14/2019	Australia	03/14/2019	6b1.A/183P5	Australia	EPI_ISL_354474
A/Sydney/191/2018|Australia|12/11/2018	Australia	12/11/2018	6b1.A/183P5	Australia	EPI_ISL_339216
A/Darwin/259/2018|Australia|12/11/2018	Australia	12/11/2018	6b1.A/183P5	Australia	EPI_ISL_334080
A/Darwin/189/2018|Australia|12/05/2018	Australia	12/05/2018	6b1.A/183P5	Australia	EPI_ISL_336009
A/Darwin/199/2018|Australia|12/04/2018	Australia	12/04/2018	6b1.A/183P5	Australia	EPI_ISL_339187
A/Darwin/185/2018|Australia|12/03/2018	Australia	12/03/2018	6b1.A/183P5	Australia	EPI_ISL_336008
A/Darwin/1930/2018|Australia|12/02/2018	Australia	12/02/2018	6b1.A/183P5	Australia	EPI_ISL_339181
A/Darwin/160/2018|Australia|11/28/2018	Australia	11/28/2018	6b1.A/183P5	Australia	EPI_ISL_336005
A/Darwin/124/2018|Australia|11/27/2018	Australia	11/27/2018	6b1.A/183P5	Australia	EPI_ISL_341406
A/Darwin/110/2018|Australia|11/26/2018	Australia	11/26/2018	6b1.A/183P5	Australia	EPI_ISL_334016
A/Darwin/119/2018|Australia|11/25/2018	Australia	11/25/2018	6b1.A/183P5	Australia	EPI_ISL_341403
A/Darwin/122/2018|Australia|11/25/2018	Australia	11/25/2018	6b1.A/183P5	Australia	EPI_ISL_341404

A/Darwin/98/2018|Australia|11/25/2018	Australia	11/25/2018	6b1.A/183P5	Australia	EPI_ISL_334015
A/Darwin/81/2018|Australia|11/24/2018	Australia	11/24/2018	6b1.A/183P5	Australia	EPI_ISL_334009
A/Darwin/87/2018|Australia|11/23/2018	Australia	11/23/2018	6b1.A/183P5	Australia	EPI_ISL_334011
A/Darwin/84/2018|Australia|11/22/2018	Australia	11/22/2018	6b1.A/183P5	Australia	EPI_ISL_334085
A/Darwin/75/2018|Australia|11/22/2018	Australia	11/22/2018	6b1.A/183P5	Australia	EPI_ISL_334065
A/Darwin/49/2018|Australia|11/21/2018	Australia	11/21/2018	6b1.A/183P5	Australia	EPI_ISL_334068
A/Darwin/65/2018|Australia|11/20/2018	Australia	11/20/2018	6b1.A/183P5	Australia	EPI_ISL_334006
A/Darwin/47/2018|Australia|11/20/2018	Australia	11/20/2018	6b1.A/183P5	Australia	EPI_ISL_334066
A/Darwin/19/2018|Australia|11/20/2018	Australia	11/20/2018	6b1.A/183P5	Australia	EPI_ISL_333984
A/Darwin/28/2018|Australia|11/19/2018	Australia	11/19/2018	6b1.A/183P5	Australia	EPI_ISL_333991
A/Darwin/54/2018|Australia|11/19/2018	Australia	11/19/2018	6b1.A/183P5	Australia	EPI_ISL_334070
A/Darwin/50/2018|Australia|11/19/2018	Australia	11/19/2018	6b1.A/183P5	Australia	EPI_ISL_333999
A/Darwin/32/2018|Australia|11/17/2018	Australia	11/17/2018	6b1.A/183P5	Australia	EPI_ISL_333992
A/Darwin/38/2018|Australia|11/15/2018	Australia	11/15/2018	6b1.A/183P5	Australia	EPI_ISL_333996
A/Darwin/25/2018|Australia|11/15/2018	Australia	11/15/2018	6b1.A/183P5	Australia	EPI_ISL_333989
A/Darwin/23/2018|Australia|11/14/2018	Australia	11/14/2018	6b1.A/183P5	Australia	EPI_ISL_333988
A/Darwin/42/2018|Australia|11/12/2018	Australia	11/12/2018	6b1.A/183P5	Australia	EPI_ISL_333997
A/Darwin/421/2018|Australia|12/20/2018	Australia	12/20/2018	6b1.A/183P5	Australia	EPI_ISL_351753
A/Newcastle/147/2018|Australia|12/17/2018	Australia	12/17/2018	6b1.A/183P5	Australia	EPI_ISL_339202
A/Darwin/269/2018|Australia|12/07/2018	Australia	12/07/2018	6b1.A/183P5	Australia	EPI_ISL_334079
A/Darwin/67/2018|Australia|11/21/2018	Australia	11/21/2018	6b1.A/183P5	Australia	EPI_ISL_334072
A/Darwin/104/2019|Australia|03/21/2019	Australia	03/21/2019	6b1.A/183P5	Australia	EPI_ISL_354702
A/Darwin/98/2019|Australia|03/18/2019	Australia	03/18/2019	6b1.A/183P5	Australia	EPI_ISL_354475
A/Darwin/89/2019|Australia|03/01/2019	Australia	03/01/2019	6b1.A/183P5	Australia	EPI_ISL_354498
A/Darwin/74/2019|Australia|02/11/2019	Australia	02/11/2019	6b1.A/183P5	Australia	EPI_ISL_342362
A/Darwin/57/2019|Australia|01/23/2019	Australia	01/23/2019	6b1.A/183P5	Australia	EPI_ISL_341426
A/Darwin/51/2018|Australia|11/19/2018	Australia	11/19/2018	6b1.A/183P5	Australia	EPI_ISL_334000
A/Darwin/36/2018|Australia|11/15/2018	Australia	11/15/2018	6b1.A/183P5	Australia	EPI_ISL_333994
A/Victoria/746/2018|Australia|11/14/2018	Australia	11/14/2018	6b1.A/183P5	Australia	EPI_ISL_338253
A/Darwin/88/2018|Australia|11/23/2018	Australia	11/23/2018	6b1.A/183P5	Australia	EPI_ISL_338259
A/Darwin/6/2018|Australia|11/03/2018	Australia	11/03/2018	6b1.A/183P5	Australia	EPI_ISL_349822
A/Darwin/5/2018|Australia|10/30/2018	Australia	10/30/2018	6b1.A/183P5	Australia	EPI_ISL_338263
A/Darwin/69/2018|Australia|11/22/2018	Australia	11/22/2018	6b1.A/183P5	Australia	EPI_ISL_334074
A/Darwin/118/2018|Australia|11/24/2018	Australia	11/24/2018	6b1.A/183P5	Australia	EPI_ISL_338245

A/Darwin/191/2018|Australia|12/03/2018	Australia	12/03/2018	6b1.A/183P5	Australia	EPI_ISL_338248
A/Darwin/103/2018|Australia|11/25/2018	Australia	11/25/2018	6b1.A/183P5	Australia	EPI_ISL_338262
A/Darwin/91/2018|Australia|11/23/2018	Australia	11/23/2018	6b1.A/183P5	Australia	EPI_ISL_338260
A/Darwin/193/2018|Australia|12/03/2018	Australia	12/03/2018	6b1.A/183P5	Australia	EPI_ISL_338275
A/Darwin/196/2018|Australia|12/02/2018	Australia	12/02/2018	6b1.A/183P5	Australia	EPI_ISL_338249
A/Darwin/125/2018|Australia|11/27/2018	Australia	11/27/2018	6b1.A/183P5	Australia	EPI_ISL_341407
A/Darwin/95/2018|Australia|11/25/2018	Australia	11/25/2018	6b1.A/183P5	Australia	EPI_ISL_338261
A/Darwin/85/2018|Australia|11/22/2018	Australia	11/22/2018	6b1.A/183P5	Australia	EPI_ISL_334010
A/Darwin/72/2018|Australia|11/22/2018	Australia	11/22/2018	6b1.A/183P5	Australia	EPI_ISL_334075
A/Darwin/74/2018|Australia|11/22/2018	Australia	11/22/2018	6b1.A/183P5	Australia	EPI_ISL_334076
A/Darwin/96/2018|Australia|11/25/2018	Australia	11/25/2018	6b1.A/183P5	Australia	EPI_ISL_362334
A/Darwin/61/2018|Australia|11/20/2018	Australia	11/20/2018	6b1.A/183P5	Australia	EPI_ISL_334004
A/Darwin/64/2018|Australia|11/19/2018	Australia	11/19/2018	6b1.A/183P5	Australia	EPI_ISL_334005
A/Darwin/35/2018|Australia|11/16/2018	Australia	11/16/2018	6b1.A/183P5	Australia	EPI_ISL_333993
A/Darwin/44/2018|Australia|11/17/2018	Australia	11/17/2018	6b1.A/183P5	Australia	EPI_ISL_333998
A/Darwin/79/2018|Australia|11/23/2018	Australia	11/23/2018	6b1.A/183P5	Australia	EPI_ISL_334077
A/Darwin/73/2019|Australia|02/11/2019	Australia	02/11/2019	6b1.A/183P5	Australia	EPI_ISL_351742
A/Victoria/2063/2019|Australia|02/02/2019	Australia	02/02/2019	6b1.A/183P5	Australia	EPI_ISL_354503
A/Darwin/79/2019|Australia|02/16/2019	Australia	02/16/2019	6b1.A/183P5	Australia	EPI_ISL_351741
A/Darwin/64/2019|Australia|01/31/2019	Australia	01/31/2019	6b1.A/183P5	Australia	EPI_ISL_354713
A/Darwin/43/2019|Australia|01/12/2019	Australia	01/12/2019	6b1.A/183P5	Australia	EPI_ISL_339262
A/Townsville/11/2019|Australia|02/18/2019	Australia	02/18/2019	6b1.A/183P5	Australia	EPI_ISL_354463
A/Sydney/1012/2019|Australia|03/04/2019	Australia	03/04/2019	6b1.A/183P5	Australia	EPI_ISL_352133
A/Sydney/33/2019|Australia|03/18/2019	Australia	03/18/2019	6b1.A/183P5	Australia	EPI_ISL_354479
A/Victoria/2066/2019|Australia|02/04/2019	Australia	02/04/2019	6b1.A/183P5	Australia	EPI_ISL_354445
A/Victoria/14/2019|Australia|03/25/2019	Australia	03/25/2019	6b1.A/183P5	Australia	EPI_ISL_354766
A/Townsville/10/2019|Australia|02/16/2019	Australia	02/16/2019	6b1.A/183P5	Australia	EPI_ISL_354462
A/Townsville/14/2019|Australia|02/26/2019	Australia	02/26/2019	6b1.A/183P5	Australia	EPI_ISL_354470
A/Newcastle/23/2019|Australia|03/11/2019	Australia	03/11/2019	6b1.A/183P5	Australia	EPI_ISL_354768
A/Newcastle/86/2018|Australia|12/27/2018	Australia	12/27/2018	6b1.A/183P5	Australia	EPI_ISL_336003
A/Victoria/2031/2019|Australia|01/17/2019	Australia	01/17/2019	6b1.A/183P5	Australia	EPI_ISL_354422
A/Victoria/10/2019|Australia|02/10/2019	Australia	02/10/2019	6b1.A/183P5	Australia	EPI_ISL_341433
A/Brisbane/1000/2019|Australia|01/03/2019	Australia	01/03/2019	6b1.A/183P5	Australia	EPI_ISL_345231
A/Sydney/49/2019|Australia|03/07/2019	Australia	03/07/2019	6b1.A/183P5	Australia	EPI_ISL_354696

A/Newcastle/26/2019|Australia|03/13/2019	Australia	03/13/2019	6b1.A/183P5	Australia	EPI_ISL_354695
A/South_Australia/213/2018|Australia|12/04/2018	Australia	12/04/2018	6b1.A/183P5	Australia	EPI_ISL_339222
A/Victoria/2061/2019|Australia|02/01/2019	Australia	02/01/2019	6b1.A/183P5	Australia	EPI_ISL_354476
A/Victoria/2064/2019|Australia|02/03/2019	Australia	02/03/2019	6b1.A/183P5	Australia	EPI_ISL_354465
A/Victoria/701/2019|Australia|03/26/2019	Australia	03/26/2019	6b1.A/183P5	Australia	EPI_ISL_348400
A/Tasmania/15/2018|Australia|11/17/2018	Australia	11/17/2018	6b1.A/183P5	Australia	EPI_ISL_334084
A/Sydney/722/2019|Australia|02/08/2019	Australia	02/08/2019	6b1.A/183P5	Australia	EPI_ISL_354464
A/Tasmania/10/2018|Australia|11/22/2018	Australia	11/22/2018	6b1.A/183P5	Australia	EPI_ISL_338251
A/Brisbane/8/2019|Australia|01/31/2019	Australia	01/31/2019	6b1.A/183P5	Australia	EPI_ISL_354780
A/Brisbane/9/2019|Australia|02/14/2019	Australia	02/14/2019	6b1.A/183P5	Australia	EPI_ISL_354461
A/Townsville/5/2019|Australia|01/28/2019	Australia	01/28/2019	6b1.A/183P5	Australia	EPI_ISL_354466
A/Newcastle/20/2019|Australia|03/08/2019	Australia	03/08/2019	6b1.A/183P5	Australia	EPI_ISL_354767
A/Tasmania/503/2018|Australia|11/04/2018	Australia	11/04/2018	6b1.A/183P5	Australia	EPI_ISL_332779
A/Sydney/27/2019|Australia|03/09/2019	Australia	03/09/2019	6b1.A/183P5	Australia	EPI_ISL_354477
A/Victoria/2089/2019|Australia|02/21/2019	Australia	02/21/2019	6b1.A/183P5	Australia	EPI_ISL_354506
A/South_Australia/37/2018|Australia|08/29/2018	Australia	08/29/2018	6b1.A/183P5		EPI_ISL_330108
A/South_Australia/49/2018|Australia|09/12/2018	Australia	09/12/2018	6b1.A/183P5	Australia	EPI_ISL_332492
A/Brisbane/1019/2018|Australia|10/17/2018	Australia	10/17/2018	6b1.A/183P5	Australia	EPI_ISL_332066
A/Brisbane/1020/2018|Australia|10/17/2018	Australia	10/17/2018	6b1.A/183P5	Australia	EPI_ISL_332776
A/Darwin/4/2018|Australia|10/28/2018	Australia	10/28/2018	6b1.A/183P5	Australia	EPI_ISL_336000
A/Perth/1035/2018|Australia|10/12/2018	Australia	10/12/2018	6b1.A/183P5	Australia	EPI_ISL_332775
A/Perth/148/2018|Australia|10/01/2018	Australia	10/01/2018	6b1.A/183P5	Australia	EPI_ISL_332757
A/Perth/1009/2018|Australia|07/14/2018	Australia	07/14/2018	6b1.A/183P5		EPI_ISL_322994
A/Perth/1017/2018|Australia|08/08/2018	Australia	08/08/2018	6b1.A/183P5		EPI_ISL_322779
A/Canberra/17/2018|Australia|07/03/2018	Australia	07/03/2018	6b1.A/183P5		EPI_ISL_323007
A/Canberra/12/2018|Australia|06/26/2018	Australia	06/26/2018	6b1.A/183P5		EPI_ISL_323006
A/Perth/1022/2018|Australia|09/03/2018	Australia	09/03/2018	6b1.A/183P5	Australia	EPI_ISL_330098
A/Port_Hedland/1000/2019|Australia|02/11/2019	Australia	02/11/2019	6b1.A/183P5	Australia	EPI_ISL_354496
A/South_Australia/172/2018|Australia|11/05/2018	Australia	11/05/2018	6b1.A/183P6	Australia	EPI_ISL_338252
A/South_Australia/166/2018|Australia|11/07/2018	Australia	11/07/2018	6b1.A/183P6	Australia	EPI_ISL_338277
A/South_Australia/152/2018|Australia|11/01/2018	Australia	11/01/2018	6b1.A/183P6	Australia	EPI_ISL_338256
A/South_Australia/238/2018|Australia|12/15/2018	Australia	12/15/2018	6b1.A/183P6	Australia	EPI_ISL_339224
A/South_Australia/227/2018|Australia|12/10/2018	Australia	12/10/2018	6b1.A/183P6	Australia	EPI_ISL_339223
A/South_Australia/221/2018|Australia|12/14/2018	Australia	12/14/2018	6b1.A/183P6	Australia	EPI_ISL_339263

A/South_Australia/173/2018|Australia|11/03/2018	Australia	11/03/2018	6b1.A/183P6	Australia	EPI_ISL_334432
A/South_Australia/196/2018|Australia|11/18/2018	Australia	11/18/2018	6b1.A/183P6	Australia	EPI_ISL_334082
A/South_Australia/240/2018|Australia|12/17/2018	Australia	12/17/2018	6b1.A/183P6	Australia	EPI_ISL_339225
A/South_Australia/188/2018|Australia|11/19/2018	Australia	11/19/2018	6b1.A/183P6	Australia	EPI_ISL_336010
A/Victoria/2076/2018|Australia|09/28/2018	Australia	09/28/2018	6b1.A/183P6	Australia	EPI_ISL_332756
A/Tasmania/11/2018|Australia|11/20/2018	Australia	11/20/2018	6b1.A/183P6	Australia	EPI_ISL_334083
A/Victoria/1001/2018|Australia|09/07/2018	Australia	09/07/2018	6b1.A/183P6	Australia	EPI_ISL_330099
A/Victoria/2045/2018|Australia|09/01/2018	Australia	09/01/2018	6b1.A/183P6	Australia	EPI_ISL_330110
A/Victoria/744/2018|Australia|11/25/2018	Australia	11/25/2018	6b1.A/183P6	Australia	EPI_ISL_338250
A/Victoria/1005/2018|Australia|10/18/2018	Australia	10/18/2018	6b1.A/183P6	Australia	EPI_ISL_332067
A/Sydney/1034/2018|Australia|10/02/2018	Australia	10/02/2018	6b1.A/183P6	Australia	EPI_ISL_332773
A/Victoria/2162/2018|Australia|11/29/2018	Australia	11/29/2018	6b1.A/183P6	Australia	EPI_ISL_339231
A/Newcastle/619/2019|Australia|03/25/2019	Australia	03/25/2019	6b1.A/183P6	Australia	EPI_ISL_355186
A/Victoria/2022/2019|Australia|01/13/2019	Australia	01/13/2019	6b1.A/183P6	Australia	EPI_ISL_354420
A/Sydney/41/2019|Australia|03/12/2019	Australia	03/12/2019	6b1.A/183P6	Australia	EPI_ISL_354709
A/Sydney/40/2019|Australia|03/12/2019	Australia	03/12/2019	6b1.A/183P6	Australia	EPI_ISL_354769
A/Newcastle/31/2019|Australia|03/12/2019	Australia	03/12/2019	6b1.A/183P6	Australia	EPI_ISL_355187
A/Sydney/713/2019|Australia|02/03/2019	Australia	02/03/2019	6b1.A/183P6	Australia	EPI_ISL_354472
A/Newcastle/16/2019|Australia|01/19/2019	Australia	01/19/2019	6b1.A/183P6	Australia	EPI_ISL_341427
A/Brisbane/169/2018|Australia|11/13/2018	Australia	11/13/2018	6b1.A/183P6	Australia	EPI_ISL_339198
A/Brisbane/157/2018|Australia|10/18/2018	Australia	10/18/2018	6b1.A/183P6	Australia	EPI_ISL_334434
A/South_Australia/122/2019|Australia|03/06/2019	Australia	03/06/2019	6b1.A/183P6	Australia	EPI_ISL_354711
A/Victoria/2082/2019|Australia|02/14/2019	Australia	02/14/2019	6b1.A/183P6	Australia	EPI_ISL_354505
A/Newcastle/85/2018|Australia|12/27/2018	Australia	12/27/2018	6b1.A/183P6	Australia	EPI_ISL_339051
A/Newcastle/119/2018|Australia|12/17/2018	Australia	12/17/2018	6b1.A/183P6	Australia	EPI_ISL_339192
A/Newcastle/100/2018|Australia|11/28/2018	Australia	11/28/2018	6b1.A/183P6	Australia	EPI_ISL_339174
A/Newcastle/101/2018|Australia|12/15/2018	Australia	12/15/2018	6b1.A/183P6	Australia	EPI_ISL_339189
A/Newcastle/77/2018|Australia|12/28/2018	Australia	12/28/2018	6b1.A/183P6	Australia	EPI_ISL_336002
A/Victoria/2137/2018|Australia|11/06/2018	Australia	11/06/2018	6b1.A/183P6	Australia	EPI_ISL_339218
A/Sydney/77/2018|Australia|07/21/2018	Australia	07/21/2018	6b1.A/183P6		EPI_ISL_322755
A/Sydney/144/2018|Australia|09/24/2018	Australia	09/24/2018	6b1.A/183P6	Australia	EPI_ISL_332751
A/Sydney/31/2018|Australia|03/22/2018	Australia	03/22/2018	6b1.A/183P6		EPI_ISL_320395
A/Brisbane/59/2018|Australia|05/10/2018	Australia	05/10/2018	6b1.A/183P6		EPI_ISL_322330
A/Perth/1011/2018|Australia|07/11/2018	Australia	07/11/2018	6b1.A/183P6		EPI_ISL_322985

A/Perth/1002/2018|Australia|06/26/2018	Australia	06/26/2018	6b1.A/183P6		EPI_ISL_323066
A/Brisbane/178/2018|Australia|12/14/2018	Australia	12/14/2018	6b1.A/183P6	Australia	EPI_ISL_339200
A/Perth/1025/2018|Australia|09/11/2018	Australia	09/11/2018	6b1.A/183P6	Australia	EPI_ISL_331298
A/Perth/57/2018|Australia|08/07/2018	Australia	08/07/2018	6b1.A/183P6		EPI_ISL_322775
A/Victoria/906/2018|Australia|09/24/2018	Australia	09/24/2018	6b1.A/183P6	Australia	EPI_ISL_332068
A/Victoria/2044/2018|Australia|08/30/2018	Australia	08/30/2018	6b1.A/183P6		EPI_ISL_330107
A/Brisbane/122/2018|Australia|08/06/2018	Australia	08/06/2018	6b1.A/183P6		EPI_ISL_322772
A/Perth/2/2019|Australia|01/13/2019	Australia	01/13/2019	6b1.A/183P6	Australia	EPI_ISL_339282
A/Newcastle/103/2018|Australia|12/04/2018	Australia	12/04/2018	6b1.A/183P6	Australia	EPI_ISL_339191
A/Tasmania/2/2018|Australia|10/01/2018	Australia	10/01/2018	6b1.A/183P6	Australia	EPI_ISL_332759
A/Sydney/134/2018|Australia|09/03/2018	Australia	09/03/2018	6b1.A/183P6	Australia	EPI_ISL_326769
A/South_Australia/25/2018|Australia|08/15/2018	Australia	08/15/2018	6b1.A/183P6		EPI_ISL_326773
A/Tasmania/505/2018|Australia|10/18/2018	Australia	10/18/2018	6b1.A/183P6	Australia	EPI_ISL_336001
A/Newcastle/49/2018|Australia|10/02/2018	Australia	10/02/2018	6b1.A/183P6	Australia	EPI_ISL_332762
A/Brisbane/167/2018|Australia|11/08/2018	Australia	11/08/2018	6b1.A/183P6	Australia	EPI_ISL_339204
A/South_Australia/17/2018|Australia|06/27/2018	Australia	06/27/2018	6b1.A/183P6		EPI_ISL_323068
A/Newcastle/30/2018|Australia|07/19/2018	Australia	07/19/2018	6b1.A/183P6		EPI_ISL_322747
A/South_Australia/58/2018|Australia|09/14/2018	Australia	09/14/2018	6b1.A/183P6	Australia	EPI_ISL_330102
A/South_Australia/1019/2018|Australia|10/02/2018	Australia	10/02/2018	6b1.A/183P6	Australia	EPI_ISL_332771
A/Canberra/29/2019|Australia|02/09/2019	Australia	02/09/2019	6b1.A/183P6	Australia	EPI_ISL_354480
A/Sydney/1005/2018|Australia|07/09/2018	Australia	07/09/2018	6b1.A/183P6		EPI_ISL_320944
A/Sydney/512/2018|Australia|06/28/2018	Australia	06/28/2018	6b1.A/183P6		EPI_ISL_323003
A/South_Australia/14/2018|Australia|06/05/2018	Australia	06/05/2018	6b1.A/183P6		EPI_ISL_320408
A/South_Australia/15/2018|Australia|06/17/2018	Australia	06/17/2018	6b1.A/183P6		EPI_ISL_320409
A/Brisbane/123/2018|Australia|08/03/2018	Australia	08/03/2018	6b1.A/183P6		EPI_ISL_322773
A/Sydney/713/2018|Australia|07/18/2018	Australia	07/18/2018	6b1.A/183P6		EPI_ISL_322800
A/Sydney/50/2018|Australia|06/17/2018	Australia	06/17/2018	6b1.A/183P6		EPI_ISL_323062
A/Victoria/2102/2018|Australia|10/08/2018	Australia	10/08/2018	6b1.A/183P6	Australia	EPI_ISL_338255
A/Newcastle/3/2018|Australia|02/09/2018	Australia	02/09/2018	6b1.A/183P6		EPI_ISL_312212
A/Newcastle/1/2018|Australia|02/06/2018	Australia	02/06/2018	6b1.A/183P6		EPI_ISL_312186
A/Sydney/728/2018|Australia|08/01/2018	Australia	08/01/2018	6b1.A/183P1		EPI_ISL_323084
A/Canberra/25/2018|Australia|07/25/2018	Australia	07/25/2018	6b1.A/183P1		EPI_ISL_326785
A/Sydney/519/2018|Australia|07/23/2018	Australia	07/23/2018	6b1.A/183P1		EPI_ISL_323075
A/Sydney/70/2018|Australia|07/19/2018	Australia	07/19/2018	6b1.A/183P1		EPI_ISL_326782

A/Sydney/40/2018|Australia|07/09/2018	Australia	07/09/2018	6b1.A/183P1		EPI_ISL_320950
A/Sydney/707/2018|Australia|07/08/2018	Australia	07/08/2018	6b1.A/183P1		EPI_ISL_322798
A/Sydney/39/2018|Australia|07/03/2018	Australia	07/03/2018	6b1.A/183P1		EPI_ISL_321476
A/Sydney/59/2018|Australia|06/25/2018	Australia	06/25/2018	6b1.A/183P1		EPI_ISL_323064
A/Sydney/54/2018|Australia|06/21/2018	Australia	06/21/2018	6b1.A/183P1		EPI_ISL_323063
A/Sydney/51/2018|Australia|06/21/2018	Australia	06/21/2018	6b1.A/183P1		EPI_ISL_321475
A/Sydney/1016/2018|Australia|08/14/2018	Australia	08/14/2018	6b1.A/183P1		EPI_ISL_322780
A/Sydney/711/2018|Australia|07/16/2018	Australia	07/16/2018	6b1.A/183P1		EPI_ISL_322799
A/Sydney/704/2018|Australia|07/04/2018	Australia	07/04/2018	6b1.A/183P1		EPI_ISL_322797
A/Sydney/76/2018|Australia|07/24/2018	Australia	07/24/2018	6b1.A/183P1		EPI_ISL_322754
A/Sydney/500/2018|Australia|07/30/2018	Australia	07/30/2018	6b1.A/183P1		EPI_ISL_323001
A/Sydney/533/2018|Australia|07/16/2018	Australia	07/16/2018	6b1.A/183P1		EPI_ISL_323074
A/Sydney/180/2018|Australia|10/10/2018	Australia	10/10/2018	6b1.A/183P1	Australia	EPI_ISL_332770
A/Canberra/1001/2018|Australia|09/05/2018	Australia	09/05/2018	6b1.A/183P1	Australia	EPI_ISL_330100
A/Sydney/1009/2018|Australia|07/20/2018	Australia	07/20/2018	6b1.A/183P1		EPI_ISL_322986
A/Sydney/64/2018|Australia|07/10/2018	Australia	07/10/2018	6b1.A/183P1		EPI_ISL_322028
A/Sydney/42/2018|Australia|07/09/2018	Australia	07/09/2018	6b1.A/183P1		EPI_ISL_319654
A/Sydney/44/2018|Australia|07/08/2018	Australia	07/08/2018	6b1.A/183P1		EPI_ISL_319653
A/Sydney/701/2018|Australia|07/02/2018	Australia	07/02/2018	6b1.A/183P1		EPI_ISL_322796
A/Newcastle/24/2018|Australia|06/20/2018	Australia	06/20/2018	6b1.A/183P1		EPI_ISL_322729
A/Canberra/26/2018|Australia|07/25/2018	Australia	07/25/2018	6b1.A/183P1		EPI_ISL_322031
A/Canberra/22/2018|Australia|07/19/2018	Australia	07/19/2018	6b1.A/183P1		EPI_ISL_323014
A/Sydney/45/2018|Australia|07/08/2018	Australia	07/08/2018	6b1.A/183P1		EPI_ISL_321477
A/Canberra/24/2018|Australia|07/20/2018	Australia	07/20/2018	6b1.A/183P1		EPI_ISL_323008
A/Newcastle/45/2018|Australia|08/09/2018	Australia	08/09/2018	6b1.A/183P1		EPI_ISL_332490
A/Newcastle/122/2018|Australia|12/14/2018	Australia	12/14/2018	6b1.A/183P1	Australia	EPI_ISL_339193
A/Sydney/65/2018|Australia|07/04/2018	Australia	07/04/2018	6b1.A/183P1		EPI_ISL_323011
A/Brisbane/02/2018|Australia|01/04/2018	Australia	01/04/2018	6b1.A/183P1		EPI_ISL_362099
A/Brisbane/02/2018|Australia|04/11/2019	Australia	04/11/2019	6b1.A/183P1	Australia	EPI_ISL_362099
A/Brisbane/93/2018|Australia|07/05/2018	Australia	07/05/2018	6b1.A/183P1		EPI_ISL_322029
A/Sydney/1001/2019|Australia|01/14/2019	Australia	01/14/2019	6b1.A/183P1	Australia	EPI_ISL_345233
A/Sydney/1003/2019|Australia|01/11/2019	Australia	01/11/2019	6b1.A/183P1	Australia	EPI_ISL_345232
A/Perth/70/2018|Australia|08/12/2018	Australia	08/12/2018	6b1.A/183P1		EPI_ISL_326778
A/Perth/63/2018|Australia|08/04/2018	Australia	08/04/2018	6b1.A/183P1		EPI_ISL_326775

A/Newcastle/25/2018|Australia|06/22/2018	Australia	06/22/2018	6b1.A/183P1		EPI_ISL_326783
A/Townsville/12/2018|Australia|06/16/2018	Australia	06/16/2018	6b1.A/183P1		EPI_ISL_323071
A/Perth/1006/2018|Australia|07/11/2018	Australia	07/11/2018	6b1.A/183P1		EPI_ISL_320945
A/Perth/34/2018|Australia|06/23/2018	Australia	06/23/2018	6b1.A/183P1		EPI_ISL_323070
A/South_Australia/18/2018|Australia|07/02/2018	Australia	07/02/2018	6b1.A/183P1		EPI_ISL_323069
A/Townsville/9/2019|Australia|02/18/2019	Australia	02/18/2019	6b1.A/183P1	Australia	EPI_ISL_354468
A/Newcastle/149/2018|Australia|12/14/2018	Australia	12/14/2018	6b1.A/183P1	Australia	EPI_ISL_339203
A/Brisbane/1/2019|Australia|01/02/2019	Australia	01/02/2019	6b1.A/183P1	Australia	EPI_ISL_339201
A/Brisbane/1018/2018|Australia|10/05/2018	Australia	10/05/2018	6b1.A/183P1	Australia	EPI_ISL_332774
A/Newcastle/8/2019|Australia|01/02/2019	Australia	01/02/2019	6b1.A/183P1	Australia	EPI_ISL_339344
A/Victoria/742/2018|Australia|11/16/2018	Australia	11/16/2018	6b1.A/183P1	Australia	EPI_ISL_339182
A/Sydney/9/2018|Australia|01/23/2018	Australia	01/23/2018	6b1.A/183P1		EPI_ISL_312209
A/Townsville/06/2018|Australia|04/14/2018	Australia	04/14/2018	6b1.A/183P1		EPI_ISL_314810
A/South_Australia/1025/2018|Australia|10/02/2018	Australia	10/02/2018	6b1.A/183P1	Australia	EPI_ISL_332772
A/South_Australia/27/2018|Australia|08/13/2018	Australia	08/13/2018	6b1.A/183P1		EPI_ISL_332491
A/Victoria/9/2018|Australia|08/15/2018	Australia	08/15/2018	6b1.A/183P1		EPI_ISL_322776
A/Brisbane/121/2018|Australia|08/06/2018	Australia	08/06/2018	6b1.A/183P1		EPI_ISL_323091
A/Brisbane/61/2018|Australia|05/17/2018	Australia	05/17/2018	6b1.A/183P1		EPI_ISL_314812
A/Newcastle/47/2018|Australia|08/10/2018	Australia	08/10/2018	6b1.A/183P2		EPI_ISL_323089
A/South_Australia/23/2018|Australia|08/07/2018	Australia	08/07/2018	6b1.A/183P2		EPI_ISL_322781
A/Brisbane/68/2018|Australia|06/06/2018	Australia	06/06/2018	6b1.A/183P2		EPI_ISL_322321
A/Brisbane/69/2018|Australia|05/30/2018	Australia	05/30/2018	6b1.A/183P2		EPI_ISL_320412
A/Brisbane/65/2018|Australia|05/29/2018	Australia	05/29/2018	6b1.A/183P2		EPI_ISL_320420
A/Sydney/169/2018|Australia|09/18/2018	Australia	09/18/2018	6b1.A/183P2	Australia	EPI_ISL_332748
A/South_Australia/1011/2018|Australia|07/24/2018	Australia	07/24/2018	6b1.A/183P2		EPI_ISL_322987
A/South_Australia/1013/2018|Australia|08/06/2018	Australia	08/06/2018	6b1.A/183P2		EPI_ISL_322778
A/Canberra/21/2018|Australia|07/19/2018	Australia	07/19/2018	6b1.A/183P2		EPI_ISL_323072
A/Sydney/501/2018|Australia|07/25/2018	Australia	07/25/2018	6b1.A/183P2		EPI_ISL_323002
A/Sydney/723/2018|Australia|07/25/2018	Australia	07/25/2018	6b1.A/183P2		EPI_ISL_322802
A/South_Australia/1012/2018|Australia|07/24/2018	Australia	07/24/2018	6b1.A/183P2		EPI_ISL_322988
A/Newcastle/29/2018|Australia|07/19/2018	Australia	07/19/2018	6b1.A/183P2		EPI_ISL_322731
A/Sydney/181/2018|Australia|10/11/2018	Australia	10/11/2018	6b1.A/183P2	Australia	EPI_ISL_332769
A/South_Australia/246/2018|Australia|12/27/2018	Australia	12/27/2018	6b1.A/183P2	Australia	EPI_ISL_339226
A/South_Australia/202/2018|Australia|11/25/2018	Australia	11/25/2018	6b1.A/183P2	Australia	EPI_ISL_339221

A/South_Australia/193/2018|Australia|11/23/2018	Australia	11/23/2018	6b1.A/183P2	Australia	EPI_ISL_334081
A/Victoria/251/2018|Australia|10/03/2018	Australia	10/03/2018	6b1.A/183P2	Australia	EPI_ISL_331297
A/Victoria/2151/2018|Australia|11/21/2018	Australia	11/21/2018	6b1.A/183P2	Australia	EPI_ISL_339219
A/Sydney/131/2018|Australia|09/01/2018	Australia	09/01/2018	6b1.A/183P2	Australia	EPI_ISL_326768
A/Sydney/127/2018|Australia|08/30/2018	Australia	08/30/2018	6b1.A/183P2		EPI_ISL_326767
A/Sydney/126/2018|Australia|08/25/2018	Australia	08/25/2018	6b1.A/183P2		EPI_ISL_326766
A/Tasmania/4/2018|Australia|10/03/2018	Australia	10/03/2018	6b1.A/183P2	Australia	EPI_ISL_332760
A/Tasmania/5/2018|Australia|10/03/2018	Australia	10/03/2018	6b1.A/183P2	Australia	EPI_ISL_332761
A/Townsville/08/2018|Australia|05/23/2018	Australia	05/23/2018	6b1.A/183P2		EPI_ISL_322322
A/Townsville/21/2018|Australia|08/02/2018	Australia	08/02/2018	6b1.A/183P2		EPI_ISL_323090
A/Townsville/07/2018|Australia|05/17/2018	Australia	05/17/2018	6b1.A/183P2		EPI_ISL_320419
A/Brisbane/48/2018|Australia|04/23/2018	Australia	04/23/2018	6b1.A/183P2		EPI_ISL_320386
A/South_Australia/132/2019|Australia|03/01/2019	Australia	03/01/2019	6b1.A/183P2	Australia	EPI_ISL_354706
A/Brisbane/174/2018|Australia|12/03/2018	Australia	12/03/2018	6b1.A/183P2	Australia	EPI_ISL_339199
A/Townsville/09/2018|Australia|05/15/2018	Australia	05/15/2018	6b1.A/183P2		EPI_ISL_320411
A/Newcastle/35/2018|Australia|07/25/2018	Australia	07/25/2018	6b1.A/183P2		EPI_ISL_322748
A/Canberra/23/2018|Australia|07/19/2018	Australia	07/19/2018	6b1.A/183P2		EPI_ISL_321470
A/Sydney/75/2018|Australia|07/08/2018	Australia	07/08/2018	6b1.A/183P2		EPI_ISL_322765
A/Canberra/20/2018|Australia|07/08/2018	Australia	07/08/2018	6b1.A/183P2		EPI_ISL_323013
A/Sydney/60/2018|Australia|07/08/2018	Australia	07/08/2018	6b1.A/183P2		EPI_ISL_323065
A/Sydney/80/2018|Australia|07/06/2018	Australia	07/06/2018	6b1.A/183P2		EPI_ISL_322756
A/Sydney/66/2018|Australia|07/03/2018	Australia	07/03/2018	6b1.A/183P2		EPI_ISL_323073
A/Victoria/10/2018|Australia|08/18/2018	Australia	08/18/2018	6b1.A/183P2		EPI_ISL_322777
A/South_Australia/139/2018|Australia|10/24/2018	Australia	10/24/2018	6b1.A/183P2	Australia	EPI_ISL_332777
A/Townsville/11/2018|Australia|06/06/2018	Australia	06/06/2018	6b1.A/183P2		EPI_ISL_322323
A/Newcastle/42/2018|Australia|08/05/2018	Australia	08/05/2018	6b1.A/183P2		EPI_ISL_323088
A/Newcastle/43/2018|Australia|08/06/2018	Australia	08/06/2018	6b1.A/183P2		EPI_ISL_323047
A/Newcastle/40/2018|Australia|08/03/2018	Australia	08/03/2018	6b1.A/183P2		EPI_ISL_323087
A/Newcastle/32/2018|Australia|07/24/2018	Australia	07/24/2018	6b1.A/183P2		EPI_ISL_323086
A/Newcastle/28/2018|Australia|07/17/2018	Australia	07/17/2018	6b1.A/183P2		EPI_ISL_323085
A/Townsville/22/2018|Australia|08/05/2018	Australia	08/05/2018	6b1.A/183P2		EPI_ISL_323092
A/Sydney/132/2018|Australia|09/02/2018	Australia	09/02/2018	6b1.A/183P2	Australia	EPI_ISL_330109
A/Tasmania/515/2018|Australia|10/28/2018	Australia	10/28/2018	6b1.A/183P2	Australia	EPI_ISL_332065
A/Newcastle/74/2018|Australia|09/16/2018	Australia	09/16/2018	6b1.A/183P2	Australia	EPI_ISL_332749

A/Townsville/14/2018|Australia|06/15/2018	Australia	06/15/2018	6b1.A/183P2		EPI_ISL_320946
A/Victoria/2066/2018|Australia|09/20/2018	Australia	09/20/2018	6b1.A/183P2	Australia	EPI_ISL_332755
A/Victoria/2040/2018|Australia|08/23/2018	Australia	08/23/2018	6b1.A/183P2		EPI_ISL_330106
A/Newcastle/88/2018|Australia|11/07/2018	Australia	11/07/2018	6b1.A/183P2	Australia	EPI_ISL_336004
A/Brisbane/41/2018|Australia|04/10/2018	Australia	04/10/2018	6b1.A/183P2		EPI_ISL_314815
A/Brisbane/1006/2018|Australia|08/13/2018	Australia	08/13/2018	6b1.A/183P2		EPI_ISL_326772
A/Brisbane/159/2018|Australia|10/25/2018	Australia	10/25/2018	6b1.A/183P2	Australia	EPI_ISL_338254
A/Brisbane/72/2018|Australia|06/11/2018	Australia	06/11/2018	6b1.A/183P2		EPI_ISL_322325
A/Brisbane/71/2018|Australia|06/06/2018	Australia	06/06/2018	6b1.A/183P2		EPI_ISL_320413
A/Tasmania/502/2018|Australia|10/16/2018	Australia	10/16/2018	6b1.A/183P2	Australia	EPI_ISL_332778
A/Tasmania/518/2018|Australia|10/14/2018	Australia	10/14/2018	6b1.A/183P2	Australia	EPI_ISL_332070
A/Brisbane/52/2018|Australia|05/02/2018	Australia	05/02/2018	6b1.A/183P2		EPI_ISL_320387
A/Brisbane/133/2018|Australia|08/29/2018	Australia	08/29/2018	6b1.A/183P2		EPI_ISL_331296
A/Brisbane/78/2018|Australia|06/18/2018	Australia	06/18/2018	6b1.A/183P2		EPI_ISL_320947
A/Newcastle/102/2018|Australia|11/18/2018	Australia	11/18/2018	6b1.A/183P2	Australia	EPI_ISL_339190
A/Brisbane/70/2018|Australia|06/06/2018	Australia	06/06/2018	6b1.A/183P2		EPI_ISL_322324
A/Victoria/22/2018|Australia|09/26/2018	Australia	09/26/2018	6b1.A/183P2	Australia	EPI_ISL_331301
A/Victoria/19/2018|Australia|09/18/2018	Australia	09/18/2018	6b1.A/183P2	Australia	EPI_ISL_331299
A/South_Australia/24/2018|Australia|08/14/2018	Australia	08/14/2018	6b1.A/183P2		EPI_ISL_322782
A/Brisbane/151/2018|Australia|10/10/2018	Australia	10/10/2018	6b1.A/183P2	Australia	EPI_ISL_338257
A/Perth/40/2018|Australia|06/30/2018	Australia	06/30/2018	6b1.A/183P2		EPI_ISL_323012
A/South_Australia/1008/2018|Australia|07/15/2018	Australia	07/15/2018	6b1.A/183P2		EPI_ISL_323005
A/Perth/47/2018|Australia|08/01/2018	Australia	08/01/2018	6b1.A/183P2		EPI_ISL_326774
A/Perth/1016/2018|Australia|07/31/2018	Australia	07/31/2018	6b1.A/183P2		EPI_ISL_326771
A/Perth/1010/2018|Australia|07/12/2018	Australia	07/12/2018	6b1.A/183P2		EPI_ISL_323004
A/Newcastle/46/2019|Australia|03/13/2019	Australia	03/13/2019	6b1.A/183P2	Australia	EPI_ISL_362102
A/Newcastle/48/2019|Australia|03/17/2019	Australia	03/17/2019	6b1.A/183P2	Australia	EPI_ISL_354693
A/Canberra/13/2019|Australia|01/19/2019	Australia	01/19/2019	6b1.A/183P2	Australia	EPI_ISL_362100
A/Darwin/102/2019|Australia|03/21/2019	Australia	03/21/2019	6b1.A/183P2	Australia	EPI_ISL_354701
A/Victoria/2158/2018|Australia|11/25/2018	Australia	11/25/2018	6b1.A/183P2	Australia	EPI_ISL_341432
A/South_Australia/239/2018|Australia|12/15/2018	Australia	12/15/2018	6b1.A/183P2	Australia	EPI_ISL_341431
A/Brisbane/47/2018|Australia|04/07/2018	Australia	04/07/2018	6b1.A/183P2		EPI_ISL_320415
A/Victoria/2128/2018|Australia|11/02/2018	Australia	11/02/2018	6b1.A/183P2	Australia	EPI_ISL_339227
A/Sydney/16/2018|Australia|03/08/2018	Australia	03/08/2018	6b1.A/183P2		EPI_ISL_320396

A/Newcastle/76/2018|Australia|12/05/2018	Australia	12/05/2018	6b1.A/183P3	Australia	EPI_ISL_339049
A/South_Australia/42/2019|Australia|02/07/2019	Australia	02/07/2019	6b1.A/183P7	Australia	EPI_ISL_351754
A/Sydney/178/2018|Australia|10/04/2018	Australia	10/04/2018	6b1.A/183P7	Australia	EPI_ISL_332752
A/Victoria/2117/2018|Australia|10/22/2018	Australia	10/22/2018	6b1.A/183P7	Australia	EPI_ISL_338276
A/Newcastle/92/2018|Australia|10/25/2018	Australia	10/25/2018	6b1.A/183P7	Australia	EPI_ISL_339173
A/Victoria/700/2019|Australia|03/10/2019	Australia	03/10/2019	6b1.A/183P7	Australia	EPI_ISL_348328
A/Victoria/2157/2018|Australia|11/24/2018	Australia	11/24/2018	6b1.A/183P7	Australia	EPI_ISL_339220
A/Brisbane/03/2018|Australia|01/04/2018	Australia	01/04/2018	6b1.A/183P7		EPI_ISL_312215
A/Sydney/90/2018|Australia|06/24/2018	Australia	06/24/2018	6b1.A/183P7		EPI_ISL_322766
A/Perth/14/2018|Australia|02/15/2018	Australia	02/15/2018	6b1.A/183P7		EPI_ISL_312217
A/Perth/1005/2018|Australia|07/10/2018	Australia	07/10/2018	6b1.A/183P7		EPI_ISL_323067
A/Perth/10/2018|Australia|08/19/2018	Australia	08/19/2018	6b1.A/183P7		EPI_ISL_326776
A/Perth/21/2018|Australia|03/29/2018	Australia	03/29/2018	6b1.A/183P7		EPI_ISL_320394
A/Darwin/10/2018|Australia|11/07/2018	Australia	11/07/2018	6b1.A	Australia	EPI_ISL_332060
A/Darwin/9/2018|Australia|11/08/2018	Australia	11/08/2018	6b1.A	Australia	EPI_ISL_332062
A/Perth/183/2018|Australia|11/01/2018	Australia	11/01/2018	6b1.A	Australia	EPI_ISL_334433
A/Sydney/61/2018|Australia|07/07/2018	Australia	07/07/2018	6b1.A		EPI_ISL_320943
A/Newcastle/10/2018|Australia|03/25/2018	Australia	03/25/2018	6b1.A		EPI_ISL_320370
A/Sydney/718/2018|Australia|07/21/2018	Australia	07/21/2018	6b1.A		EPI_ISL_326781
A/Canberra/15/2018|Australia|06/29/2018	Australia	06/29/2018	6b1.A		EPI_ISL_321449
A/Canberra/14/2018|Australia|06/29/2018	Australia	06/29/2018	6b1.A		EPI_ISL_322030
A/Brisbane/46/2018|Australia|04/22/2018	Australia	04/22/2018	6b1.A		EPI_ISL_320385
A/Darwin/59/2018|Australia|11/21/2018	Australia	11/21/2018	6b1.A	Australia	EPI_ISL_334002
A/Townsville/26/2018|Australia|08/08/2018	Australia	08/08/2018	6b1.A		EPI_ISL_331300
A/Sydney/6/2018|Australia|01/28/2018	Australia	01/28/2018	6b1.A		EPI_ISL_314059
A/Brisbane/21/2018|Australia|02/17/2018	Australia	02/17/2018	6b1.A		EPI_ISL_312214
A/Townsville/01/2018|Australia|02/10/2018	Australia	02/10/2018	6b1.A		EPI_ISL_312213
A/Perth/10/2018|Australia|02/22/2018	Australia	02/22/2018	6b1.A		EPI_ISL_326776
A/Perth/6/2018|Australia|02/04/2018	Australia	02/04/2018	6b1.A		EPI_ISL_312216
A/Perth/9/2018|Australia|02/17/2018	Australia	02/17/2018	6b1.A		EPI_ISL_314060
A/Sydney/2/2018|Australia|01/04/2018	Australia	01/04/2018	6b1.A		EPI_ISL_312208
A/Bari/424/2019|Europe|04/03/2019	Europe	04/03/2019	6b1.A/183P5	Global	EPI_ISL_356735
A/Sachsen/111/2019|Europe|04/02/2019	Europe	04/02/2019	6b1.A/183P5	Global	EPI_ISL_355943
A/Stockholm/21/2018|Europe|09/30/2018	Europe	09/30/2018	6b1.A/183P5	Global	EPI_ISL_332333

A/Romania/240760/2019|Europe|01/23/2019	Europe	01/23/2019	6b1.A/183P5	Global	EPI_ISL_347607
A/England/619/2018|Europe|11/17/2018	Europe	11/17/2018	6b1.A/183P5	Global	EPI_ISL_335393
A/Neath/6337/2018|Europe|10/28/2018	Europe	10/28/2018	6b1.A/183P5	Global	EPI_ISL_331244
A/Llanelli/0234/2018|Europe|10/28/2018	Europe	10/28/2018	6b1.A/183P5	Global	EPI_ISL_331249
A/Swansea/8921/2018|Europe|11/14/2018	Europe	11/14/2018	6b1.A/183P5	Global	EPI_ISL_332134
A/Vologda/384/2019|Europe|02/28/2019	Europe	02/28/2019	6b1.A/183P5	Global	EPI_ISL_352144
A/Nordrhein-Westfalen/120/2019|Europe|04/04/2019	Europe	04/04/2019	6b1.A/183P5	Global	EPI_ISL_356687
A/Sweden/32/2019|Europe|03/07/2019	Europe	03/07/2019	6b1.A/183P5	Global	EPI_ISL_347817
A/Norway/3679/2018|Europe|11/29/2018	Europe	11/29/2018	6b1.A/183P5	Global	EPI_ISL_347403
A/Irkutsk/975/2019|Europe|02/15/2019	Europe	02/15/2019	6b1.A/183P5	Global	EPI_ISL_352157
A/Netherlands/10201/2019|Europe|03/01/2019	Europe	03/01/2019	6b1.A/183P5	Global	EPI_ISL_349477
A/Finland/103/2019|Europe|04/02/2019	Europe	04/02/2019	6b1.A/183P5	Global	EPI_ISL_357780
A/Catalonia/2053646NS/2018|Europe|12/27/2018	Europe	12/27/2018	6b1.A/183P5	Global	EPI_ISL_335217
A/Netherlands/00348/2019|Europe|02/12/2019	Europe	02/12/2019	6b1.A/183P5	Global	EPI_ISL_342163
A/Denmark/2824/2019|Europe|03/18/2019	Europe	03/18/2019	6b1.A/183P5	Global	EPI_ISL_354061
A/Finland/981/2018|Europe|12/12/2018	Europe	12/12/2018	6b1.A/183P5	Global	EPI_ISL_337404
A/Andalucia/268/2019|Europe|01/05/2019	Europe	01/05/2019	6b1.A/183P5	Global	EPI_ISL_355263
A/Finland/39/2019|Europe|02/17/2019	Europe	02/17/2019	6b1.A/183P5	Global	EPI_ISL_357783
A/Gothenburg/1/2018|Europe|09/05/2018	Europe	09/05/2018	6b1.A/183P5	Global	EPI_ISL_329742
A/Lisboa/5/2018|Europe|01/26/2018	Europe	01/26/2018	6b1.A/183P5		EPI_ISL_320003
A/Moscow/84/2018|Europe|03/14/2018	Europe	03/14/2018	6b1.A/183P5		EPI_ISL_322382
A/Netherlands/01121/18|Europe|02/17/2018	Europe	02/17/2018	6b1.A/183P5		EPI_ISL_305437
A/Belgium/G0021/2018|Europe|01/03/2018	Europe	01/03/2018	6b1.A/183P5		EPI_ISL_298763
A/Bretagne/002/2018|Europe|01/02/2018	Europe	01/02/2018	6b1.A/183P5		EPI_ISL_294131
A/Kalmar/1/2018|Europe|07/29/2018	Europe	07/29/2018	6b1.A/183P5		EPI_ISL_329739
A/Ireland/90237/2018|Europe|12/19/2018	Europe	12/19/2018	6b1.A/183P6	Global	EPI_ISL_337055
A/Norway/1899/2019|Europe|05/02/2019	Europe	05/02/2019	6b1.A/183P6	Global	EPI_ISL_357442
A/Norway/3224/2018|Europe|07/31/2018	Europe	07/31/2018	6b1.A/183P6		EPI_ISL_321781
A/Norway/3221/2018|Europe|07/24/2018	Europe	07/24/2018	6b1.A/183P6		EPI_ISL_321779
A/Valladolid/559/2018|Europe|09/24/2018	Europe	09/24/2018	6b1.A/183P6	Global	EPI_ISL_347449
A/Estonia/112776/2018|Europe|03/02/2018	Europe	03/02/2018	6b1.A/183P6		EPI_ISL_320815
A/Moscow/122/2018|Europe|04/03/2018	Europe	04/03/2018	6b1.A/183P6		EPI_ISL_322372
A/Saint-Petersburg/RII-291/2018|Europe|03/17/2018	Europe	03/17/2018	6b1.A/183P6		EPI_ISL_322378
A/Sassari/07/2018|Europe|01/22/2018	Europe	01/22/2018	6b1.A/183P6		EPI_ISL_311994

A/Romania/223921/2018|Europe|02/14/2018	Europe	02/14/2018	6b1.A/183P6		EPI_ISL_312670
A/Norway/2947/2018|Europe|05/22/2018	Europe	05/22/2018	6b1.A/183P6		EPI_ISL_321775
A/Moscow/134/2018|Europe|04/11/2018	Europe	04/11/2018	6b1.A/183P6		EPI_ISL_322371
A/Saint-Petersburg/RII-230/2018|Europe|03/04/2018	Europe	03/04/2018	6b1.A/183P6		EPI_ISL_322418
A/Northern_Ireland/16810/2018|Europe|05/21/2018	Europe	05/21/2018	6b1.A/183P6		EPI_ISL_329757
A/Voronezh/CRIE/59/2018|Europe|05/05/2018	Europe	05/05/2018	6b1.A/183P1		EPI_ISL_316159
A/England/591/2018|Europe|07/15/2018	Europe	07/15/2018	6b1.A/183P1		EPI_ISL_335403
A/Olt/225477/2018|Europe|03/03/2018	Europe	03/03/2018	6b1.A/183P1		EPI_ISL_332841
A/Izhevsk/RII-1689S/2018|Europe|02/06/2018	Europe	02/06/2018	6b1.A/183P1		EPI_ISL_322343
A/St_Petersburg/RII-477/2018|Europe|05/03/2018	Europe	05/03/2018	6b1.A/183P1		EPI_ISL_330040
A/Norway/3231/2018|Europe|08/08/2018	Europe	08/08/2018	6b1.A/183P1		EPI_ISL_321783
A/Khabarovsk/35/2018|Europe|04/04/2018	Europe	04/04/2018	6b1.A/183P1		EPI_ISL_322366
A/Yekaterinburg/472/2018|Europe|04/20/2018	Europe	04/20/2018	6b1.A/183P1		EPI_ISL_331117
A/Baden-Wurttemberg/161/2018|Europe|08/16/2018	Europe	08/16/2018	6b1.A/183P2		EPI_ISL_347331
A/Ireland/07549/2019|Europe|01/08/2019	Europe	01/08/2019	6b1.A/183P3	Global	EPI_ISL_350972
A/Lisboa/35/2018|Europe|12/28/2018	Europe	12/28/2018	6b1.A/183P7	Global	EPI_ISL_338220
A/Serbia/5144/2019|Europe|03/04/2019	Europe	03/04/2019	6b1.A/183P7	Global	EPI_ISL_363045
A/Catalonia/NSVH100959748/2019|Europe|01/06/2019	Europe	01/06/2019	6b1.A/183P7	Global	EPI_ISL_356452
A/Bosnia_and_Herzegovina/72/2018|Europe|12/31/2018	Europe	12/31/2018	6b1.A/183P7	Global	EPI_ISL_338436
A/Mecklenburg-Vorpommern/4/2018|Europe|11/01/2018	Europe	11/01/2018	6b1.A/183P7	Global	EPI_ISL_338075
A/Niedersachsen/160/2019|Europe|04/01/2019	Europe	04/01/2019	6b1.A/183P7	Global	EPI_ISL_355942
A/Uppsala/5/2019|Europe|03/23/2019	Europe	03/23/2019	6b1.A/183P7	Global	EPI_ISL_356853
A/Ireland/22715/2019|Europe|02/26/2019	Europe	02/26/2019	6b1.A/183P7	Global	EPI_ISL_356488
A/Yakutsk/RII-1082S/2019|Europe|01/15/2019	Europe	01/15/2019	6b1.A/183P7	Global	EPI_ISL_337426
A/Lisboa/4/2018|Europe|01/28/2018	Europe	01/28/2018	6b1.A/183P7		EPI_ISL_320000
A/England/595/2018|Europe|10/20/2018	Europe	10/20/2018	6b1.A/183P7	Global	EPI_ISL_338032
A/Neath/9887/2018|Europe|10/23/2018	Europe	10/23/2018	6b1.A/183P7	Global	EPI_ISL_332353
A/Cardiff/6094/2018|Europe|10/30/2018	Europe	10/30/2018	6b1.A/183P7	Global	EPI_ISL_331385
A/Cardiff/6179/2018|Europe|11/01/2018	Europe	11/01/2018	6b1.A/183P7	Global	EPI_ISL_331386
A/Krasnoyarsk/68V/2018|Europe|09/24/2018	Europe	09/24/2018	6b1.A/183P7	Global	EPI_ISL_331621
A/Czech_Republic/319/2018__H1N1_|Europe|02/01/2018	Europe	02/01/2018	6b1.A/183P7		
A/Norway/3188/2018|Europe|06/27/2018	Europe	06/27/2018	6b1.A		EPI_ISL_321776
A/Norway/3226/2018|Europe|07/30/2018	Europe	07/30/2018	6b1.A		EPI_ISL_321782
A/Tomsk/6301/2018|Europe|04/02/2018	Europe	04/02/2018	6b1.A		EPI_ISL_331087

A/Netherlands/10597/2018|Europe|09/13/2018	Europe	09/13/2018	6b1.A	Global	EPI_ISL_333203
A/Stockholm/19/2018|Europe|08/31/2018	Europe	08/31/2018	6b1.A		EPI_ISL_329740
A/Birobidzhan/1219/2018|Europe|05/10/2018	Europe	05/10/2018	6b1.A		EPI_ISL_331116
A/Bosnia_and_Herzegovina/43972/2018|Europe|02/26/2018	Europe	02/26/2018	6b1.A		EPI_ISL_315699
A/Iowa/78/2018|NorthAmerica|12/28/2018	NorthAmerica	12/28/2018	6b1.A/183P5	Global	EPI_ISL_336672
A/Alberta/145/2018|NorthAmerica|11/23/2018	NorthAmerica	11/23/2018	6b1.A/183P5	Global	EPI_ISL_345341
A/Texas/109/2019|NorthAmerica|02/11/2019	NorthAmerica	02/11/2019	6b1.A/183P5	Global	EPI_ISL_348004
A/Illinois/02/2019|NorthAmerica|01/06/2019	NorthAmerica	01/06/2019	6b1.A/183P5	Global	EPI_ISL_339608
A/Virginia/21/2019|NorthAmerica|03/04/2019	NorthAmerica	03/04/2019	6b1.A/183P5	Global	EPI_ISL_354274
A/Arizona/43/2018|NorthAmerica|10/07/2018	NorthAmerica	10/07/2018	6b1.A/183P5	Global	EPI_ISL_331971
A/California/129/2019|NorthAmerica|04/05/2019	NorthAmerica	04/05/2019	6b1.A/183P5	Global	EPI_ISL_356191
A/California/66/2019|NorthAmerica|02/15/2019	NorthAmerica	02/15/2019	6b1.A/183P5	Global	EPI_ISL_348064
A/Trinidad/1967/2019|NorthAmerica|01/23/2019	NorthAmerica	01/23/2019	6b1.A/183P5	Global	EPI_ISL_355504
A/Minnesota/03/2018|NorthAmerica|01/04/2018	NorthAmerica	01/04/2018	6b1.A/183P5		EPI_ISL_296055
A/Alaska/48/2018|NorthAmerica|09/10/2018	NorthAmerica	09/10/2018	6b1.A/183P5	Global	EPI_ISL_330463
A/Michigan/390/2018|NorthAmerica|08/22/2018	NorthAmerica	08/22/2018	6b1.A/183P5		EPI_ISL_329910
A/Quebec/228/2018|NorthAmerica|11/16/2018	NorthAmerica	11/16/2018	6b1.A/183P5	Global	EPI_ISL_345493
A/Michigan/419/2018|NorthAmerica|12/25/2018	NorthAmerica	12/25/2018	6b1.A/183P5	Global	EPI_ISL_338675
A/Iowa/33/2019|NorthAmerica|05/03/2019	NorthAmerica	05/03/2019	6b1.A/183P5	Global	EPI_ISL_357927
A/Iowa/34/2019|NorthAmerica|05/06/2019	NorthAmerica	05/06/2019	6b1.A/183P5	Global	EPI_ISL_357935
A/Illinois/30/2019|NorthAmerica|05/07/2019	NorthAmerica	05/07/2019	6b1.A/183P5	Global	EPI_ISL_357936
A/Maine/16/2019|NorthAmerica|03/12/2019	NorthAmerica	03/12/2019	6b1.A/183P5	Global	EPI_ISL_354353
A/New_York/34/2019|NorthAmerica|04/05/2019	NorthAmerica	04/05/2019	6b1.A/183P5	Global	EPI_ISL_356186
A/New_York/62/2018|NorthAmerica|12/30/2018	NorthAmerica	12/30/2018	6b1.A/183P5	Global	EPI_ISL_348234
A/Ontario/RV2001/2019|NorthAmerica|05/05/2019	NorthAmerica	05/05/2019	6b1.A/183P5	Global	EPI_ISL_362001
A/Maryland/50/2018|NorthAmerica|11/04/2018	NorthAmerica	11/04/2018	6b1.A/183P5	Global	EPI_ISL_335814
A/Idaho/22/2019|NorthAmerica|04/02/2019	NorthAmerica	04/02/2019	6b1.A/183P6	Global	EPI_ISL_354340
A/Kentucky/21/2018|NorthAmerica|03/16/2018	NorthAmerica	03/16/2018	6b1.A/183P6		EPI_ISL_309063
A/Iowa/51/2018|NorthAmerica|04/12/2018	NorthAmerica	04/12/2018	6b1.A/183P6		EPI_ISL_311516
A/Delaware/05/2018|NorthAmerica|01/19/2018	NorthAmerica	01/19/2018	6b1.A/183P6		EPI_ISL_300014
A/Guatemala/387/2018|NorthAmerica|05/15/2018	NorthAmerica	05/15/2018	6b1.A/183P1		EPI_ISL_321264
A/Minnesota/34/2018|NorthAmerica|04/27/2018	NorthAmerica	04/27/2018	6b1.A/183P1		EPI_ISL_331505
A/Alabama/19/2018|NorthAmerica|02/10/2018	NorthAmerica	02/10/2018	6b1.A/183P1		EPI_ISL_302882
A/Nova_Scotia/RV3828/2018|NorthAmerica|04/02/2018	NorthAmerica	04/02/2018	6b1.A/183P1		EPI_ISL_316070

A/Pennsylvania/115/2018|NorthAmerica|03/05/2018	NorthAmerica	03/05/2018	6b1.A/183P1		EPI_ISL_358520
A/Guatemala/091/2018|NorthAmerica|05/24/2018	NorthAmerica	05/24/2018	6b1.A/183P1		EPI_ISL_321262
A/New_York/18/2018|NorthAmerica|02/13/2018	NorthAmerica	02/13/2018	6b1.A/183P1		EPI_ISL_305223
A/Virginia/11/2018|NorthAmerica|02/08/2018	NorthAmerica	02/08/2018	6b1.A/183P1		EPI_ISL_302924
A/Honduras/1973/2018|NorthAmerica|03/19/2018	NorthAmerica	03/19/2018	6b1.A/183P1		EPI_ISL_314796
A/Honduras/1935/2018|NorthAmerica|04/02/2018	NorthAmerica	04/02/2018	6b1.A/183P1		EPI_ISL_314789
A/South_Dakota/43/2018|NorthAmerica|05/07/2018	NorthAmerica	05/07/2018	6b1.A/183P1		EPI_ISL_314027
A/Mexico/2538/2018|NorthAmerica|09/17/2018	NorthAmerica	09/17/2018	6b1.A/183P1	Global	EPI_ISL_336625
A/Alberta/RV3822/2018|NorthAmerica|05/24/2018	NorthAmerica	05/24/2018	6b1.A/183P1		EPI_ISL_332235
A/Saskatchewan/RV1773/2019|NorthAmerica|04/08/2019	NorthAmerica	04/08/2019	6b1.A/183P1	Global	EPI_ISL_354639
A/Honduras/2586/2018|NorthAmerica|06/07/2018	NorthAmerica	06/07/2018	6b1.A/183P1		EPI_ISL_335907
A/El_Salvador/707/2018|NorthAmerica|08/31/2018	NorthAmerica	08/31/2018	6b1.A/183P1		EPI_ISL_333544
A/Florida/60/2018|NorthAmerica|05/07/2018	NorthAmerica	05/07/2018	6b1.A/183P1		EPI_ISL_312932
A/Honduras/2967/2018|NorthAmerica|07/24/2018	NorthAmerica	07/24/2018	6b1.A/183P1		EPI_ISL_336817
A/El_Salvador/454/2018|NorthAmerica|06/24/2018	NorthAmerica	06/24/2018	6b1.A/183P1		EPI_ISL_319747
A/Honduras/2730/2018|NorthAmerica|06/23/2018	NorthAmerica	06/23/2018	6b1.A/183P1		EPI_ISL_336780
A/Honduras/2793/2018|NorthAmerica|06/26/2018	NorthAmerica	06/26/2018	6b1.A/183P1		EPI_ISL_334278
A/El_Salvador/800/2018|NorthAmerica|09/21/2018	NorthAmerica	09/21/2018	6b1.A/183P1	Global	EPI_ISL_335830
A/El_Salvador/654/2018|NorthAmerica|08/26/2018	NorthAmerica	08/26/2018	6b1.A/183P1		EPI_ISL_335947
A/Illinois/23/2018|NorthAmerica|03/20/2018	NorthAmerica	03/20/2018	6b1.A/183P1		EPI_ISL_307718
A/Alberta/189/2018|NorthAmerica|12/03/2018	NorthAmerica	12/03/2018	6b1.A/183P1	Global	EPI_ISL_345367
A/Hawaii/66/2018|NorthAmerica|10/31/2018	NorthAmerica	10/31/2018	6b1.A/183P1	Global	EPI_ISL_335821
A/Minnesota/09/2019|NorthAmerica|02/05/2019	NorthAmerica	02/05/2019	6b1.A/183P1	Global	EPI_ISL_348090
A/Alberta/149/2018|NorthAmerica|11/25/2018	NorthAmerica	11/25/2018	6b1.A/183P1	Global	EPI_ISL_345345
A/Alberta/RV3945/2018|NorthAmerica|09/11/2018	NorthAmerica	09/11/2018	6b1.A/183P1	Global	EPI_ISL_333553
A/Hawaii/21/2019|NorthAmerica|03/22/2019	NorthAmerica	03/22/2019	6b1.A/183P1	Global	EPI_ISL_353391
A/Mexico/2059/2018|NorthAmerica|07/14/2018	NorthAmerica	07/14/2018	6b1.A/183P1		EPI_ISL_332995
A/Yucatan/InDRE2003/2018|NorthAmerica|07/13/2018	NorthAmerica	07/13/2018	6b1.A/183P1		EPI_ISL_322970
A/Mexico/1926/2018|NorthAmerica|07/03/2018	NorthAmerica	07/03/2018	6b1.A/183P1		EPI_ISL_332985
A/British_Columbia/RV4197/2018|NorthAmerica|10/09/2018	NorthAmerica	10/09/2018	6b1.A/183P2	Global	EPI_ISL_333912
A/Tennessee/08/2019|NorthAmerica|01/29/2019	NorthAmerica	01/29/2019	6b1.A/183P2	Global	EPI_ISL_346008
A/Texas/138/2018|NorthAmerica|10/21/2018	NorthAmerica	10/21/2018	6b1.A/183P3	Global	EPI_ISL_331429
A/Louisiana/18/2018|NorthAmerica|08/06/2018	NorthAmerica	08/06/2018	6b1.A/183P3		EPI_ISL_331989
A/Florida/91/2018|NorthAmerica|10/09/2018	NorthAmerica	10/09/2018	6b1.A/183P3	Global	EPI_ISL_332016

A/Colorado/26/2018|NorthAmerica|04/27/2018	NorthAmerica	04/27/2018	6b1.A/183P3		EPI_ISL_312944
A/Vermont/23/2018|NorthAmerica|07/13/2018	NorthAmerica	07/13/2018	6b1.A/183P3		EPI_ISL_320698
A/Washington/129/2018|NorthAmerica|02/05/2018	NorthAmerica	02/05/2018	6b1.A/183P3		EPI_ISL_360851
A/Wisconsin/405/2019|NorthAmerica|05/08/2019	NorthAmerica	05/08/2019	6b1.A/183P7	Global	EPI_ISL_357926
A/Michigan/201/2019|NorthAmerica|03/03/2019	NorthAmerica	03/03/2019	6b1.A/183P7	Global	EPI_ISL_351871
A/West_Virginia/22/2019|NorthAmerica|03/10/2019	NorthAmerica	03/10/2019	6b1.A/183P7	Global	EPI_ISL_354288
A/Indiana/30/2019|NorthAmerica|04/17/2019	NorthAmerica	04/17/2019	6b1.A/183P7	Global	EPI_ISL_357929
A/California/41/2019|NorthAmerica|01/30/2019	NorthAmerica	01/30/2019	6b1.A/183P7	Global	EPI_ISL_344786
A/Utah/16/2019|NorthAmerica|02/06/2019	NorthAmerica	02/06/2019	6b1.A/183P7	Global	EPI_ISL_348246
A/Ohio/35/2018|NorthAmerica|11/19/2018	NorthAmerica	11/19/2018	6b1.A/183P7	Global	EPI_ISL_335808
A/Vermont/28/2018|NorthAmerica|12/23/2018	NorthAmerica	12/23/2018	6b1.A/183P7	Global	EPI_ISL_336800
A/New_York/06/2019|NorthAmerica|01/31/2019	NorthAmerica	01/31/2019	6b1.A	Global	EPI_ISL_341004
A/California/56/2019|NorthAmerica|02/06/2019	NorthAmerica	02/06/2019	6b1.A	Global	EPI_ISL_348062
A/Pennsylvania/127/2018|NorthAmerica|08/15/2018	NorthAmerica	08/15/2018	6b1.A		EPI_ISL_321935
A/Manitoba/RV0444/2018|NorthAmerica|01/10/2018	NorthAmerica	01/10/2018	6b1.A		EPI_ISL_300904
A/Dominican_Republic/514/2018|NorthAmerica|06/13/2018	NorthAmerica	06/13/2018	6b1.A		EPI_ISL_330409
A/Washington/72/2018|NorthAmerica|03/16/2018	NorthAmerica	03/16/2018	6b1.A		EPI_ISL_306792
A/Alberta/RV3930/2018|NorthAmerica|09/01/2018	NorthAmerica	09/01/2018	6b1.A	Global	EPI_ISL_332247
A/Maine/03/2018|NorthAmerica|01/12/2018	NorthAmerica	01/12/2018	6b1.A		EPI_ISL_295919
A/North_Carolina/15/2018|NorthAmerica|02/07/2018	NorthAmerica	02/07/2018	6b1.A		EPI_ISL_304286
A/Wisconsin/239/2018|NorthAmerica|01/26/2018	NorthAmerica	01/26/2018	6b1.A		EPI_ISL_361333
A/Fiji/13/2018|Oceania|03/18/2018	Oceania	03/18/2018	6b1.A/183P5		EPI_ISL_320389
A/Fiji/54/2018|Oceania|05/17/2018	Oceania	05/17/2018	6b1.A/183P5		EPI_ISL_322328
A/Wellington/17/2018|Oceania|07/14/2018	Oceania	07/14/2018	6b1.A/183P5		EPI_ISL_323078
A/New_Caledonia/14/2018|Oceania|04/03/2018	Oceania	04/03/2018	6b1.A/183P5		EPI_ISL_346463
A/New_Caledonia/22/2018|Oceania|05/04/2018	Oceania	05/04/2018	6b1.A/183P5		EPI_ISL_326784
A/New_Caledonia/7/2018|Oceania|02/27/2018	Oceania	02/27/2018	6b1.A/183P5		EPI_ISL_346462
A/New_Caledonia/2/2018|Oceania|02/13/2018	Oceania	02/13/2018	6b1.A/183P5		EPI_ISL_312211
A/New_Caledonia/4/2018|Oceania|02/22/2018	Oceania	02/22/2018	6b1.A/183P5		EPI_ISL_322750
A/New_Caledonia/20/2018|Oceania|04/17/2018	Oceania	04/17/2018	6b1.A/183P5		EPI_ISL_322753
A/Fiji/2/2018|Oceania|01/30/2018	Oceania	01/30/2018	6b1.A/183P5		EPI_ISL_314814
A/Fiji/16/2018|Oceania|03/22/2018	Oceania	03/22/2018	6b1.A/183P6		EPI_ISL_314813
A/Fiji/11/2018|Oceania|03/15/2018	Oceania	03/15/2018	6b1.A/183P6		EPI_ISL_346477
A/Fiji/24/2018|Oceania|04/03/2018	Oceania	04/03/2018	6b1.A/183P6		EPI_ISL_322331

A/Fiji/20/2018|Oceania|02/14/2018	Oceania	02/14/2018	6b1.A/183P6		EPI_ISL_346479
A/Tauranga/5/2018|Oceania|07/18/2018	Oceania	07/18/2018	6b1.A/183P6		EPI_ISL_346467
A/Christchurch/532/2018|Oceania|09/01/2018	Oceania	09/01/2018	6b1.A/183P6	Global	EPI_ISL_332746
A/Canterbury/1/2018|Oceania|04/14/2018	Oceania	04/14/2018	6b1.A/183P6		EPI_ISL_322744
A/Timor-Leste/9/2018|Oceania|02/19/2018	Oceania	02/19/2018	6b1.A/183P6		EPI_ISL_332329
A/Timor-Leste/19/2018|Oceania|03/05/2018	Oceania	03/05/2018	6b1.A/183P6		EPI_ISL_330298
A/Timor-Leste/26/2018|Oceania|03/15/2018	Oceania	03/15/2018	6b1.A/183P6		EPI_ISL_332331
A/Wellington/4/2018|Oceania|06/19/2018	Oceania	06/19/2018	6b1.A/183P6		EPI_ISL_322742
A/Wellington/5/2018|Oceania|06/27/2018	Oceania	06/27/2018	6b1.A/183P6		EPI_ISL_346465
A/Tauranga/9/2018|Oceania|07/16/2018	Oceania	07/16/2018	6b1.A/183P1		EPI_ISL_322746
A/Christchurch/531/2018|Oceania|09/05/2018	Oceania	09/05/2018	6b1.A/183P1	Global	EPI_ISL_346459
A/Christchurch/528/2018|Oceania|09/13/2018	Oceania	09/13/2018	6b1.A/183P1	Global	EPI_ISL_332747
A/North-West_Auckland/1/2018|Oceania|06/20/2018	Oceania	06/20/2018	6b1.A/183P1		EPI_ISL_326786
A/Wellington/3/2018|Oceania|05/24/2018	Oceania	05/24/2018	6b1.A/183P1		EPI_ISL_320393
A/Tauranga/3/2018|Oceania|07/20/2018	Oceania	07/20/2018	6b1.A/183P1		EPI_ISL_322764
A/Christchurch/525/2018|Oceania|09/26/2018	Oceania	09/26/2018	6b1.A/183P2	Global	EPI_ISL_346460
A/Christchurch/506/2018|Oceania|08/14/2018	Oceania	08/14/2018	6b1.A/183P2		EPI_ISL_332493
A/Christchurch/526/2018|Oceania|09/26/2018	Oceania	09/26/2018	6b1.A/183P2	Global	EPI_ISL_332758
A/Christchurch/514/2018|Oceania|08/13/2018	Oceania	08/13/2018	6b1.A/183P2		EPI_ISL_326787
A/Christchurch/515/2018|Oceania|08/15/2018	Oceania	08/15/2018	6b1.A/183P2		EPI_ISL_332494
A/Christchurch/501/2018|Oceania|08/09/2018	Oceania	08/09/2018	6b1.A/183P2		EPI_ISL_330104
A/Wellington/2/2018|Oceania|05/16/2018	Oceania	05/16/2018	6b1.A/183P2		EPI_ISL_320392
A/New_Caledonia/18/2018|Oceania|04/08/2018	Oceania	04/08/2018	6b1.A/183P2		EPI_ISL_322752
A/Papua_New_Guinea/4/2018|Oceania|06/22/2018	Oceania	06/22/2018	6b1.A		EPI_ISL_346474
A/Papua_New_Guinea/5/2018|Oceania|06/22/2018	Oceania	06/22/2018	6b1.A		EPI_ISL_346475
A/Dunedin/3/2018|Oceania|07/10/2018	Oceania	07/10/2018	6b1.A		EPI_ISL_322763
A/Guyane/1881/2019|SouthAmerica|03/03/2019	SouthAmerica	03/03/2019	6b1.A/183P5	Global	EPI_ISL_353967
A/Parana/28/2019|SouthAmerica|01/18/2019	SouthAmerica	01/18/2019	6b1.A/183P5	Global	EPI_ISL_351709
A/Santiago/90003/2018|SouthAmerica|10/24/2018	SouthAmerica	10/24/2018	6b1.A/183P5	Global	EPI_ISL_347494
A/La_Serena/70693/2018|SouthAmerica|08/22/2018	SouthAmerica	08/22/2018	6b1.A/183P5		EPI_ISL_347484
A/Bolivia/171/2018|SouthAmerica|02/28/2018	SouthAmerica	02/28/2018	6b1.A/183P5		EPI_ISL_332206
A/Guyane/1882/2019|SouthAmerica|03/16/2019	SouthAmerica	03/16/2019	6b1.A/183P5	Global	EPI_ISL_353968
A/Santa_Catarina/49/2019|SouthAmerica|02/13/2019	SouthAmerica	02/13/2019	6b1.A/183P5	Global	EPI_ISL_351712
A/Santa_Catarina/50/2019|SouthAmerica|01/30/2019	SouthAmerica	01/30/2019	6b1.A/183P5	Global	EPI_ISL_351713

A/Ecuador/7643/2019|SouthAmerica|01/08/2019	SouthAmerica	01/08/2019	6b1.A/183P5	Global	EPI_ISL_345974
A/Goias/870796/2018|SouthAmerica|02/14/2018	SouthAmerica	02/14/2018	6b1.A/183P5		EPI_ISL_311499
A/Brazil/1438/2018|SouthAmerica|03/14/2018	SouthAmerica	03/14/2018	6b1.A/183P5		EPI_ISL_316746
A/Santiago/58061/2018|SouthAmerica|07/16/2018	SouthAmerica	07/16/2018	6b1.A/183P5		EPI_ISL_321283
A/Espirito_Santo/543/2018|SouthAmerica|05/18/2018	SouthAmerica	05/18/2018	6b1.A/183P5		EPI_ISL_322213
A/Parana/30/2019|SouthAmerica|01/03/2019	SouthAmerica	01/03/2019	6b1.A/183P5	Global	EPI_ISL_351711
A/Parana/142/2018|SouthAmerica|02/21/2018	SouthAmerica	02/21/2018	6b1.A/183P6		EPI_ISL_322246
A/Parana/154/2018|SouthAmerica|03/07/2018	SouthAmerica	03/07/2018	6b1.A/183P6		EPI_ISL_322308
A/Parana/315/2018|SouthAmerica|04/16/2018	SouthAmerica	04/16/2018	6b1.A/183P6		EPI_ISL_322254
A/Bolivia/632/2018|SouthAmerica|04/12/2018	SouthAmerica	04/12/2018	6b1.A/183P6		EPI_ISL_321974
A/Bolivia/1723/2018|SouthAmerica|04/25/2018	SouthAmerica	04/25/2018	6b1.A/183P6		EPI_ISL_322915
A/Bolivia/4069/2018|SouthAmerica|11/23/2018	SouthAmerica	11/23/2018	6b1.A/183P6	Global	EPI_ISL_357920
A/Bolivia/118/2018|SouthAmerica|02/22/2018	SouthAmerica	02/22/2018	6b1.A/183P6		EPI_ISL_333040
A/Minas_Gerais/136/2018|SouthAmerica|03/12/2018	SouthAmerica	03/12/2018	6b1.A/183P6		EPI_ISL_322225
A/Amazonas/156180-IEC/2019|SouthAmerica|02/18/2019	SouthAmerica	02/18/2019	6b1.A/183P1	Global	EPI_ISL_348391
A/Amazonas/156194-IEC/2019|SouthAmerica|02/18/2019	SouthAmerica	02/18/2019	6b1.A/183P1	Global	EPI_ISL_348392
A/Amazonas/156070-IEC/2019|SouthAmerica|02/12/2019	SouthAmerica	02/12/2019	6b1.A/183P1	Global	EPI_ISL_342394
A/Amazonas/156440-IEC/2019|SouthAmerica|03/27/2019	SouthAmerica	03/27/2019	6b1.A/183P1	Global	EPI_ISL_348398
A/Para/156266-IEC/2019|SouthAmerica|02/27/2019	SouthAmerica	02/27/2019	6b1.A/183P1	Global	EPI_ISL_348395
A/Amazonas/156436-IEC/2019|SouthAmerica|03/05/2019	SouthAmerica	03/05/2019	6b1.A/183P1	Global	EPI_ISL_348397
A/Amazonas/156442-IEC/2019|SouthAmerica|03/28/2019	SouthAmerica	03/28/2019	6b1.A/183P1	Global	EPI_ISL_348399
A/Santa_Catarina/1290/2018|SouthAmerica|10/09/2018	SouthAmerica	10/09/2018	6b1.A/183P1	Global	EPI_ISL_345589
A/Santa_Catarina/1302/2018|SouthAmerica|10/24/2018	SouthAmerica	10/24/2018	6b1.A/183P1	Global	EPI_ISL_345590
A/Santa_Catarina/1199/2018|SouthAmerica|09/02/2018	SouthAmerica	09/02/2018	6b1.A/183P1	Global	EPI_ISL_345582
A/Argentina/121/2018|SouthAmerica|07/06/2018	SouthAmerica	07/06/2018	6b1.A/183P1		EPI_ISL_338730
A/Brazil/4335/2018|SouthAmerica|06/20/2018	SouthAmerica	06/20/2018	6b1.A/183P1		EPI_ISL_333064
A/Parana/1309/2018|SouthAmerica|09/20/2018	SouthAmerica	09/20/2018	6b1.A/183P1	Global	EPI_ISL_345591
A/Colombia/6280/2018|SouthAmerica|12/29/2018	SouthAmerica	12/29/2018	6b1.A/183P1	Global	EPI_ISL_340951
A/Colombia/9815/2018|SouthAmerica|12/31/2018	SouthAmerica	12/31/2018	6b1.A/183P1	Global	EPI_ISL_340955
A/Uruguay/682/2018|SouthAmerica|08/09/2018	SouthAmerica	08/09/2018	6b1.A/183P1		EPI_ISL_329991
A/Parana/1080/2018|SouthAmerica|07/18/2018	SouthAmerica	07/18/2018	6b1.A/183P1		EPI_ISL_330437
A/Bolivia/3651/2018|SouthAmerica|06/25/2018	SouthAmerica	06/25/2018	6b1.A/183P1		EPI_ISL_332222
A/Bolivia/3391/2018|SouthAmerica|05/30/2018	SouthAmerica	05/30/2018	6b1.A/183P1		EPI_ISL_333053
A/Bolivia/3327/2018|SouthAmerica|05/28/2018	SouthAmerica	05/28/2018	6b1.A/183P1		EPI_ISL_333050

A/Bolivia/3247/2018|SouthAmerica|05/23/2018	SouthAmerica	05/23/2018	6b1.A/183P1		EPI_ISL_322859
A/Bolivia/4087/2018|SouthAmerica|11/29/2018	SouthAmerica	11/29/2018	6b1.A/183P1	Global	EPI_ISL_357919
A/Colombia/2898/2019|SouthAmerica|01/02/2019	SouthAmerica	01/02/2019	6b1.A/183P2	Global	EPI_ISL_342323
A/Valparaiso/78188/2018|SouthAmerica|09/07/2018	SouthAmerica	09/07/2018	6b1.A/183P3	Global	EPI_ISL_347487
A/Argentina/13808/2018|SouthAmerica|08/15/2018	SouthAmerica	08/15/2018	6b1.A/183P3		EPI_ISL_330212
A/PuertoMontt/6375/2018|SouthAmerica|01/20/2018	SouthAmerica	01/20/2018	6b1.A		EPI_ISL_304370
A/Colombia/2463/2018|SouthAmerica|11/22/2018	SouthAmerica	11/22/2018	6b1.A	Global	EPI_ISL_342324
A/Venezuela/30/2018|SouthAmerica|12/03/2018	SouthAmerica	12/03/2018	6b1.A	Global	EPI_ISL_348094
A/Ecuador/815/2018|SouthAmerica|12/13/2018	SouthAmerica	12/13/2018	6b1.A	Global	EPI_ISL_349808
A/Ecuador/655/2018|SouthAmerica|03/01/2018	SouthAmerica	03/01/2018	6b1.A		EPI_ISL_320243
A/Ecuador/272/2018|SouthAmerica|01/10/2018	SouthAmerica	01/10/2018	6b1.A		EPI_ISL_299994
A/Ecuador/739/2018|SouthAmerica|01/05/2018	SouthAmerica	01/05/2018	6b1.A		EPI_ISL_300079
A/Colombia/2555/2018|SouthAmerica|11/30/2018	SouthAmerica	11/30/2018	6b1.A	Global	EPI_ISL_345989
A/Colombia/0602/2018|SouthAmerica|07/13/2018	SouthAmerica	07/13/2018	6b1.A		EPI_ISL_322905
A/Venezuela/24/2018|SouthAmerica|11/22/2018	SouthAmerica	11/22/2018	6b1.A	Global	EPI_ISL_348093
A/Peru/59818/2018|SouthAmerica|06/06/2018	SouthAmerica	06/06/2018	6b1.A		EPI_ISL_321975
A/Peru/5218/2018|SouthAmerica|05/31/2018	SouthAmerica	05/31/2018	6b1.A		EPI_ISL_319816
A/Colombia/0469/2018|SouthAmerica|12/24/2018	SouthAmerica	12/24/2018	6b1.A	Global	EPI_ISL_345985
A/Bolivia/1912/2018|SouthAmerica|09/05/2018	SouthAmerica	09/05/2018	6b1.A	Global	EPI_ISL_330877
A/Argentina/13806/2018|SouthAmerica|08/15/2018	SouthAmerica	08/15/2018	6b1.A		EPI_ISL_330195
A/Puerto_Montt/87367/2018|SouthAmerica|10/10/2018	SouthAmerica	10/10/2018	6b1.A	Global	EPI_ISL_347106
A/Puerto_Montt/87368/2018|SouthAmerica|10/12/2018	SouthAmerica	10/12/2018	6b1.A	Global	EPI_ISL_347107
A/Osorno/88979/2018|SouthAmerica|09/23/2018	SouthAmerica	09/23/2018	6b1.A	Global	EPI_ISL_347493
A/Peru/45918/2018|SouthAmerica|06/11/2018	SouthAmerica	06/11/2018	6b1.A		EPI_ISL_321972
A/Colombia/8170/2018|SouthAmerica|04/05/2018	SouthAmerica	04/05/2018	6b1.A		EPI_ISL_312918
A/Ecuador/1155/2018|SouthAmerica|06/02/2018	SouthAmerica	06/02/2018	6b1.A		EPI_ISL_320242
A/Venezuela/04/2018|SouthAmerica|08/07/2018	SouthAmerica	08/07/2018	6b1.A		EPI_ISL_321949
A/Colombia/0358/2018|SouthAmerica|07/12/2018	SouthAmerica	07/12/2018	6b1.A		EPI_ISL_322914
A/Ecuador/1755/2018|SouthAmerica|01/02/2018	SouthAmerica	01/02/2018	6b1.A		EPI_ISL_300081
A/Peru/7118/2018|SouthAmerica|03/05/2018	SouthAmerica	03/05/2018	6b1.A		EPI_ISL_319806
A/Ceara/153529-IEC/2018|SouthAmerica|04/16/2018	SouthAmerica	04/16/2018	6b1.A		EPI_ISL_320225
A/Santiago/15715/201|SouthAmerica|02/19/2018	SouthAmerica	02/19/2018	6b1.A		EPI_ISL_314825
A/Guyane/013/2018|SouthAmerica|01/02/2018	SouthAmerica	01/02/2018	6b1.A		EPI_ISL_306110

Supplementary Table 1

A(H3N2) HA sequences used in Figure 4b obtained from GISAID https://www.gisaid.org/

This supplementary material is hosted by *Eurosurveillance* as supporting information alongside the article "Intense interseasonal influenza outbreaks in Australia in 2018-9" on behalf of the authors who remain responsible for the accuracy and appropriateness of the content. The same standards for ethics, copyright, attributions and permissions as for the article apply. *Eurosurveillance* is not responsible for the maintenance of any links or email addresses provided therein

strain	country	date	subtype	location	GISAID
A/Abu_Dhabi/123/2018|Asia|10/13/2018	Asia	10/13/2018	3C.2a1b+131K	Global	EPI_ISL_334105
A/Agadir/757/2018|Africa|01/13/2018	Africa	01/13/2018	3C.2a1b+135K		EPI_ISL_308712
A/Alabama/43/2018|NorthAmerica|12/11/2018	NorthAmerica	12/11/2018	3C.3a	Global	EPI_ISL_338585
A/Alagoas/615/2018|SouthAmerica|05/18/2018	SouthAmerica	05/18/2018	3C.2a2		EPI_ISL_321237
A/Alaska/43/2018|NorthAmerica|05/09/2018	NorthAmerica	05/09/2018	3C.2a2		EPI_ISL_320302
A/Alaska/52/2018|NorthAmerica|09/12/2018	NorthAmerica	09/12/2018	3C.2a2	Global	EPI_ISL_331137
A/Alberta/RV1965/2019|NorthAmerica|04/17/2019	NorthAmerica	04/17/2019	3C.3a	Global	EPI_ISL_358086
A/Alberta/RV3924/2018|NorthAmerica|07/17/2018	NorthAmerica	07/17/2018	3C.2a1b+135N		EPI_ISL_332970
A/Alberta/RV3927/2018|NorthAmerica|08/29/2018	NorthAmerica	08/29/2018	3C.2a3		EPI_ISL_332972
A/Alberta/RV4033/2018|NorthAmerica|10/23/2018	NorthAmerica	10/23/2018	3C.2a1b+135K	Global	EPI_ISL_336760
A/Algeria/27/2019|Africa|02/06/2019	Africa	02/06/2019	3C.2a1b+135K	Global	EPI_ISL_355000
A/Algeria/418/2018|Africa|01/16/2018	Africa	01/16/2018	3C.2a1b+135K		EPI_ISL_308714
A/Algeria/45/2019|Africa|02/14/2019	Africa	02/14/2019	3C.2a1b+135K	Global	EPI_ISL_355002
A/Algeria/55/2019|Africa|02/20/2019	Africa	02/20/2019	3C.2a1b+135K	Global	EPI_ISL_355003
A/Alto_Hospicio/32464/2018|SouthAmerica|03/31/2018	SouthAmerica	03/31/2018	3C.2a2		EPI_ISL_322594
A/Amazonas/151285-IEC/2018|SouthAmerica|02/14/2018	SouthAmerica	02/14/2018	3C.2a2		EPI_ISL_330958
A/Ankara/190/2018|Asia|01/12/2018	Asia	01/12/2018	3C.2a1b+135K		EPI_ISL_312311
A/Ankara/254/2018|Asia|01/18/2018	Asia	01/18/2018	3C.2a1b+135N		EPI_ISL_312315
A/Antofagasta/58996/2018|SouthAmerica|07/10/2018	SouthAmerica	07/10/2018	3C.2a1b+135K		EPI_ISL_330280

A/Antofagasta/77952/2018|SouthAmerica|08/23/2018	SouthAmerica	08/23/2018	3C.2a2		EPI_ISL_347064
A/Ayutthaya/176/2018|Asia|08/26/2018	Asia	08/26/2018	3C.2a2		EPI_ISL_349731
A/Baden-Wurttemberg/27/2019|Europe|02/04/2019	Europe	02/04/2019	3C.2a1b+131K	Global	EPI_ISL_347122
A/Bahia/20/2018|SouthAmerica|01/11/2018	SouthAmerica	01/11/2018	3C.2a3		EPI_ISL_314037
A/Bangladesh/723/2018|Asia|08/27/2018	Asia	08/27/2018	3C.2a1b+135N		EPI_ISL_332958
A/Bayern/49/2018|Europe|10/29/2018	Europe	10/29/2018	3C.2a1b+131K	Global	EPI_ISL_332938
A/Belgium/G0100/2019|Europe|01/28/2019	Europe	01/28/2019	3C.3a	Global	EPI_ISL_354088
A/Belgium/S0039/2019|Europe|12/31/2018	Europe	12/31/2018	3C.2a2	Global	EPI_ISL_355017
A/Belgium/S0043/2018|Europe|01/10/2018	Europe	01/10/2018	3C.2a2		EPI_ISL_298850
A/Belgium/S0515/2019|Europe|01/11/2019	Europe	01/11/2019	3C.2a1b+131K	Global	EPI_ISL_357176
A/Beni_mellal/735/2018|Africa|01/04/2018	Africa	01/04/2018	3C.2a1b+135K		EPI_ISL_308715
A/Bhutan/1219/2018|Asia|09/17/2018	Asia	09/17/2018	3C.2a1b+135K	Global	EPI_ISL_331900
A/Bolivia/0922/2018|SouthAmerica|06/12/2018	SouthAmerica	06/12/2018	3C.2a1b+135K		EPI_ISL_329848
A/Bolivia/150/2018|SouthAmerica|02/27/2018	SouthAmerica	02/27/2018	3C.2a2		EPI_ISL_331069
A/Bolivia/2170/2018|SouthAmerica|10/26/2018	SouthAmerica	10/26/2018	3C.2a2	Global	EPI_ISL_346215
A/Brandenburg/8/2018|Europe|02/07/2018	Europe	02/07/2018	3C.2a2		EPI_ISL_301682
A/Brazil/106/2018|SouthAmerica|04/04/2018	SouthAmerica	04/04/2018	3C.2a2		EPI_ISL_316433
A/Brest/1991/2018|Europe|04/13/2018	Europe	04/13/2018	3C.3a		EPI_ISL_314855
A/Brest/2751/2018|Europe|12/19/2018	Europe	12/19/2018	3C.2a1b+135K	Global	EPI_ISL_335978
A/Brisbane/01/2018|Australia|01/02/2018	Australia	01/02/2018	3C.2a2		EPI_ISL_313657
A/Brisbane/06/2018|Australia|01/01/2018	Australia	01/01/2018	3C.2a2		EPI_ISL_312149
A/Brisbane/07/2018|Australia|01/04/2018	Australia	01/04/2018	3C.2a1b+135K		EPI_ISL_312150
A/Brisbane/08/2018|Australia|01/05/2018	Australia	01/05/2018	3C.2a3		EPI_ISL_312151
A/Brisbane/10/2018|Australia|01/20/2018	Australia	01/20/2018	3C.2a2		EPI_ISL_312152
A/Brisbane/1000/2018|Australia|04/03/2018	Australia	04/03/2018	3C.2a2		EPI_ISL_313809
A/Brisbane/1001/2018|Australia|04/03/2018	Australia	04/03/2018	3C.2a2		EPI_ISL_320416
A/Brisbane/1002/2018|Australia|07/17/2018	Australia	07/17/2018	3C.2a2		EPI_ISL_322336
A/Brisbane/101/2018|Australia|07/02/2018	Australia	07/02/2018	3C.2a1b+135N		EPI_ISL_322714
A/Brisbane/1016/2018|Australia|09/24/2018	Australia	09/24/2018	3C.2a2	Australia	EPI_ISL_333937
A/Brisbane/1028/2018|Australia|12/06/2018	Australia	12/06/2018	3C.2a1b+131K	Australia	EPI_ISL_339233
A/Brisbane/1029/2018|Australia|12/14/2018	Australia	12/14/2018	3C.2a1b+131K	Australia	EPI_ISL_339267
A/Brisbane/104/2018|Australia|07/12/2018	Australia	07/12/2018	3C.2a1b+135N		EPI_ISL_322715
A/Brisbane/106/2018|Australia|07/05/2018	Australia	07/05/2018	3C.2a2		EPI_ISL_322716
A/Brisbane/11/2018|Australia|01/31/2018	Australia	01/31/2018	3C.2a2		EPI_ISL_312153

A/Brisbane/112/2018|Australia|07/25/2018	Australia	07/25/2018	3C.2a2		EPI_ISL_322713
A/Brisbane/115/2018|Australia|07/19/2018	Australia	07/19/2018	3C.2a1b+135K		EPI_ISL_322717
A/Brisbane/117/2018|Australia|07/27/2018	Australia	07/27/2018	3C.2a2		EPI_ISL_322718
A/Brisbane/12/2018|Australia|01/22/2018	Australia	01/22/2018	3C.2a2		EPI_ISL_312154
A/Brisbane/12/2019|Australia|03/04/2019	Australia	03/04/2019	3C.2a1b+131K	Australia	EPI_ISL_354434
A/Brisbane/120/2018|Australia|07/26/2018	Australia	07/26/2018	3C.2a2		EPI_ISL_322726
A/Brisbane/13/2018|Australia|02/03/2018	Australia	02/03/2018			EPI_ISL_312155
A/Brisbane/14/2018|Australia|02/01/2018	Australia	02/01/2018	3C.2a1b+135K		EPI_ISL_312156
A/Brisbane/141/2018|Australia|09/18/2018	Australia	09/18/2018	3C.2a2	Australia	EPI_ISL_339077
A/Brisbane/146/2018|Australia|10/01/2018	Australia	10/01/2018	3C.2a1b+131K	Australia	EPI_ISL_339078
A/Brisbane/154/2018|Australia|10/14/2018	Australia	10/14/2018	3C.2a1b+131K	Australia	EPI_ISL_332641
A/Brisbane/158/2018|Australia|10/19/2018	Australia	10/19/2018	3C.2a3	Australia	EPI_ISL_339079
A/Brisbane/16/2018|Australia|02/05/2018	Australia	02/05/2018	3C.2a2		EPI_ISL_312157
A/Brisbane/161/2018|Australia|10/27/2018	Australia	10/27/2018	3C.2a2	Australia	EPI_ISL_332643
A/Brisbane/168/2018|Australia|11/09/2018	Australia	11/09/2018	3C.2a3	Australia	EPI_ISL_339241
A/Brisbane/17/2018|Australia|02/09/2018	Australia	02/09/2018	3C.2a2		EPI_ISL_312158
A/Brisbane/173/2018|Australia|12/03/2018	Australia	12/03/2018	3C.2a1b+131K	Australia	EPI_ISL_339273
A/Brisbane/176/2018|Australia|12/08/2018	Australia	12/08/2018	3C.2a1b+131K	Australia	EPI_ISL_342177
A/Brisbane/19/2018|Australia|02/13/2018	Australia	02/13/2018	3C.2a2		EPI_ISL_312159
A/Brisbane/22/2018|Australia|02/17/2018	Australia	02/17/2018	3C.2a1b+135K		EPI_ISL_312160
A/Brisbane/23/2018|Australia|02/12/2018	Australia	02/12/2018	3C.2a2		EPI_ISL_312169
A/Brisbane/25/2018|Australia|02/23/2018	Australia	02/23/2018	3C.2a2		EPI_ISL_314628
A/Brisbane/27/2018|Australia|02/20/2018	Australia	02/20/2018	3C.2a2		EPI_ISL_314627
A/Brisbane/28/2018|Australia|02/14/2018	Australia	02/14/2018	3C.2a2		EPI_ISL_314624
A/Brisbane/29/2018|Australia|03/01/2018	Australia	03/01/2018	3C.2a1b+135K		EPI_ISL_314626
A/Brisbane/31/2018|Australia|03/01/2018	Australia	03/01/2018	3C.2a1b+135K		EPI_ISL_314625
A/Brisbane/32/2018|Australia|03/13/2018	Australia	03/13/2018	3C.2a2		EPI_ISL_320942
A/Brisbane/34/2018|Australia|03/17/2018	Australia	03/17/2018	3C.3a		EPI_ISL_330022
A/Brisbane/36/2018|Australia|03/24/2018	Australia	03/24/2018	3C.2a2		EPI_ISL_331870
A/Brisbane/40/2018|Australia|04/04/2018	Australia	04/04/2018	3C.2a2		EPI_ISL_320940
A/Brisbane/42/2018|Australia|03/28/2018	Australia	03/28/2018	3C.2a1b+135K		EPI_ISL_314599
A/Brisbane/43/2018|Australia|04/07/2018	Australia	04/07/2018	3C.2a2		EPI_ISL_330023
A/Brisbane/44/2018|Australia|04/07/2018	Australia	04/07/2018	3C.2a1b+135K		EPI_ISL_314601
A/Brisbane/45/2018|Australia|04/17/2018	Australia	04/17/2018	3C.2a2		EPI_ISL_314602

A/Brisbane/5/2019|Australia|01/10/2019	Australia	01/10/2019	3C.2a2	Australia	EPI_ISL_354431
A/Brisbane/54/2018|Australia|04/26/2018	Australia	04/26/2018	3C.2a2		EPI_ISL_314018
A/Brisbane/55/2018|Australia|04/20/2018	Australia	04/20/2018	3C.2a2		EPI_ISL_314017
A/Brisbane/56/2018|Australia|05/08/2018	Australia	05/08/2018	3C.2a2		EPI_ISL_314016
A/Brisbane/57/2018|Australia|05/09/2018	Australia	05/09/2018	3C.2a2		EPI_ISL_314015
A/Brisbane/58/2018|Australia|04/24/2018	Australia	04/24/2018	3C.2a2		EPI_ISL_320361
A/Brisbane/60/2018|Australia|05/16/2018	Australia	05/16/2018	3C.2a1b+135K		EPI_ISL_320362
A/Brisbane/64/2018|Australia|05/21/2018	Australia	05/21/2018	3C.2a1b+135K		EPI_ISL_320401
A/Brisbane/66/2018|Australia|05/23/2018	Australia	05/23/2018	3C.2a1b+135K		EPI_ISL_320403
A/Brisbane/67/2018|Australia|05/21/2018	Australia	05/21/2018	3C.2a1b+135K		EPI_ISL_320404
A/Brisbane/73/2018|Australia|06/11/2018	Australia	06/11/2018	3C.2a2		EPI_ISL_320405
A/Brisbane/77/2018|Australia|06/12/2018	Australia	06/12/2018	3C.2a2		EPI_ISL_321590
A/Brisbane/85/2018|Australia|07/01/2018	Australia	07/01/2018	3C.2a2		EPI_ISL_321591
A/Brisbane/88/2018|Australia|07/02/2018	Australia	07/02/2018	3C.2a2		EPI_ISL_321454
A/Brisbane/89/2018|Australia|06/21/2018	Australia	06/21/2018	3C.2a2		EPI_ISL_321471
A/Brisbane/94/2018|Australia|07/06/2018	Australia	07/06/2018	3C.2a2		EPI_ISL_321592
A/Bucuresti/239696/2018|Europe|12/23/2018	Europe	12/23/2018	3C.2a1b+135K	Global	EPI_ISL_338743
A/Burkina_Faso/1042/2018|Africa|11/26/2018	Africa	11/26/2018	3C.2a1b+135K	Global	EPI_ISL_362193
A/Burkina_Faso/1045/2018|Africa|11/26/2018	Africa	11/26/2018	3C.2a1b+131K	Global	EPI_ISL_362420
A/Burkina_Faso/1057/2018|Africa|11/30/2018	Africa	11/30/2018	3C.2a3	Global	EPI_ISL_356301
A/Burkina_Faso/1080/2018|Africa|12/12/2018	Africa	12/12/2018	3C.2a1b+135K	Global	EPI_ISL_356298
A/Burkina_Faso/1442/2019|Africa|01/11/2019	Africa	01/11/2019	3C.2a3	Global	EPI_ISL_362553
A/Burkina_Faso/1449/2019|Africa|01/14/2019	Africa	01/14/2019	3C.2a1b+135K	Global	EPI_ISL_356293
A/Burkina_Faso/1464/2019|Africa|01/16/2019	Africa	01/16/2019	3C.2a1b+135K	Global	EPI_ISL_362434
A/Burkina_Faso/2410/2018|Africa|11/18/2018	Africa	11/18/2018	3C.2a1b+135K	Global	EPI_ISL_362192
A/Burkina_Faso/2445/2018|Africa|12/18/2018	Africa	12/18/2018	3C.2a3	Global	EPI_ISL_355604
A/Burkina_Faso/2454/2019|Africa|01/08/2019	Africa	01/08/2019	3C.2a1b+135K	Global	EPI_ISL_362634
A/California/101/2019|NorthAmerica|03/04/2019	NorthAmerica	03/04/2019	3C.2a1b+131K	Global	EPI_ISL_362189
A/California/64/2018|NorthAmerica|10/11/2018	NorthAmerica	10/11/2018	3C.2a2	Global	EPI_ISL_333449
A/California/71/2019|NorthAmerica|02/22/2019	NorthAmerica	02/22/2019	3C.3a	Global	EPI_ISL_355724
A/Cameroon/4372/2018|Africa|04/18/2018	Africa	04/18/2018	3C.2a3		EPI_ISL_330674
A/Cameroon/8607/2018|Africa|10/11/2018	Africa	10/11/2018	3C.2a3	Global	EPI_ISL_358027
A/Cameroon/8932/2018|Africa|10/30/2018	Africa	10/30/2018	3C.2a3	Global	EPI_ISL_341936
A/Cameroon/9008/2018|Africa|10/23/2018	Africa	10/23/2018	3C.2a3	Global	EPI_ISL_358029

A/Cameroon/9319/2018|Africa|10/30/2018	Africa	10/30/2018	3C.2a3	Global	EPI_ISL_341945
A/Cameroon/9696/2018|Africa|11/15/2018	Africa	11/15/2018	3C.2a3	Global	EPI_ISL_341948
A/Canberra/1/2018|Australia|04/25/2018	Australia	04/25/2018	3C.2a2		EPI_ISL_313817
A/Canberra/1000/2018|Australia|02/13/2018	Australia	02/13/2018	3C.2a1b+135N		EPI_ISL_314621
A/Canberra/115/2019|Australia|05/05/2019	Australia	05/05/2019	3C.2a1b+131K	Australia	EPI_ISL_362094
A/Canberra/144/2019|Australia|05/20/2019	Australia	05/20/2019	3C.2a1b+131K	Australia	EPI_ISL_362095
A/Canberra/19/2019|Australia|01/29/2019	Australia	01/29/2019	3C.3a	Australia	EPI_ISL_351025
A/Canberra/23/2019|Australia|02/03/2019	Australia	02/03/2019	3C.3a	Australia	EPI_ISL_351026
A/Canberra/25/2019|Australia|02/05/2019	Australia	02/05/2019	3C.2a1b+135K	Australia	EPI_ISL_354439
A/Canberra/27/2019|Australia|02/08/2019	Australia	02/08/2019	3C.2a1b+131K	Australia	EPI_ISL_354440
A/Canberra/3/2018|Australia|01/27/2018	Australia	01/27/2018	3C.2a2		EPI_ISL_314603
A/Canberra/31/2019|Australia|02/11/2019	Australia	02/11/2019	3C.2a1b+131K	Australia	EPI_ISL_354441
A/Canberra/345/2018|Australia|12/03/2018	Australia	12/03/2018	3C.2a1b+131K	Australia	EPI_ISL_334086
A/Canberra/40/2019|Australia|02/15/2019	Australia	02/15/2019	3C.2a1b+131K	Australia	
A/Canberra/45/2019|Australia|02/15/2019	Australia	02/15/2019	3C.2a1b+131K	Australia	
A/Canberra/52/2019|Australia|02/15/2019	Australia	02/15/2019	3C.2a1b+131K	Australia	
A/Canberra/59/2019|Australia|02/15/2019	Australia	02/15/2019	3C.2a1b+131K	Australia	
A/Canberra/61/2019|Australia|03/17/2019	Australia	03/17/2019	3C.2a1b+131K	Australia	EPI_ISL_356760
A/Canberra/62/2019|Australia|03/24/2019	Australia	03/24/2019	3C.2a1b+131K	Australia	EPI_ISL_356759
A/Canberra/65/2019|Australia|04/06/2019	Australia	04/06/2019	3C.2a1b+131K	Australia	EPI_ISL_356758
A/Canberra/66/2019|Australia|04/07/2019	Australia	04/07/2019	3C.2a1b+131K	Australia	EPI_ISL_356757
A/Canberra/8/2018|Australia|05/26/2018	Australia	05/26/2018	3C.2a2		EPI_ISL_329734
A/Canberra/9/2018|Australia|06/14/2018	Australia	06/14/2018	3C.2a2		EPI_ISL_329735
A/Canterbury/3/2018|Oceania|06/14/2018	Oceania	06/14/2018	3C.2a1b+135N		EPI_ISL_322171
A/Canterbury/4/2018|Oceania|06/12/2018	Oceania	06/12/2018	3C.2a1b+135N		EPI_ISL_322172
A/Canterbury/5/2018|Oceania|04/11/2018	Oceania	04/11/2018	3C.2a1b+135K		EPI_ISL_322173
A/Canterbury/6/2018|Oceania|01/25/2018	Oceania	01/25/2018	3C.2a2		EPI_ISL_322175
A/Canterbury/7/2018|Oceania|01/05/2018	Oceania	01/05/2018	3C.2a2		EPI_ISL_322176
A/Canterbury/8/2018|Oceania|01/05/2018	Oceania	01/05/2018			EPI_ISL_322177
A/Canterbury/8/2019|Oceania|02/15/2019	Oceania	02/15/2019	3C.3a	Global	EPI_ISL_356628
A/Caras_Severin/244476/2019|Europe|03/22/2019	Europe	03/22/2019	3C.2a1b+135K	Global	EPI_ISL_356530
A/Cardiff/8120/2018|Europe|03/07/2018	Europe	03/07/2018	3C.2a2		EPI_ISL_307677
A/Centre/1480/2019|Europe|02/09/2019	Europe	02/09/2019	3C.3a	Global	EPI_ISL_348369
A/Chongqing-Yuzhong/11928/2018|Asia|11/30/2018	Asia	11/30/2018	3C.2a1b+131K	Global	EPI_ISL_334520

A/Christchurch/504/2018|Oceania|08/07/2018	Oceania	08/07/2018	3C.2a2		EPI_ISL_323108
A/Christchurch/505/2018|Oceania|08/15/2018	Oceania	08/15/2018	3C.2a2		EPI_ISL_330735
A/Christchurch/521/2018|Oceania|09/11/2018	Oceania	09/11/2018	3C.2a1b+135N	Global	EPI_ISL_339058
A/Christchurch/522/2018|Oceania|09/25/2018	Oceania	09/25/2018	3C.2a1b+135N	Global	EPI_ISL_332736
A/Colombia/0396/2018|SouthAmerica|12/30/2018	SouthAmerica	12/30/2018	3C.2a1b+135K	Global	EPI_ISL_353522
A/Concepcion/45013/2018|SouthAmerica|05/22/2018	SouthAmerica	05/22/2018	3C.2a2		EPI_ISL_322156
A/Concepcion/48747/2018|SouthAmerica|06/06/2018	SouthAmerica	06/06/2018	3C.2a2		EPI_ISL_322164
A/Congo/003/2019|Africa|01/02/2019	Africa	01/02/2019	3C.2a3	Global	EPI_ISL_347951
A/Congo/297/2018|Africa|05/18/2018	Africa	05/18/2018	3C.2a3		EPI_ISL_320351
A/Congo/326/2018|Africa|05/30/2018	Africa	05/30/2018	3C.2a3		EPI_ISL_320346
A/Congo/357/2018|Africa|06/14/2018	Africa	06/14/2018	3C.2a3		EPI_ISL_320349
A/Congo/416/2018|Africa|07/05/2018	Africa	07/05/2018	3C.2a3		EPI_ISL_329854
A/Congo/422/2018|Africa|07/10/2018	Africa	07/10/2018	3C.2a3		EPI_ISL_329853
A/Congo/726/2018|Africa|12/21/2018	Africa	12/21/2018	3C.2a3	Global	EPI_ISL_347943
A/Congo/730/2018|Africa|12/26/2018	Africa	12/26/2018	3C.2a3	Global	EPI_ISL_348156
A/Congo/737/2018|Africa|12/29/2018	Africa	12/29/2018	3C.2a3	Global	EPI_ISL_349708
A/Connecticut/04/2019|NorthAmerica|01/13/2019	NorthAmerica	01/13/2019	3C.2a2	Global	EPI_ISL_348161
A/Costa_Rica/1932/2018|NorthAmerica|11/27/2018	NorthAmerica	11/27/2018	3C.2a1b+135K	Global	EPI_ISL_346253
A/Dakar/10/2018|Africa|08/03/2018	Africa	08/03/2018	3C.3a		EPI_ISL_347133
A/Dakar/25/2018|Africa|06/12/2018	Africa	06/12/2018	3C.3a		EPI_ISL_347136
A/Dakar/28/2018|Africa|06/12/2018	Africa	06/12/2018	3C.3a		EPI_ISL_347137
A/Dakar/29/2018|Africa|06/12/2018	Africa	06/12/2018	3C.3a		EPI_ISL_347138
A/Darwin/1000/2018|Australia|01/23/2018	Australia	01/23/2018	3C.2a1b+135K		EPI_ISL_314623
A/Darwin/1001/2018|Australia|01/23/2018	Australia	01/23/2018	3C.2a1b+135K		EPI_ISL_314618
A/Darwin/1004/2018|Australia|01/23/2018	Australia	01/23/2018	3C.2a1b+135K		EPI_ISL_314615
A/Darwin/1006/2018|Australia|01/29/2018	Australia	01/29/2018	3C.2a1b+135K		EPI_ISL_314617
A/Darwin/1007/2018|Australia|01/23/2018	Australia	01/23/2018	3C.2a1b+135K		EPI_ISL_313821
A/Darwin/107/2019|Australia|03/23/2019	Australia	03/23/2019	3C.2a1b+131K	Australia	EPI_ISL_356633
A/Darwin/109/2019|Australia|03/26/2019	Australia	03/26/2019	3C.2a1b+131K	Australia	EPI_ISL_354770
A/Darwin/111/2019|Australia|03/27/2019	Australia	03/27/2019	3C.2a1b+131K	Australia	EPI_ISL_356635
A/Darwin/118/2019|Australia|04/03/2019	Australia	04/03/2019	3C.2a1b+131K	Australia	EPI_ISL_356669
A/Darwin/127/2019|Australia|03/25/2019	Australia	03/25/2019	3C.2a1b+131K	Australia	EPI_ISL_356634
A/Darwin/16/2018|Australia|09/27/2018	Australia	09/27/2018	3C.2a2	Australia	EPI_ISL_332655
A/Darwin/3/2019|Australia|01/01/2019	Australia	01/01/2019	3C.2a1b+131K	Australia	EPI_ISL_342169

A/Darwin/339/2019|Australia|05/06/2019	Australia	05/06/2019	3C.2a1b+131K	Australia	EPI_ISL_362091
A/Darwin/35/2019|Australia|01/09/2019	Australia	01/09/2019	3C.2a1b+131K	Australia	EPI_ISL_342170
A/Darwin/362/2019|Australia|05/12/2019	Australia	05/12/2019	3C.2a1b+131K	Australia	EPI_ISL_362093
A/Darwin/394/2019|Australia|05/17/2019	Australia	05/17/2019	3C.2a1b+131K	Australia	EPI_ISL_362097
A/Darwin/45/2018|Australia|11/16/2018	Australia	11/16/2018	3C.2a1b+131K	Australia	EPI_ISL_338273
A/Darwin/52/2019|Australia|01/19/2019	Australia	01/19/2019	3C.2a1b+131K	Australia	EPI_ISL_346297
A/Darwin/6/2019|Australia|01/01/2019	Australia	01/01/2019	3C.2a1b+131K	Australia	EPI_ISL_346298
A/Darwin/61/2019|Australia|01/29/2019	Australia	01/29/2019	3C.2a1b+131K	Australia	EPI_ISL_346300
A/Darwin/70/2019|Australia|02/08/2019	Australia	02/08/2019	3C.2a1b+131K	Australia	EPI_ISL_354501
A/Darwin/94/2019|Australia|03/07/2019	Australia	03/07/2019	3C.2a1b+131K	Australia	EPI_ISL_354443
A/Darwin/99/2019|Australia|03/17/2019	Australia	03/17/2019	3C.2a1b+131K	Australia	EPI_ISL_354430
A/Delaware/01/2019|NorthAmerica|01/04/2019	NorthAmerica	01/04/2019	3C.3a	Global	EPI_ISL_347921
A/Denmark/1578/2019|Europe|02/07/2019	Europe	02/07/2019	3C.2a1b+135K	Global	EPI_ISL_344481
A/Denmark/1829/2018|Europe|11/26/2018	Europe	11/26/2018	3C.2a1b+135K	Global	EPI_ISL_337887
A/Dijon/2017/2019|Europe|04/21/2019	Europe	04/21/2019	3C.2a1b+131K	Global	EPI_ISL_362087
A/Dunedin/1/2018|Oceania|02/24/2018	Oceania	02/24/2018	3C.2a2		EPI_ISL_321456
A/Dunedin/1/2019|Oceania|02/26/2019	Oceania	02/26/2019	3C.2a1b+131K	Global	EPI_ISL_353801
A/Dunedin/12/2019|Oceania|03/28/2019	Oceania	03/28/2019	3C.2a1b+131K	Global	EPI_ISL_356602
A/Dunedin/2/2019|Oceania|02/27/2019	Oceania	02/27/2019	3C.2a1b+131K	Global	EPI_ISL_356610
A/Ecuador/1246/2018|SouthAmerica|11/20/2018	SouthAmerica	11/20/2018	3C.2a1b+135K	Global	EPI_ISL_355741
A/Ecuador/1332/2019|SouthAmerica|01/16/2019	SouthAmerica	01/16/2019	3C.2a1b+135K	Global	EPI_ISL_347927
A/Ecuador/2090/2019|SouthAmerica|01/12/2019	SouthAmerica	01/12/2019	3C.2a1b+135K	Global	EPI_ISL_347932
A/Ecuador/3466/2019|SouthAmerica|01/10/2019	SouthAmerica	01/10/2019	3C.2a1b+135K	Global	EPI_ISL_347933
A/Ecuador/3480/2019|SouthAmerica|01/15/2019	SouthAmerica	01/15/2019	3C.2a1b+135K	Global	EPI_ISL_357824
A/Ecuador/4107/2018|SouthAmerica|12/27/2018	SouthAmerica	12/27/2018	3C.2a1b+135K	Global	EPI_ISL_355735
A/Ecuador/4125/2019|SouthAmerica|01/03/2019	SouthAmerica	01/03/2019	3C.2a1b+135K	Global	EPI_ISL_355736
A/Egypt/5730/2018|Africa|09/20/2018	Africa	09/20/2018	3C.2a1b+135K	Global	EPI_ISL_336077
A/Egypt/5816/2018|Africa|09/28/2018	Africa	09/28/2018	3C.2a1b+135K	Global	EPI_ISL_336078
A/EHIME/27/2018|Asia|03/09/2018	Asia	03/09/2018	3C.2a2		EPI_ISL_314322
A/EHIME/35/2018|Asia|09/19/2018	Asia	09/19/2018	3C.2a1b+135K	Global	EPI_ISL_333927
A/England/80220576/2018|Europe|01/04/2018	Europe	01/04/2018	3C.2a1b+135K		EPI_ISL_293737
A/England/80340457/2018|Europe|01/14/2018	Europe	01/14/2018	3C.2a2		EPI_ISL_298105
A/England/80400415/2018|Europe|01/18/2018	Europe	01/18/2018	3C.2a1b+135K		EPI_ISL_303455
A/England/80600014/2018|Europe|02/01/2018	Europe	02/01/2018	3C.2a2		EPI_ISL_303394

A/England/80680583/2018|Europe|02/06/2018	Europe	02/06/2018	3C.2a2		EPI_ISL_303405
A/England/81120500/2018|Europe|03/06/2018	Europe	03/06/2018	3C.2a2		EPI_ISL_312067
A/Estonia/113528/2018|Europe|04/02/2018	Europe	04/02/2018	3C.2a2		EPI_ISL_320615
A/Fiji/37/2018|Oceania|04/09/2018	Oceania	04/09/2018	3C.2a1b+135N		EPI_ISL_320374
A/Fiji/38/2018|Oceania|04/05/2018	Oceania	04/05/2018	3C.2a1b+135N		EPI_ISL_320375
A/Fiji/48/2018|Oceania|04/20/2018	Oceania	04/20/2018	3C.2a1b+135N		EPI_ISL_320376
A/Fiji/49/2018|Oceania|04/26/2018	Oceania	04/26/2018	3C.2a1b+135N		EPI_ISL_320363
A/Fiji/56/2018|Oceania|05/18/2018	Oceania	05/18/2018	3C.2a1b+135N		EPI_ISL_320369
A/Fiji/61/2018|Oceania|06/06/2018	Oceania	06/06/2018	3C.2a1b+135N		EPI_ISL_340089
A/Fiji/68/2018|Oceania|06/19/2018	Oceania	06/19/2018	3C.2a1b+135N		EPI_ISL_340090
A/Fiji/71/2018|Oceania|10/24/2018	Oceania	10/24/2018	3C.2a2	Global	EPI_ISL_339248
A/Fiji/8/2018|Oceania|03/13/2018	Oceania	03/13/2018	3C.2a1b+135N		EPI_ISL_320372
A/Fiji/9/2018|Oceania|02/27/2018	Oceania	02/27/2018	3C.2a3		EPI_ISL_320365
A/Finland/106/2019|Europe|04/15/2019	Europe	04/15/2019	3C.2a1b+131K	Global	EPI_ISL_357770
A/Finland/123/2019|Europe|05/08/2019	Europe	05/08/2019	3C.2a1b+131K	Global	EPI_ISL_357745
A/Florida/109/2018|NorthAmerica|11/15/2018	NorthAmerica	11/15/2018	3C.3a	Global	EPI_ISL_336529
A/Georgia/35/2018|NorthAmerica|12/27/2018	NorthAmerica	12/27/2018	3C.3a	Global	EPI_ISL_349872
A/Georgia/725/2019|Asia|04/01/2019	Asia	04/01/2019	3C.2a1b+135K	Global	EPI_ISL_361945
A/Greece/1456/19/2019|Europe|03/15/2019	Europe	03/15/2019	3C.2a1b+135K	Global	EPI_ISL_355042
A/Guatemala/126/2018|NorthAmerica|06/26/2018	NorthAmerica	06/26/2018	3C.2a2		EPI_ISL_330251
A/Guatemala/144/2018|NorthAmerica|05/13/2018	NorthAmerica	05/13/2018	3C.2a2		EPI_ISL_321215
A/Guatemala/183/2018|NorthAmerica|06/17/2018	NorthAmerica	06/17/2018	3C.2a2		EPI_ISL_330252
A/Guyane/023/2018|SouthAmerica|01/03/2018	SouthAmerica	01/03/2018	3C.2a2		EPI_ISL_299897
A/Guyane/1877/2019|SouthAmerica|02/01/2019	SouthAmerica	02/01/2019	3C.3a	Global	EPI_ISL_353964
A/Guyane/1879/2019|SouthAmerica|02/26/2019	SouthAmerica	02/26/2019	3C.3a	Global	EPI_ISL_353966
A/Guyane/1886/2019|SouthAmerica|02/26/2019	SouthAmerica	02/26/2019	3C.3a	Global	EPI_ISL_353970
A/Guyane/1888/2019|SouthAmerica|02/27/2019	SouthAmerica	02/27/2019	3C.2a1b+135K	Global	EPI_ISL_353971
A/Guyane/1894/2019|SouthAmerica|03/05/2019	SouthAmerica	03/05/2019	3C.2a1b+135K	Global	EPI_ISL_353974
A/Guyane/1895/2019|SouthAmerica|03/12/2019	SouthAmerica	03/12/2019	3C.2a2	Global	EPI_ISL_353975
A/Haute_Normandie/2695/2018|Europe|10/26/2018	Europe	10/26/2018	3C.2a2	Global	EPI_ISL_335967
A/Hawaii/49/2018|NorthAmerica|07/26/2018	NorthAmerica	07/26/2018	3C.2a2		EPI_ISL_330018
A/Hawaii/50/2018|NorthAmerica|08/01/2018	NorthAmerica	08/01/2018	3C.2a1b+135N		EPI_ISL_330019
A/Hawkes_Bay/1/2019|Africa|03/03/2019	Africa	03/03/2019	3C.2a1b+131K	Global	EPI_ISL_353800
A/Hawkes_Bay/1/2019|Oceania|03/03/2019	Oceania	03/03/2019	3C.2a1b+131K	Global	EPI_ISL_353800

A/Hawkes_Bay/3/2019|Africa|03/08/2019	Africa	03/08/2019	3C.2a1b+131K	Global	EPI_ISL_356624
A/Heilongjiang-Aihui/1208/2018|Asia|03/09/2018	Asia	03/09/2018	3C.2a2		EPI_ISL_334996
A/Henan-Beiguan/524/2018|Asia|12/26/2018	Asia	12/26/2018	3C.2a2	Global	EPI_ISL_336872
A/Hessen/56/2019|Europe|03/20/2019	Europe	03/20/2019	3C.2a1b+135K	Global	EPI_ISL_358039
A/HIROSHIMA-C/9/2018|Asia|04/09/2018	Asia	04/09/2018	3C.2a2		EPI_ISL_320563
A/HK/1078/2018|Asia|06/08/2018	Asia	06/08/2018	3C.2a1b+135K		EPI_ISL_319862
A/HK/1100/2018|Asia|06/12/2018	Asia	06/12/2018	3C.2a1b+135K		EPI_ISL_319863
A/HK/1162/2018|Asia|06/28/2018	Asia	06/28/2018	3C.2a2		EPI_ISL_319865
A/HK/121/2019|Asia|01/02/2019	Asia	01/02/2019	3C.2a1b+135K	Global	EPI_ISL_350950
A/HK/2693/2018|Asia|11/12/2018	Asia	11/12/2018	3C.2a1b+135N	Global	EPI_ISL_337490
A/HK/38/2019|Asia|12/23/2018	Asia	12/23/2018	3C.2a1b+131K	Global	EPI_ISL_337499
A/Hong_Kong/1100/2018|Asia|06/12/2018	Asia	06/12/2018	3C.2a1b+135K		EPI_ISL_320631
A/Hong_Kong/2060/2018|Asia|09/21/2018	Asia	09/21/2018	3C.2a1b+135N	Global	EPI_ISL_349720
A/Hong_Kong/639/2018|Asia|04/01/2018	Asia	04/01/2018	3C.2a1b+135K		EPI_ISL_312264
A/Hubei-Jiangan/1140/2019|Asia|02/11/2019	Asia	02/11/2019	3C.2a1b+131K	Global	EPI_ISL_344266
A/Iceland/88/2018|Europe|05/09/2018	Europe	05/09/2018	3C.2a1b+135K		EPI_ISL_315973
A/Iceland/98/2018|Europe|10/11/2018	Europe	10/11/2018	3C.2a1b+135K	Global	EPI_ISL_337898
A/Indiana/33/2019|NorthAmerica|05/02/2019	NorthAmerica	05/02/2019	3C.3a	Global	EPI_ISL_357879
A/Iowa/83/2018|NorthAmerica|12/27/2018	NorthAmerica	12/27/2018	3C.3a	Global	EPI_ISL_348162
A/Iquique/21652/2019|SouthAmerica|02/21/2019	SouthAmerica	02/21/2019	3C.2a2	Global	EPI_ISL_355849
A/Iquique/44550/2018|SouthAmerica|05/14/2018	SouthAmerica	05/14/2018	3C.2a2		EPI_ISL_322151
A/Iquique/50626/2018|SouthAmerica|06/11/2018	SouthAmerica	06/11/2018	3C.2a2		EPI_ISL_355794
A/Iquique/73982/2018|SouthAmerica|08/21/2018	SouthAmerica	08/21/2018	3C.2a2		EPI_ISL_347050
A/Iquique/90057/2018|SouthAmerica|09/18/2018	SouthAmerica	09/18/2018	3C.2a2	Global	EPI_ISL_347089
A/Iran/168336/2019|Asia|03/07/2019	Asia	03/07/2019	3C.2a1b+131K	Global	EPI_ISL_361872
A/Iran/171909/2019|Asia|04/12/2019	Asia	04/12/2019	3C.2a1b+131K	Global	EPI_ISL_361875
A/Ireland/77960/2018|Europe|11/05/2018	Europe	11/05/2018	3C.2a1b+131K	Global	EPI_ISL_337902
A/Jamaica/7492/2018|NorthAmerica|04/25/2018	NorthAmerica	04/25/2018	3C.2a2		EPI_ISL_322941
A/Jilin-Longshan/1235/2018|Asia|03/14/2018	Asia	03/14/2018	3C.2a1b+135K		EPI_ISL_334992
A/KANAGAWA/40/2018|Asia|01/30/2018	Asia	01/30/2018	3C.2a2		EPI_ISL_316100
A/KANAGAWA/68/2018|Asia|03/06/2018	Asia	03/06/2018	3C.2a2		EPI_ISL_316101
A/KANAGAWA/AC1729/2018|Asia|02/21/2018	Asia	02/21/2018	3C.2a2		EPI_ISL_311968
A/KANAGAWA/AC1873/2019|Asia|02/24/2019	Asia	02/24/2019	3C.2a1b+131K	Global	EPI_ISL_356874
A/KANAGAWA/AC1878/2019|Asia|03/11/2019	Asia	03/11/2019	3C.2a1b+131K	Global	EPI_ISL_356752

A/KANAGAWA/IC1807/2018|Asia|12/20/2018	Asia	12/20/2018	3C.2a1b+131K	Global	EPI_ISL_340686
A/KANAGAWA/IC18154/2019|Asia|02/16/2019	Asia	02/16/2019	3C.2a1b+131K	Global	EPI_ISL_345223
A/KANAGAWA/IC18160/2019|Asia|02/21/2019	Asia	02/21/2019	3C.2a1b+131K	Global	EPI_ISL_344510
A/KANAGAWA/ZC1843/2019|Asia|02/06/2019	Asia	02/06/2019	3C.2a1b+131K	Global	EPI_ISL_344520
A/KANAGAWA/ZC1858/2019|Asia|03/07/2019	Asia	03/07/2019	3C.2a1b+131K	Global	EPI_ISL_356878
A/Kansas/09/2019|NorthAmerica|02/11/2019	NorthAmerica	02/11/2019	3C.3a	Global	EPI_ISL_351914
A/Kazakhstan/129/2018|Asia|02/01/2018	Asia	02/01/2018	3C.2a2		EPI_ISL_306253
A/Kazakhstan/A-09/2018|Asia|04/04/2018	Asia	04/04/2018	3C.2a2		EPI_ISL_321855
A/Kentucky/23/2018|NorthAmerica|03/27/2018	NorthAmerica	03/27/2018	3C.2a2		EPI_ISL_308983
A/Kenya/154/2018|Africa|07/16/2018	Africa	07/16/2018	3C.3a		EPI_ISL_346059
A/Kenya/155/2018|Africa|07/17/2018	Africa	07/17/2018	3C.3a		EPI_ISL_346061
A/Kenya/159/2018|Africa|08/01/2018	Africa	08/01/2018	3C.3a		EPI_ISL_346060
A/Kenya/160/2018|Africa|08/01/2018	Africa	08/01/2018	3C.3a		EPI_ISL_338561
A/Kenya/163/2018|Africa|07/31/2018	Africa	07/31/2018	3C.2a1b		EPI_ISL_346063
A/Kenya/166/2018|Africa|06/05/2018	Africa	06/05/2018	3C.2a1b		EPI_ISL_335649
A/Khanti-Mansiysk/4/2018|Europe|11/23/2018	Europe	11/23/2018	3C.2a1b+135K	Global	EPI_ISL_339098
A/KOCHI/9/2018|Asia|01/15/2018	Asia	01/15/2018	3C.2a3		EPI_ISL_316099
A/Kuwait/6355/2018|Asia|06/11/2018	Asia	06/11/2018	3C.2a1b+135K		EPI_ISL_346083
A/Kuwait/6430/2018|Asia|07/11/2018	Asia	07/11/2018	3C.2a1b+135K		EPI_ISL_346091
A/Kyiv/500/2018|Europe|12/14/2018	Europe	12/14/2018	3C.2a1b+135K	Global	EPI_ISL_346120
A/La_Reunion/1972/2018|Europe|09/19/2018	Europe	09/19/2018	3C.2a1b+135K	Global	EPI_ISL_337909
A/Lebanon/234/2018|Asia|02/13/2018	Asia	02/13/2018	3C.2a1b+135K		EPI_ISL_314379
A/Lebanon/679/2018|Asia|03/17/2018	Asia	03/17/2018	3C.2a1b+135K		EPI_ISL_346075
A/Lipetsk/129/2018|Europe|03/28/2018	Europe	03/28/2018	3C.2a1b+135K		EPI_ISL_322481
A/Lipetsk/160/2018|Europe|04/06/2018	Europe	04/06/2018	3C.2a1b+135K		EPI_ISL_320638
A/Llansamlet/9638/2019|Europe|04/15/2019	Europe	04/15/2019	3C.2a1b+131K	Global	EPI_ISL_356500
A/Luxembourg/19005983/2019|Europe|01/31/2019	Europe	01/31/2019	3C.2a1b+131K	Global	EPI_ISL_345549
A/Lyon/CHU/1520846/2018|Europe|09/02/2018	Europe	09/02/2018	3C.2a1b+135K	Global	EPI_ISL_345740
A/Madagascar/1123/2018|Africa|03/26/2018	Africa	03/26/2018	3C.2a1b+135N		EPI_ISL_330970
A/Madagascar/424/2018|Africa|02/06/2018	Africa	02/06/2018	3C.2a1b+135K		EPI_ISL_331071
A/Maine/41/2018|NorthAmerica|12/15/2018	NorthAmerica	12/15/2018	3C.2a1b+135K	Global	EPI_ISL_338716
A/Malaysia/RP3325/2018|Asia|09/08/2018	Asia	09/08/2018	3C.2a1b+131K	Global	EPI_ISL_339258
A/Maldives/340/2018|Asia|05/12/2018	Asia	05/12/2018	3C.2a1b+131K		EPI_ISL_330948
A/Mali/057/2018|Africa|10/08/2018	Africa	10/08/2018	3C.2a3	Global	EPI_ISL_353702

A/Malta/32668/2018|Europe|01/05/2018	Europe	01/05/2018	3C.2a1b+135K		EPI_ISL_309297
A/Manitoba/RV1999/2019|NorthAmerica|05/14/2019	NorthAmerica	05/14/2019	3C.3a	Global	EPI_ISL_361999
A/Manitoba/RV2025/2019|NorthAmerica|05/01/2019	NorthAmerica	05/01/2019	3C.3a	Global	EPI_ISL_362015
A/Manitoba/RV3992/2018|NorthAmerica|10/26/2018	NorthAmerica	10/26/2018	3C.2a1b+135K	Global	EPI_ISL_341060
A/Maryland/19/2018|NorthAmerica|02/05/2018	NorthAmerica	02/05/2018	3C.2a2		EPI_ISL_308951
A/Mauritius/2263/2018|Africa|05/02/2018	Africa	05/02/2018	3C.2a2		EPI_ISL_321447
A/Mauritius/2287/2018|Africa|05/10/2018	Africa	05/10/2018	3C.2a1b+135N		EPI_ISL_320642
A/Mauritius/I-851/2018|Africa|09/16/2018	Africa	09/16/2018	3C.2a1b+135K	Global	EPI_ISL_332808
A/Mauritius/I-888/2018|Africa|09/28/2018	Africa	09/28/2018	3C.2a2	Global	EPI_ISL_332809
A/Meknes/1218/2018|Africa|02/13/2018	Africa	02/13/2018	3C.2a1b+135K		EPI_ISL_314175
A/Michigan/245/2018|NorthAmerica|01/30/2018	NorthAmerica	01/30/2018	3C.2a2		EPI_ISL_360638
A/Michigan/282/2019|NorthAmerica|04/02/2019	NorthAmerica	04/02/2019	3C.3a	Global	EPI_ISL_357844
A/Michigan/360/2018|NorthAmerica|03/10/2018	NorthAmerica	03/10/2018	3C.2a2		EPI_ISL_360517
A/Michigan/401/2018|NorthAmerica|09/30/2018	NorthAmerica	09/30/2018	3C.2a1b+135K	Global	EPI_ISL_331131
A/Michigan/74/2018|NorthAmerica|04/04/2018	NorthAmerica	04/04/2018	3C.3a		EPI_ISL_309558
A/Michigan/90/2018|NorthAmerica|01/04/2018	NorthAmerica	01/04/2018	3C.2a2		EPI_ISL_360562
A/Minas_Gerais/39/2018|SouthAmerica|01/13/2018	SouthAmerica	01/13/2018	3C.2a2		EPI_ISL_314036
A/Minnesota/54/2018|NorthAmerica|10/26/2018	NorthAmerica	10/26/2018	3C.2a1b+131K	Global	EPI_ISL_336745
A/Montana/26/2019|NorthAmerica|03/21/2019	NorthAmerica	03/21/2019	3C.3a	Global	EPI_ISL_362413
A/Moscow/185/2018|Europe|05/01/2018	Europe	05/01/2018	3C.2a1b+135K		EPI_ISL_329836
A/Moscow/219/2018|Europe|12/20/2018	Europe	12/20/2018	3C.2a1b+135K	Global	EPI_ISL_342006
A/Mozambique/0418/2018|Africa|02/14/2018	Africa	02/14/2018	3C.2a1b		EPI_ISL_320718
A/Mozambique/2118/2018|Africa|02/16/2018	Africa	02/16/2018	3C.2a1b		EPI_ISL_320808
A/Mugla/8/2018|Asia|12/24/2018	Asia	12/24/2018	3C.2a1b+135K	Global	EPI_ISL_337169
A/Myanmar/18M269/2018|Asia|10/03/2018	Asia	10/03/2018	3C.2a1b+135K	Global	EPI_ISL_348312
A/Nebraska/08/2018|NorthAmerica|07/20/2018	NorthAmerica	07/20/2018	3C.2a2		EPI_ISL_330491
A/Netherlands/00010/2019|Europe|01/02/2019	Europe	01/02/2019	3C.2a1b+135K	Global	EPI_ISL_336174
A/Netherlands/00563/2019|Europe|02/21/2019	Europe	02/21/2019	3C.2a1b+135K	Global	EPI_ISL_345716
A/Netherlands/00627/2019|Europe|02/20/2019	Europe	02/20/2019	3C.3a	Global	EPI_ISL_347546
A/Netherlands/10240/2019|Europe|03/28/2019	Europe	03/28/2019	3C.2a1b+135K	Global	EPI_ISL_350668
A/Netherlands/10245/2019|Europe|04/05/2019	Europe	04/05/2019	3C.3a	Global	EPI_ISL_351174
A/Netherlands/10260/2018|Europe|02/15/2018	Europe	02/15/2018	3C.2a1b+135K		EPI_ISL_348116
A/Netherlands/10541/2018|Europe|03/29/2018	Europe	03/29/2018	3C.2a2		EPI_ISL_306988
A/Netherlands/10601/2018|Europe|10/16/2018	Europe	10/16/2018	3C.2a1b+135K	Global	EPI_ISL_342010

A/Netherlands/10606/2018|Europe|11/30/2018	Europe	11/30/2018	3C.2a1b+135K	Global	EPI_ISL_342011
A/New_Brunswick/RV3876/2018|NorthAmerica|05/21/2018	NorthAmerica	05/21/2018	3C.2a2		EPI_ISL_316499
A/New_Brunswick/RV3888/2018|NorthAmerica|05/24/2018	NorthAmerica	05/24/2018	3C.2a2		EPI_ISL_332967
A/New_Caledonia/1/2018|Oceania|01/25/2018	Oceania	01/25/2018	3C.2a2		EPI_ISL_312145
A/New_Caledonia/1/2019|Oceania|01/09/2019	Oceania	01/09/2019	3C.2a1b+131K	Global	EPI_ISL_356593
A/New_Caledonia/23/2018|Oceania|06/03/2018	Oceania	06/03/2018	3C.2a1b+135N		EPI_ISL_329730
A/New_Caledonia/8/2018|Oceania|02/28/2018	Oceania	02/28/2018	3C.2a1b+135K		EPI_ISL_322728
A/New_Jersey/35/2018|NorthAmerica|05/14/2018	NorthAmerica	05/14/2018	3C.2a2		EPI_ISL_314349
A/New_Jersey/38/2018|NorthAmerica|07/10/2018	NorthAmerica	07/10/2018	3C.2a2		EPI_ISL_320740
A/New_Jersey/41/2018|NorthAmerica|08/11/2018	NorthAmerica	08/11/2018	3C.2a1b+135N		EPI_ISL_329794
A/New_Mexico/10/2019|NorthAmerica|02/10/2019	NorthAmerica	02/10/2019	3C.2a1b+131K	Global	EPI_ISL_353623
A/Newcastle/10/2019|Australia|01/12/2019	Australia	01/12/2019	3C.2a1b+131K	Australia	EPI_ISL_346331
A/Newcastle/1001/2018|Australia|09/05/2018	Australia	09/05/2018	3C.2a1b+131K	Australia	EPI_ISL_330113
A/Newcastle/104/2018|Australia|12/06/2018	Australia	12/06/2018	3C.2a1b+131K	Australia	EPI_ISL_339347
A/Newcastle/105/2018|Australia|12/15/2018	Australia	12/15/2018	3C.2a1b+131K	Australia	EPI_ISL_335439
A/Newcastle/106/2018|Australia|12/14/2018	Australia	12/14/2018	3C.2a1b+135K	Australia	EPI_ISL_339246
A/Newcastle/11/2018|Australia|03/23/2018	Australia	03/23/2018	3C.2a2		EPI_ISL_320378
A/Newcastle/11/2019|Australia|01/15/2019	Australia	01/15/2019	3C.2a1b+135K	Australia	EPI_ISL_346332
A/Newcastle/113/2018|Australia|11/06/2018	Australia	11/06/2018	3C.2a2	Australia	EPI_ISL_339247
A/Newcastle/12/2018|Australia|02/22/2018	Australia	02/22/2018	3C.2a2		EPI_ISL_320379
A/Newcastle/12/2019|Australia|01/02/2019	Australia	01/02/2019	3C.2a1b+131K	Australia	EPI_ISL_346303
A/Newcastle/13/2018|Australia|03/13/2018	Australia	03/13/2018	3C.2a2		EPI_ISL_320380
A/Newcastle/13/2019|Australia|01/06/2019	Australia	01/06/2019	3C.2a2	Australia	EPI_ISL_346324
A/Newcastle/14/2019|Australia|01/12/2019	Australia	01/12/2019	3C.2a1b+131K	Australia	EPI_ISL_346304
A/Newcastle/145/2018|Australia|12/18/2018	Australia	12/18/2018	3C.2a2	Australia	EPI_ISL_339236
A/Newcastle/146/2018|Australia|12/13/2018	Australia	12/13/2018	3C.2a2	Australia	EPI_ISL_339243
A/Newcastle/148/2018|Australia|12/18/2018	Australia	12/18/2018	3C.2a1b+131K	Australia	EPI_ISL_342176
A/Newcastle/15/2018|Australia|04/22/2018	Australia	04/22/2018	3C.2a1b+135K		EPI_ISL_320381
A/Newcastle/155/2018|Australia|12/05/2018	Australia	12/05/2018	3C.2a1b+131K	Australia	EPI_ISL_339278
A/Newcastle/156/2018|Australia|12/05/2018	Australia	12/05/2018	3C.2a1b+131K	Australia	EPI_ISL_339279
A/Newcastle/158/2018|Australia|12/03/2018	Australia	12/03/2018	3C.2a1b+131K	Australia	EPI_ISL_339266
A/Newcastle/16/2018|Australia|04/22/2018	Australia	04/22/2018	3C.2a3		EPI_ISL_320371
A/Newcastle/161/2018|Australia|12/05/2018	Australia	12/05/2018	3C.2a2	Australia	EPI_ISL_339232
A/Newcastle/17/2018|Australia|03/01/2018	Australia	03/01/2018	3C.2a1b+135N		EPI_ISL_320382

A/Newcastle/18/2019|Australia|03/07/2019	Australia	03/07/2019	3C.2a1b+131K	Australia	EPI_ISL_354740
A/Newcastle/19/2018|Australia|03/12/2018	Australia	03/12/2018	3C.2a1b+135K		EPI_ISL_329733
A/Newcastle/19/2019|Australia|03/09/2019	Australia	03/09/2019	3C.2a1b+131K	Australia	EPI_ISL_354721
A/Newcastle/2/2019|Australia|01/21/2019	Australia	01/21/2019	3C.2a1b+131K	Australia	EPI_ISL_339080
A/Newcastle/21/2018|Australia|08/08/2018	Australia	08/08/2018	3C.2a2		EPI_ISL_329731
A/Newcastle/21/2019|Australia|03/07/2019	Australia	03/07/2019	3C.2a1b+131K	Australia	EPI_ISL_354722
A/Newcastle/24/2019|Australia|03/11/2019	Australia	03/11/2019	3C.2a1b+131K	Australia	EPI_ISL_354741
A/Newcastle/25/2019|Australia|03/10/2019	Australia	03/10/2019	3C.2a1b+131K	Australia	EPI_ISL_354742
A/Newcastle/3/2019|Australia|01/10/2019	Australia	01/10/2019	3C.2a1b+131K	Australia	EPI_ISL_339082
A/Newcastle/30/2019|Australia|03/11/2019	Australia	03/11/2019	3C.2a1b+131K	Australia	EPI_ISL_354723
A/Newcastle/32/2019|Australia|03/11/2019	Australia	03/11/2019	3C.2a1b+131K	Australia	EPI_ISL_354743
A/Newcastle/33/2019|Australia|03/11/2019	Australia	03/11/2019	3C.2a1b+131K	Australia	EPI_ISL_354724
A/Newcastle/35/2019|Australia|03/12/2019	Australia	03/12/2019	3C.2a1b+131K	Australia	EPI_ISL_354725
A/Newcastle/36/2019|Australia|03/19/2019	Australia	03/19/2019	3C.2a1b+131K	Australia	EPI_ISL_354726
A/Newcastle/39/2018|Australia|07/26/2018	Australia	07/26/2018	3C.2a1b+135N		EPI_ISL_329732
A/Newcastle/39/2019|Australia|03/17/2019	Australia	03/17/2019	3C.2a1b+131K	Australia	EPI_ISL_354727
A/Newcastle/4/2018|Australia|01/01/2018	Australia	01/01/2018	3C.2a1b+135K		EPI_ISL_312147
A/Newcastle/4/2019|Australia|01/17/2019	Australia	01/17/2019	3C.2a1b+131K	Australia	EPI_ISL_346327
A/Newcastle/40/2019|Australia|03/18/2019	Australia	03/18/2019	3C.2a1b+131K	Australia	EPI_ISL_354728
A/Newcastle/42/2019|Australia|03/07/2019	Australia	03/07/2019	3C.2a1b+131K	Australia	EPI_ISL_356799
A/Newcastle/44/2018|Australia|08/03/2018	Australia	08/03/2018	3C.2a2		EPI_ISL_322732
A/Newcastle/45/2019|Australia|03/18/2019	Australia	03/18/2019	3C.2a1b+131K	Australia	EPI_ISL_354729
A/Newcastle/48/2018|Australia|08/12/2018	Australia	08/12/2018	3C.2a1b+135N		EPI_ISL_323048
A/Newcastle/49/2019|Australia|03/17/2019	Australia	03/17/2019	3C.2a1b+131K	Australia	EPI_ISL_354771
A/Newcastle/5/2018|Australia|02/08/2018	Australia	02/08/2018	3C.2a2		EPI_ISL_312148
A/Newcastle/5/2019|Australia|01/17/2019	Australia	01/17/2019	3C.2a1b+131K	Australia	EPI_ISL_339081
A/Newcastle/50/2019|Australia|03/14/2019	Australia	03/14/2019	3C.2a1b+131K	Australia	EPI_ISL_353804
A/Newcastle/51/2019|Australia|03/17/2019	Australia	03/17/2019	3C.3a	Australia	EPI_ISL_354720
A/Newcastle/55/2018|Australia|09/04/2018	Australia	09/04/2018	3C.2a1b+135K	Australia	EPI_ISL_332738
A/Newcastle/56/2018|Australia|09/03/2018	Australia	09/03/2018	3C.2a2	Australia	EPI_ISL_332739
A/Newcastle/6/2019|Australia|01/19/2019	Australia	01/19/2019	3C.2a1b+135K	Australia	EPI_ISL_346328
A/Newcastle/602/2019|Australia|01/17/2019	Australia	01/17/2019	3C.2a1b+131K	Australia	EPI_ISL_346333
A/Newcastle/604/2019|Australia|01/19/2019	Australia	01/19/2019	3C.2a1b+131K	Australia	EPI_ISL_346334
A/Newcastle/605/2019|Australia|01/20/2019	Australia	01/20/2019	3C.2a1b+131K	Australia	EPI_ISL_346338

A/Newcastle/606/2019|Australia|01/18/2019	Australia	01/18/2019	3C.2a1b+131K	Australia	EPI_ISL_346335
A/Newcastle/607/2019|Australia|01/09/2019	Australia	01/09/2019	3C.2a1b+131K	Australia	EPI_ISL_346326
A/Newcastle/610/2019|Australia|01/16/2019	Australia	01/16/2019	3C.2a1b+131K	Australia	EPI_ISL_346336
A/Newcastle/613/2019|Australia|01/18/2019	Australia	01/18/2019	3C.2a1b+135N	Australia	EPI_ISL_346337
A/Newcastle/623/2019|Australia|02/26/2019	Australia	02/26/2019	3C.3a	Australia	EPI_ISL_356800
A/Newcastle/627/2019|Australia|03/19/2019	Australia	03/19/2019	3C.2a1b+131K	Australia	EPI_ISL_356622
A/Newcastle/631/2019|Australia|03/16/2019	Australia	03/16/2019	3C.3a	Australia	EPI_ISL_353799
A/Newcastle/633/2019|Australia|03/04/2019	Australia	03/04/2019	3C.2a1b+131K	Australia	EPI_ISL_356670
A/Newcastle/7/2018|Australia|04/01/2018	Australia	04/01/2018	3C.2a2		EPI_ISL_320377
A/Newcastle/7/2019|Australia|01/18/2019	Australia	01/18/2019	3C.2a1b+135K	Australia	EPI_ISL_346329
A/Newcastle/79/2018|Australia|12/27/2018	Australia	12/27/2018	3C.2a1b+131K	Australia	EPI_ISL_339280
A/Newcastle/82/2018|Australia|12/23/2018	Australia	12/23/2018	3C.2a1b+131K	Australia	EPI_ISL_339348
A/Newcastle/83/2018|Australia|12/25/2018	Australia	12/25/2018	3C.2a1b+131K	Australia	EPI_ISL_339349
A/Newcastle/84/2018|Australia|12/23/2018	Australia	12/23/2018	3C.2a1b+131K	Australia	EPI_ISL_339275
A/Newcastle/9/2018|Australia|02/21/2018	Australia	02/21/2018	3C.2a2		EPI_ISL_321457
A/Newcastle/9/2019|Australia|01/11/2019	Australia	01/11/2019	3C.2a1b+131K	Australia	EPI_ISL_346330
A/Newcastle/96/2018|Australia|12/16/2018	Australia	12/16/2018	3C.2a1b+135K	Australia	EPI_ISL_339245
A/Niedersachsen/117/2018|Europe|05/04/2018	Europe	05/04/2018	3C.2a1b+135K		EPI_ISL_312794
A/Nord_Pas_de_Calais/1750/2019|Europe|03/19/2019	Europe	03/19/2019	3C.3a	Global	EPI_ISL_353943
A/Northern_Ireland/12971/2018|Europe|04/05/2018	Europe	04/05/2018	3C.2a2		EPI_ISL_320650
A/Ohio/19/2019|NorthAmerica|04/10/2019	NorthAmerica	04/10/2019	3C.3a	Global	EPI_ISL_356244
A/Ohio/22/2018|NorthAmerica|06/23/2018	NorthAmerica	06/23/2018	3C.2a2		EPI_ISL_320303
A/Oman/2482/2019|Asia|03/09/2019	Asia	03/09/2019	3C.2a1b+135K	Global	EPI_ISL_355534
A/Oman/3610/2018|Asia|07/06/2018	Asia	07/06/2018	3C.2a1b+135K		EPI_ISL_356732
A/Oman/4994/2018|Asia|10/15/2018	Asia	10/15/2018	3C.2a1b+131K	Global	EPI_ISL_333702
A/Oman/5209/2018|Asia|10/20/2018	Asia	10/20/2018	3C.2a1b+135K	Global	EPI_ISL_333726
A/Oman/5606/2018|Asia|10/31/2018	Asia	10/31/2018	3C.2a1b+135K	Global	EPI_ISL_333735
A/Oman/5810/2018|Asia|11/07/2018	Asia	11/07/2018	3C.2a1b+131K	Global	EPI_ISL_333671
A/Oman/6241/2018|Asia|11/21/2018	Asia	11/21/2018	3C.2a1b+135K	Global	EPI_ISL_337935
A/Oman/6262/2018|Asia|11/10/2018	Asia	11/10/2018	3C.2a1b+135K	Global	EPI_ISL_332820
A/Ontario/RV0612/2019|NorthAmerica|01/09/2019	NorthAmerica	01/09/2019	3C.2a2	Global	EPI_ISL_339926
A/Ontario/RV1475/2019|NorthAmerica|03/06/2019	NorthAmerica	03/06/2019	3C.2a1b+131K	Global	EPI_ISL_350896
A/Ontario/RV2015/2019|NorthAmerica|05/03/2019	NorthAmerica	05/03/2019	3C.3a	Global	EPI_ISL_362010
A/Ontario/RV2142/2018|NorthAmerica|01/21/2018	NorthAmerica	01/21/2018	3C.2a2		EPI_ISL_304381

A/Ontario/RV2536/2018|NorthAmerica|02/14/2018	NorthAmerica	02/14/2018	3C.2a2		EPI_ISL_306596
A/Ontario/RV3022/2018|NorthAmerica|03/02/2018	NorthAmerica	03/02/2018	3C.2a2		EPI_ISL_308098
A/Ontario/RV3079/2018|NorthAmerica|02/02/2018	NorthAmerica	02/02/2018	3C.2a2		EPI_ISL_308119
A/Ontario/RV3393/2018|NorthAmerica|03/19/2018	NorthAmerica	03/19/2018	3C.3a		EPI_ISL_313025
A/Oregon/14/2019|NorthAmerica|03/06/2019	NorthAmerica	03/06/2019	3C.3a	Global	EPI_ISL_355601
A/OSAKA/2/2019|Asia|01/07/2019	Asia	01/07/2019	3C.2a1b+131K	Global	EPI_ISL_356444
A/Oujda/1113/2019|Africa|02/05/2019	Africa	02/05/2019	3C.2a1b+131K	Global	EPI_ISL_355094
A/Pakistan/490/2019|Asia|01/23/2019	Asia	01/23/2019	3C.2a1b+135K	Global	EPI_ISL_355543
A/Para/151642-IEC/2018|SouthAmerica|03/19/2018	SouthAmerica	03/19/2018	3C.2a2		EPI_ISL_328559
A/Para/153301-IEC/2018|SouthAmerica|05/18/2018	SouthAmerica	05/18/2018	3C.2a2		EPI_ISL_320309
A/Paraguay/1074/2018|SouthAmerica|07/15/2018	SouthAmerica	07/15/2018	3C.3a		EPI_ISL_331062
A/Paraguay/4199/2018|SouthAmerica|03/02/2018	SouthAmerica	03/02/2018	3C.2a2		EPI_ISL_321873
A/Paraguay/8607/2018|SouthAmerica|05/18/2018	SouthAmerica	05/18/2018	3C.3a		EPI_ISL_330919
A/Paraiba/151837-IEC/2018|SouthAmerica|03/28/2018	SouthAmerica	03/28/2018	3C.2a2		EPI_ISL_328546
A/Parana/1257/2018|SouthAmerica|09/02/2018	SouthAmerica	09/02/2018	3C.2a2	Global	EPI_ISL_345599
A/Parana/158/2018|SouthAmerica|03/22/2018	SouthAmerica	03/22/2018	3C.2a2		EPI_ISL_322258
A/Paris/1719/2018|Europe|04/09/2018	Europe	04/09/2018	3C.2a1b+135K		EPI_ISL_314677
A/Paris/2511/2018|Europe|11/29/2018	Europe	11/29/2018	3C.3a	Global	EPI_ISL_332706
A/Pennsylvania/362/2018|NorthAmerica|02/01/2018	NorthAmerica	02/01/2018	3C.2a2		EPI_ISL_361842
A/Pennsylvania/510/2018|NorthAmerica|12/16/2018	NorthAmerica	12/16/2018	3C.2a1b+135K	Global	EPI_ISL_338553
A/Pernambuco/151608-IEC/2018|SouthAmerica|02/16/2018	SouthAmerica	02/16/2018	3C.3a		EPI_ISL_329823
A/Perth/1/2019|Australia|01/07/2019	Australia	01/07/2019	3C.2a1b+135K	Australia	EPI_ISL_346325
A/Perth/1000/2019|Australia|01/14/2019	Australia	01/14/2019	3C.2a1b+131K	Australia	EPI_ISL_345229
A/Perth/1001/2018|Australia|06/07/2018	Australia	06/07/2018	3C.3a		EPI_ISL_320400
A/Perth/1003/2019|Australia|03/12/2019	Australia	03/12/2019	3C.2a1b+131K	Australia	EPI_ISL_356671
A/Perth/1004/2019|Australia|03/14/2019	Australia	03/14/2019	3C.2a1b+131K	Australia	EPI_ISL_356672
A/Perth/1008/2018|Australia|07/13/2018	Australia	07/13/2018	3C.3a		EPI_ISL_322332
A/Perth/1024/2018|Australia|09/06/2018	Australia	09/06/2018	3C.2a2	Australia	EPI_ISL_330114
A/Perth/1029/2018|Australia|09/21/2018	Australia	09/21/2018	3C.2a1b+135K	Australia	EPI_ISL_332732
A/Perth/103/2018|Australia|09/03/2018	Australia	09/03/2018	3C.3a	Australia	EPI_ISL_331777
A/Perth/1036/2018|Australia|10/18/2018	Australia	10/18/2018	3C.2a1b+135K	Australia	EPI_ISL_332649
A/Perth/12/2018|Australia|02/20/2018	Australia	02/20/2018	3C.2a2		EPI_ISL_313818
A/Perth/13/2018|Australia|02/20/2018	Australia	02/20/2018	3C.2a2		EPI_ISL_312182
A/Perth/13/2019|Australia|01/14/2019	Australia	01/14/2019	3C.2a1b+131K	Australia	EPI_ISL_346302

A/Perth/15/2019|Australia|01/01/2019	Australia	01/01/2019	3C.2a1b+131K	Australia	EPI_ISL_342173
A/Perth/151/2018|Australia|09/30/2018	Australia	09/30/2018	3C.2a2	Australia	EPI_ISL_334090
A/Perth/155/2018|Australia|10/01/2018	Australia	10/01/2018	3C.2a3	Australia	EPI_ISL_339075
A/Perth/16/2018|Australia|03/22/2018	Australia	03/22/2018	3C.2a1b+135K		EPI_ISL_321452
A/Perth/170/2018|Australia|10/02/2018	Australia	10/02/2018	3C.2a1b+135K	Australia	EPI_ISL_338268
A/Perth/172/2018|Australia|10/01/2018	Australia	10/01/2018	3C.2a3	Australia	EPI_ISL_339076
A/Perth/2/2018|Australia|01/28/2018	Australia	01/28/2018	3C.2a1b+135N		EPI_ISL_312181
A/Perth/43/2018|Australia|07/12/2018	Australia	07/12/2018	3C.2a2		EPI_ISL_323050
A/Perth/5/2019|Australia|01/09/2019	Australia	01/09/2019	3C.2a1b+131K	Australia	EPI_ISL_346301
A/Perth/9/2019|Australia|01/12/2019	Australia	01/12/2019	3C.2a1b+131K	Australia	EPI_ISL_346319
A/Peru/0918/2018|SouthAmerica|04/10/2018	SouthAmerica	04/10/2018	3C.2a2		EPI_ISL_322964
A/Peru/1418/2018|SouthAmerica|12/03/2018	SouthAmerica	12/03/2018	3C.2a1b+135K	Global	EPI_ISL_348171
A/Peru/2118/2018|SouthAmerica|04/09/2018	SouthAmerica	04/09/2018	3C.2a2		EPI_ISL_321908
A/Peru/3118/2018|SouthAmerica|12/05/2018	SouthAmerica	12/05/2018	3C.2a1b+135K	Global	EPI_ISL_348172
A/Peru/4718/2018|SouthAmerica|10/14/2018	SouthAmerica	10/14/2018	3C.2a2	Global	EPI_ISL_353704
A/Peru/769818/2018|SouthAmerica|10/22/2018	SouthAmerica	10/22/2018	3C.2a2	Global	EPI_ISL_348169
A/Phetchaburi/28/2018|Asia|02/01/2018	Asia	02/01/2018	3C.2a1b+135K		EPI_ISL_333862
A/Philippines/0464/2018|Asia|05/06/2018	Asia	05/06/2018	3C.2a2		EPI_ISL_322958
A/Philippines/40/2018|Asia|07/24/2018	Asia	07/24/2018	3C.2a1b+135N		EPI_ISL_330729
A/Poitiers/1977/2018|Europe|10/11/2018	Europe	10/11/2018	3C.2a1b+135K	Global	EPI_ISL_345745
A/Powys/0208/2018|Europe|02/26/2018	Europe	02/26/2018	3C.2a2		EPI_ISL_313997
A/Puerto_Montt/62121/2018|SouthAmerica|07/24/2018	SouthAmerica	07/24/2018	3C.2a1b+135K		EPI_ISL_347027
A/Puerto_Rico/21/2018|NorthAmerica|06/29/2018	NorthAmerica	06/29/2018	3C.2a2		EPI_ISL_330486
A/Punta_Arenas/102705/2018|SouthAmerica|11/30/2018	SouthAmerica	11/30/2018	3C.3a	Global	EPI_ISL_346129
A/Quebec/RV2946/2018|NorthAmerica|04/01/2018	NorthAmerica	04/01/2018	3C.3a		EPI_ISL_313515
A/Quebec/RV3649/2018|NorthAmerica|04/10/2018	NorthAmerica	04/10/2018	3C.3a		EPI_ISL_312440
A/Rabat/1229/2018|Africa|02/18/2018	Africa	02/18/2018	3C.2a1b+135K		EPI_ISL_314680
A/Rabat/1429/2019|Africa|02/07/2019	Africa	02/07/2019	3C.2a1b+135K	Global	EPI_ISL_355100
A/Rabat/799/2018|Africa|01/16/2018	Africa	01/16/2018	3C.2a1b+135K		EPI_ISL_308729
A/Rancagua/54068/2018|SouthAmerica|06/27/2018	SouthAmerica	06/27/2018	3C.2a2		EPI_ISL_330284
A/Rheinland-Pfalz/59/2019|Europe|04/29/2019	Europe	04/29/2019	3C.2a1b+135K	Global	EPI_ISL_356701
A/Rio_Grande_Do_Sul/27/2018|SouthAmerica|01/04/2018	SouthAmerica	01/04/2018	3C.2a2		EPI_ISL_330263
A/Saarland/18/2018|Europe|03/28/2018	Europe	03/28/2018	3C.2a2		EPI_ISL_311297
A/Samsun/910/2018|Asia|12/26/2018	Asia	12/26/2018	3C.2a1b+135K	Global	EPI_ISL_341331

A/Santa_Catarina/24/2018|SouthAmerica|01/05/2018	SouthAmerica	01/05/2018	3C.2a2		EPI_ISL_301370
A/Santa_Catarina/256/2018|SouthAmerica|04/15/2018	SouthAmerica	04/15/2018	3C.3a		EPI_ISL_322292
A/Santa_Catarina/333/2018|SouthAmerica|04/23/2018	SouthAmerica	04/23/2018	3C.2a2		EPI_ISL_322293
A/Santa_Catarina/62/2018|SouthAmerica|02/14/2018	SouthAmerica	02/14/2018	3C.2a2		EPI_ISL_306037
A/Santiago/103008/2018|SouthAmerica|11/29/2018	SouthAmerica	11/29/2018	3C.3a	Global	EPI_ISL_346130
A/Santiago/103167/2018|SouthAmerica|11/29/2018	SouthAmerica	11/29/2018	3C.3a	Global	EPI_ISL_346131
A/Santiago/21659/2019|SouthAmerica|03/06/2019	SouthAmerica	03/06/2019	3C.2a1b+135K	Global	EPI_ISL_355850
A/Santiago/58712/2018|SouthAmerica|07/17/2018	SouthAmerica	07/17/2018	3C.2a2		EPI_ISL_347008
A/Santiago/69717/2018|SouthAmerica|08/20/2018	SouthAmerica	08/20/2018	3C.2a2		EPI_ISL_347039
A/Santiago/79234/2018|SouthAmerica|09/14/2018	SouthAmerica	09/14/2018	3C.2a2	Global	EPI_ISL_347068
A/Santiago/9435/2018|SouthAmerica|02/01/2018	SouthAmerica	02/01/2018	3C.2a2		EPI_ISL_315097
A/SAPPORO/45/2018|Asia|05/01/2018	Asia	05/01/2018	3C.2a2		EPI_ISL_322114
A/Saskatchewan/RV1856/2019|NorthAmerica|04/21/2019	NorthAmerica	04/21/2019	3C.3a	Global	EPI_ISL_355928
A/Saskatchewan/RV1859/2019|NorthAmerica|04/15/2019	NorthAmerica	04/15/2019	3C.2a1b+131K	Global	EPI_ISL_355929
A/Saskatchewan/RV3932/2018|NorthAmerica|09/12/2018	NorthAmerica	09/12/2018	3C.2a1b+135K	Global	EPI_ISL_332953
A/Saskatchewan/RV4128/2018|NorthAmerica|11/24/2018	NorthAmerica	11/24/2018	3C.2a1b+135K	Global	EPI_ISL_339843
A/Saudi_Arabia/393/2018|Asia|05/21/2018	Asia	05/21/2018	3C.2a1b+135K		EPI_ISL_332901
A/Saudi_Arabia/459160/2018|Asia|08/11/2018	Asia	08/11/2018	3C.2a1b+135K		EPI_ISL_355104
A/Saudi_Arabia/461280/2018|Asia|08/18/2018	Asia	08/18/2018	3C.2a1b+135K		EPI_ISL_355106
A/Singapore/GP0997/2018|Asia|04/24/2018	Asia	04/24/2018	3C.2a2		EPI_ISL_322104
A/Singapore/NTF0033/2018|Asia|07/18/2018	Asia	07/18/2018	3C.2a1b+135N		EPI_ISL_340481
A/Singapore/TT0043/2018|Asia|01/11/2018	Asia	01/11/2018	3C.2a3		EPI_ISL_322080
A/Singapore/TT0256/2018|Asia|02/14/2018	Asia	02/14/2018	3C.2a1b+135N		EPI_ISL_322091
A/Slovenia/1088/2019|Europe|05/14/2019	Europe	05/14/2019	3C.2a1b+135K	Global	EPI_ISL_357325
A/South_Africa/R00489/2018|Africa|01/16/2018	Africa	01/16/2018	3C.2a1b+135N		EPI_ISL_320594
A/South_Australia/1/2018|Australia|01/04/2018	Australia	01/04/2018	3C.2a1b+135N		EPI_ISL_314614
A/South_Australia/1/2019|Australia|01/02/2019	Australia	01/02/2019	3C.2a3	Australia	EPI_ISL_346309
A/South_Australia/10/2018|Australia|04/03/2018	Australia	04/03/2018	3C.2a2		EPI_ISL_320966
A/South_Australia/1000/2019|Australia|01/10/2019	Australia	01/10/2019	3C.2a1b+131K	Australia	EPI_ISL_345230
A/South_Australia/1001/2019|Australia|02/04/2019	Australia	02/04/2019	3C.2a1b+131K	Australia	EPI_ISL_346267
A/South_Australia/1002/2019|Australia|02/26/2019	Australia	02/26/2019	3C.2a1b+131K	Australia	EPI_ISL_354777
A/South_Australia/1003/2019|Australia|02/25/2019	Australia	02/25/2019	3C.2a1b+131K	Australia	EPI_ISL_354776
A/South_Australia/1004/2018|Australia|07/09/2018	Australia	07/09/2018	3C.2a1b+135N		EPI_ISL_321453
A/South_Australia/1004/2019|Australia|02/26/2019	Australia	02/26/2019	3C.2a1b+131K	Australia	EPI_ISL_354747

A/South_Australia/1006/2018|Australia|07/18/2018	Australia	07/18/2018	3C.2a1b+135N		EPI_ISL_322340
A/South_Australia/1007/2018|Australia|07/18/2018	Australia	07/18/2018	3C.2a1b+135N		EPI_ISL_322341
A/South_Australia/1007/2019|Australia|03/01/2019	Australia	03/01/2019	3C.2a1b+131K	Australia	EPI_ISL_354748
A/South_Australia/1008/2019|Australia|03/05/2019	Australia	03/05/2019	3C.2a1b+131K	Australia	EPI_ISL_354749
A/South_Australia/1009/2018|Australia|07/17/2018	Australia	07/17/2018	3C.2a1b+135N		EPI_ISL_322342
A/South_Australia/101/2019|Australia|02/28/2019	Australia	02/28/2019	3C.2a1b+131K	Australia	EPI_ISL_354756
A/South_Australia/1017/2018|Australia|09/21/2018	Australia	09/21/2018	3C.2a1b+135N	Australia	EPI_ISL_332731
A/South_Australia/1023/2018|Australia|09/28/2018	Australia	09/28/2018	3C.2a1b+135K	Australia	EPI_ISL_332733
A/South_Australia/1028/2018|Australia|10/13/2018	Australia	10/13/2018	3C.2a1b+135K	Australia	EPI_ISL_332647
A/South_Australia/103/2019|Australia|02/25/2019	Australia	02/25/2019	3C.2a1b+131K	Australia	EPI_ISL_354775
A/South_Australia/106/2019|Australia|02/28/2019	Australia	02/28/2019	3C.2a1b+135K	Australia	EPI_ISL_354757
A/South_Australia/1060/2019|Australia|05/01/2019	Australia	05/01/2019	3C.2a1b+131K	Australia	EPI_ISL_362096
A/South_Australia/108/2019|Australia|02/27/2019	Australia	02/27/2019	3C.2a1b+131K	Australia	EPI_ISL_354758
A/South_Australia/109/2019|Australia|02/25/2019	Australia	02/25/2019	3C.2a1b+135K	Australia	EPI_ISL_354735
A/South_Australia/11/2018|Australia|04/03/2018	Australia	04/03/2018	3C.2a2		EPI_ISL_313815
A/South_Australia/111/2019|Australia|02/23/2019	Australia	02/23/2019	3C.2a1b+131K	Australia	EPI_ISL_354759
A/South_Australia/112/2018|Australia|10/05/2018	Australia	10/05/2018	3C.2a2	Australia	EPI_ISL_341296
A/South_Australia/112/2019|Australia|03/04/2019	Australia	03/04/2019	3C.2a1b+131K	Australia	EPI_ISL_354736
A/South_Australia/113/2019|Australia|03/08/2019	Australia	03/08/2019	3C.2a1b+131K	Australia	EPI_ISL_354760
A/South_Australia/114/2019|Australia|03/06/2019	Australia	03/06/2019	3C.2a1b+131K	Australia	EPI_ISL_354761
A/South_Australia/115/2019|Australia|03/02/2019	Australia	03/02/2019	3C.2a1b+131K	Australia	EPI_ISL_354762
A/South_Australia/116/2018|Australia|10/08/2018	Australia	10/08/2018	3C.2a1b+135N	Australia	EPI_ISL_332793
A/South_Australia/117/2018|Australia|10/08/2018	Australia	10/08/2018	3C.2a1b+135N	Australia	EPI_ISL_340098
A/South_Australia/117/2019|Australia|03/03/2019	Australia	03/03/2019	3C.2a1b+131K	Australia	EPI_ISL_354763
A/South_Australia/118/2019|Australia|03/09/2019	Australia	03/09/2019	3C.2a1b+131K	Australia	EPI_ISL_354737
A/South_Australia/119/2019|Australia|03/06/2019	Australia	03/06/2019	3C.2a1b+131K	Australia	EPI_ISL_354764
A/South_Australia/12/2018|Australia|05/29/2018	Australia	05/29/2018	3C.2a1b+135K		EPI_ISL_320398
A/South_Australia/12/2019|Australia|01/05/2019	Australia	01/05/2019	3C.2a1b+131K	Australia	EPI_ISL_346314
A/South_Australia/120/2018|Australia|10/09/2018	Australia	10/09/2018	3C.2a1b+135N	Australia	EPI_ISL_340099
A/South_Australia/123/2018|Australia|10/10/2018	Australia	10/10/2018	3C.2a1b+131K	Australia	EPI_ISL_332656
A/South_Australia/123/2019|Australia|03/06/2019	Australia	03/06/2019	3C.2a1b+131K	Australia	EPI_ISL_354765
A/South_Australia/124/2019|Australia|03/05/2019	Australia	03/05/2019	3C.2a1b+131K	Australia	EPI_ISL_354738
A/South_Australia/125/2018|Australia|10/09/2018	Australia	10/09/2018	3C.2a2	Australia	EPI_ISL_332657
A/South_Australia/126/2019|Australia|03/05/2019	Australia	03/05/2019	3C.2a1b+131K	Australia	EPI_ISL_354774

A/South_Australia/13/2018|Australia|05/29/2018	Australia	05/29/2018	3C.2a2		EPI_ISL_320397
A/South_Australia/13/2019|Australia|01/11/2019	Australia	01/11/2019	3C.2a1b+131K	Australia	EPI_ISL_346315
A/South_Australia/134/2018|Australia|10/15/2018	Australia	10/15/2018	3C.2a2	Australia	EPI_ISL_341297
A/South_Australia/135/2018|Australia|10/17/2018	Australia	10/17/2018	3C.2a1b+131K	Australia	EPI_ISL_340097
A/South_Australia/135/2019|Australia|03/05/2019	Australia	03/05/2019	3C.2a1b+131K	Australia	EPI_ISL_354773
A/South_Australia/137/2019|Australia|03/03/2019	Australia	03/03/2019	3C.2a1b+131K	Australia	EPI_ISL_354772
A/South_Australia/138/2019|Australia|03/06/2019	Australia	03/06/2019	3C.2a1b+131K	Australia	EPI_ISL_354739
A/South_Australia/139/2019|Australia|03/15/2019	Australia	03/15/2019	3C.2a1b+131K	Australia	
A/South_Australia/14/2019|Australia|01/16/2019	Australia	01/16/2019	3C.2a1b+131K	Australia	EPI_ISL_346321
A/South_Australia/141/2019|Australia|03/03/2019	Australia	03/03/2019	3C.2a1b+131K	Australia	EPI_ISL_354730
A/South_Australia/142/2019|Australia|03/04/2019	Australia	03/04/2019	3C.2a1b+131K	Australia	EPI_ISL_354731
A/South_Australia/143/2018|Australia|10/22/2018	Australia	10/22/2018	3C.2a1b+131K	Australia	EPI_ISL_332651
A/South_Australia/146/2019|Australia|03/12/2019	Australia	03/12/2019	3C.2a1b+131K	Australia	EPI_ISL_356673
A/South_Australia/147/2019|Australia|03/09/2019	Australia	03/09/2019	3C.2a1b+131K	Australia	EPI_ISL_356674
A/South_Australia/148/2019|Australia|03/14/2019	Australia	03/14/2019	3C.2a1b+131K	Australia	EPI_ISL_356675
A/South_Australia/149/2019|Australia|03/13/2019	Australia	03/13/2019	3C.2a1b+131K	Australia	EPI_ISL_356676
A/South_Australia/15/2019|Australia|01/15/2019	Australia	01/15/2019	3C.2a1b+131K	Australia	EPI_ISL_346322
A/South_Australia/151/2019|Australia|03/10/2019	Australia	03/10/2019	3C.2a1b+131K	Australia	EPI_ISL_356677
A/South_Australia/153/2018|Australia|11/01/2018	Australia	11/01/2018	3C.2a1b+135K	Australia	EPI_ISL_340092
A/South_Australia/153/2019|Australia|03/13/2019	Australia	03/13/2019	3C.2a1b+131K	Australia	EPI_ISL_356678
A/South_Australia/155/2019|Australia|03/10/2019	Australia	03/10/2019	3C.2a1b+131K	Australia	EPI_ISL_356679
A/South_Australia/156/2019|Australia|03/15/2019	Australia	03/15/2019	3C.2a1b+131K	Australia	
A/South_Australia/159/2018|Australia|11/03/2018	Australia	11/03/2018	3C.2a1b+131K	Australia	EPI_ISL_334088
A/South_Australia/159/2019|Australia|03/15/2019	Australia	03/15/2019	3C.2a1b+131K	Australia	EPI_ISL_356639
A/South_Australia/160/2018|Australia|11/06/2018	Australia	11/06/2018	3C.2a1b+135K	Australia	EPI_ISL_338274
A/South_Australia/160/2019|Australia|03/08/2019	Australia	03/08/2019	3C.2a1b+131K	Australia	EPI_ISL_356640
A/South_Australia/161/2018|Australia|11/04/2018	Australia	11/04/2018	3C.2a1b+135N	Australia	EPI_ISL_340095
A/South_Australia/161/2019|Australia|03/14/2019	Australia	03/14/2019	3C.2a1b+131K	Australia	EPI_ISL_356641
A/South_Australia/162/2019|Australia|03/09/2019	Australia	03/09/2019	3C.2a1b+135K	Australia	EPI_ISL_356631
A/South_Australia/163/2019|Australia|03/12/2019	Australia	03/12/2019	3C.2a1b+131K	Australia	EPI_ISL_356642
A/South_Australia/164/2018|Australia|11/05/2018	Australia	11/05/2018	3C.2a1b+135N	Australia	EPI_ISL_336143
A/South_Australia/167/2018|Australia|11/04/2018	Australia	11/04/2018	3C.2a1b+135K	Australia	EPI_ISL_340093
A/South_Australia/169/2019|Australia|03/11/2019	Australia	03/11/2019	3C.2a1b+131K	Australia	EPI_ISL_356643
A/South_Australia/171/2018|Australia|11/06/2018	Australia	11/06/2018	3C.2a1b+135K	Australia	EPI_ISL_340094

A/South_Australia/171/2019|Australia|03/14/2019	Australia	03/14/2019	3C.2a1b+131K	Australia	EPI_ISL_356644
A/South_Australia/172/2019|Australia|03/11/2019	Australia	03/11/2019	3C.2a1b+131K	Australia	EPI_ISL_356645
A/South_Australia/173/2019|Australia|03/15/2019	Australia	03/15/2019	3C.2a1b+131K	Australia	EPI_ISL_356646
A/South_Australia/174/2018|Australia|11/06/2018	Australia	11/06/2018	3C.2a1b+135K	Australia	EPI_ISL_338270
A/South_Australia/174/2019|Australia|03/15/2019	Australia	03/15/2019	3C.2a1b+131K	Australia	EPI_ISL_356647
A/South_Australia/175/2019|Australia|03/13/2019	Australia	03/13/2019	3C.2a1b+131K	Australia	EPI_ISL_356648
A/South_Australia/176/2018|Australia|11/03/2018	Australia	11/03/2018	3C.2a1b+131K	Australia	EPI_ISL_336141
A/South_Australia/177/2018|Australia|11/14/2018	Australia	11/14/2018	3C.2a2	Australia	EPI_ISL_338265
A/South_Australia/178/2019|Australia|03/16/2019	Australia	03/16/2019	3C.2a1b+131K	Australia	EPI_ISL_356680
A/South_Australia/179/2018|Australia|11/16/2018	Australia	11/16/2018	3C.2a2	Australia	EPI_ISL_338266
A/South_Australia/179/2019|Australia|03/15/2019	Australia	03/15/2019	3C.2a1b+131K	Australia	
A/South_Australia/18/2019|Australia|01/14/2019	Australia	01/14/2019	3C.2a3	Australia	EPI_ISL_346316
A/South_Australia/180/2019|Australia|03/15/2019	Australia	03/15/2019	3C.2a1b+131K	Australia	
A/South_Australia/181/2018|Australia|11/14/2018	Australia	11/14/2018	3C.2a3	Australia	EPI_ISL_338267
A/South_Australia/182/2019|Australia|03/15/2019	Australia	03/15/2019	3C.2a1b+131K	Australia	
A/South_Australia/184/2018|Australia|11/10/2018	Australia	11/10/2018	3C.2a1b+131K	Australia	EPI_ISL_339055
A/South_Australia/186/2018|Australia|11/14/2018	Australia	11/14/2018	3C.2a1b+135N	Australia	EPI_ISL_339072
A/South_Australia/186/2019|Australia|03/18/2019	Australia	03/18/2019	3C.2a1b+131K	Australia	EPI_ISL_356649
A/South_Australia/187/2019|Australia|03/19/2019	Australia	03/19/2019	3C.2a1b+131K	Australia	EPI_ISL_356650
A/South_Australia/188/2019|Australia|03/16/2019	Australia	03/16/2019	3C.2a1b+131K	Australia	EPI_ISL_356651
A/South_Australia/189/2018|Australia|11/18/2018	Australia	11/18/2018	3C.2a2	Australia	EPI_ISL_339073
A/South_Australia/189/2019|Australia|03/19/2019	Australia	03/19/2019	3C.2a1b+131K	Australia	EPI_ISL_356652
A/South_Australia/19/2018|Australia|07/10/2018	Australia	07/10/2018	3C.2a1b+135N		EPI_ISL_321588
A/South_Australia/19/2019|Australia|01/17/2019	Australia	01/17/2019	3C.2a1b+131K	Australia	EPI_ISL_346317
A/South_Australia/192/2019|Australia|03/15/2019	Australia	03/15/2019	3C.2a1b+131K	Australia	EPI_ISL_356653
A/South_Australia/194/2019|Australia|03/20/2019	Australia	03/20/2019	3C.2a1b+131K	Australia	EPI_ISL_356654
A/South_Australia/197/2019|Australia|03/20/2019	Australia	03/20/2019	3C.2a1b+131K	Australia	EPI_ISL_356655
A/South_Australia/198/2019|Australia|03/19/2019	Australia	03/19/2019	3C.2a1b+131K	Australia	EPI_ISL_356656
A/South_Australia/199/2018|Australia|11/20/2018	Australia	11/20/2018	3C.2a1b+135K	Australia	EPI_ISL_334087
A/South_Australia/199/2019|Australia|03/17/2019	Australia	03/17/2019	3C.2a1b+131K	Australia	EPI_ISL_356657
A/South_Australia/2/2018|Australia|01/06/2018	Australia	01/06/2018	3C.2a2		EPI_ISL_314620
A/South_Australia/2/2019|Australia|01/04/2019	Australia	01/04/2019	3C.2a1b+135K	Australia	EPI_ISL_346310
A/South_Australia/201/2019|Australia|03/19/2019	Australia	03/19/2019	3C.2a1b+131K	Australia	EPI_ISL_356658
A/South_Australia/203/2019|Australia|03/20/2019	Australia	03/20/2019	3C.2a1b+131K	Australia	EPI_ISL_356659

A/South_Australia/204/2019|Australia|03/20/2019	Australia	03/20/2019	3C.2a1b+131K	Australia	EPI_ISL_356660
A/South_Australia/206/2019|Australia|03/15/2019	Australia	03/15/2019	3C.2a1b+131K	Australia	EPI_ISL_356661
A/South_Australia/207/2019|Australia|03/20/2019	Australia	03/20/2019	3C.2a1b+131K	Australia	EPI_ISL_356662
A/South_Australia/209/2019|Australia|03/18/2019	Australia	03/18/2019	3C.2a1b+131K	Australia	EPI_ISL_356663
A/South_Australia/210/2019|Australia|03/22/2019	Australia	03/22/2019	3C.2a1b+131K	Australia	EPI_ISL_356664
A/South_Australia/213/2019|Australia|03/21/2019	Australia	03/21/2019	3C.2a1b+131K	Australia	EPI_ISL_356665
A/South_Australia/214/2018|Australia|12/01/2018	Australia	12/01/2018	3C.2a2	Australia	EPI_ISL_342175
A/South_Australia/214/2019|Australia|03/18/2019	Australia	03/18/2019	3C.2a1b+131K	Australia	EPI_ISL_356666
A/South_Australia/217/2019|Australia|03/15/2019	Australia	03/15/2019	3C.2a1b+131K	Australia	EPI_ISL_356667
A/South_Australia/218/2019|Australia|03/22/2019	Australia	03/22/2019	3C.3a	Australia	EPI_ISL_353807
A/South_Australia/219/2019|Australia|03/22/2019	Australia	03/22/2019	3C.2a1b+131K	Australia	EPI_ISL_356668
A/South_Australia/22/2018|Australia|08/01/2018	Australia	08/01/2018			EPI_ISL_323115
A/South_Australia/22/2019|Australia|01/12/2019	Australia	01/12/2019	3C.2a1b+131K	Australia	EPI_ISL_346318
A/South_Australia/233/2018|Australia|12/17/2018	Australia	12/17/2018	3C.2a1b+135K	Australia	EPI_ISL_339234
A/South_Australia/24/2019|Australia|01/14/2019	Australia	01/14/2019	3C.2a1b+131K	Australia	EPI_ISL_346323
A/South_Australia/243/2018|Australia|12/27/2018	Australia	12/27/2018	3C.2a1b+131K	Australia	EPI_ISL_339269
A/South_Australia/250/2018|Australia|12/28/2018	Australia	12/28/2018	3C.2a1b+131K	Australia	EPI_ISL_339270
A/South_Australia/26/2018|Australia|08/12/2018	Australia	08/12/2018	3C.2a1b+131K		EPI_ISL_323116
A/South_Australia/28/2018|Australia|08/18/2018	Australia	08/18/2018	3C.2a1b+135N		EPI_ISL_330728
A/South_Australia/3/2018|Australia|01/08/2018	Australia	01/08/2018	3C.2a2		EPI_ISL_314613
A/South_Australia/33/2019|Australia|02/06/2019	Australia	02/06/2019	3C.2a1b+135K	Australia	EPI_ISL_346282
A/South_Australia/34/2019|Australia|02/06/2019	Australia	02/06/2019	3C.2a1b+131K	Australia	EPI_ISL_355880
A/South_Australia/36/2019|Australia|02/07/2019	Australia	02/07/2019	3C.2a1b+131K	Australia	EPI_ISL_355881
A/South_Australia/39/2019|Australia|02/08/2019	Australia	02/08/2019	3C.2a1b+131K	Australia	EPI_ISL_355882
A/South_Australia/4/2018|Australia|01/16/2018	Australia	01/16/2018	3C.2a2		EPI_ISL_314616
A/South_Australia/4/2019|Australia|01/01/2019	Australia	01/01/2019	3C.2a1b+135K	Australia	EPI_ISL_342174
A/South_Australia/40/2019|Australia|02/05/2019	Australia	02/05/2019	3C.2a1b+135K	Australia	EPI_ISL_346286
A/South_Australia/43/2019|Australia|02/01/2019	Australia	02/01/2019	3C.2a1b+135K	Australia	EPI_ISL_346287
A/South_Australia/44/2019|Australia|02/10/2019	Australia	02/10/2019	3C.2a1b+131K	Australia	EPI_ISL_354502
A/South_Australia/45/2019|Australia|02/13/2019	Australia	02/13/2019	3C.2a1b+131K	Australia	EPI_ISL_354500
A/South_Australia/5/2018|Australia|01/24/2018	Australia	01/24/2018	3C.2a2		EPI_ISL_314619
A/South_Australia/51/2019|Australia|02/17/2019	Australia	02/17/2019	3C.2a1b+131K	Australia	EPI_ISL_351029
A/South_Australia/53/2018|Australia|09/13/2018	Australia	09/13/2018	3C.2a1b+135N	Australia	EPI_ISL_330115
A/South_Australia/56/2019|Australia|02/17/2019	Australia	02/17/2019	3C.2a1b+135K	Australia	EPI_ISL_354444

A/South_Australia/6/2018|Australia|01/22/2018	Australia	01/22/2018	3C.2a2		EPI_ISL_314612
A/South_Australia/6/2019|Australia|01/04/2019	Australia	01/04/2019	3C.2a1b+131K	Australia	EPI_ISL_346311
A/South_Australia/63/2019|Australia|02/17/2019	Australia	02/17/2019	3C.2a1b+131K	Australia	EPI_ISL_354459
A/South_Australia/69/2019|Australia|01/21/2019	Australia	01/21/2019	3C.2a1b+131K	Australia	EPI_ISL_346268
A/South_Australia/7/2018|Australia|03/11/2018	Australia	03/11/2018	3C.2a1b+135N		EPI_ISL_314611
A/South_Australia/7/2019|Australia|01/07/2019	Australia	01/07/2019	3C.2a1b+131K	Australia	EPI_ISL_346312
A/South_Australia/70/2019|Australia|01/21/2019	Australia	01/21/2019	3C.2a1b+131K	Australia	EPI_ISL_346269
A/South_Australia/72/2019|Australia|01/24/2019	Australia	01/24/2019	3C.2a1b+131K	Australia	EPI_ISL_346270
A/South_Australia/74/2019|Australia|01/23/2019	Australia	01/23/2019	3C.2a2	Australia	EPI_ISL_346271
A/South_Australia/75/2018|Australia|09/21/2018	Australia	09/21/2018	3C.2a1b+135N	Australia	EPI_ISL_332740
A/South_Australia/76/2019|Australia|01/19/2019	Australia	01/19/2019	3C.2a1b+131K	Australia	EPI_ISL_346272
A/South_Australia/77/2019|Australia|01/22/2019	Australia	01/22/2019	3C.2a1b+131K	Australia	EPI_ISL_346273
A/South_Australia/78/2019|Australia|01/18/2019	Australia	01/18/2019	3C.2a1b+135K	Australia	EPI_ISL_346274
A/South_Australia/8/2018|Australia|03/17/2018	Australia	03/17/2018	3C.2a2		EPI_ISL_313810
A/South_Australia/8/2019|Australia|01/08/2019	Australia	01/08/2019	3C.2a1b+131K	Australia	EPI_ISL_346320
A/South_Australia/80/2019|Australia|01/24/2019	Australia	01/24/2019	3C.2a1b+131K	Australia	EPI_ISL_346275
A/South_Australia/81/2019|Australia|01/19/2019	Australia	01/19/2019	3C.2a1b+131K	Australia	EPI_ISL_346276
A/South_Australia/82/2018|Australia|09/17/2018	Australia	09/17/2018	3C.2a2	Australia	EPI_ISL_332741
A/South_Australia/82/2019|Australia|01/21/2019	Australia	01/21/2019	3C.2a1b+131K	Australia	EPI_ISL_355883
A/South_Australia/84/2019|Australia|01/28/2019	Australia	01/28/2019	3C.2a1b+131K	Australia	EPI_ISL_346278
A/South_Australia/85/2019|Australia|01/27/2019	Australia	01/27/2019	3C.2a1b+131K	Australia	EPI_ISL_346279
A/South_Australia/86/2019|Australia|01/27/2019	Australia	01/27/2019	3C.2a2	Australia	EPI_ISL_346280
A/South_Australia/87/2019|Australia|01/29/2019	Australia	01/29/2019	3C.2a1b+135K	Australia	EPI_ISL_346281
A/South_Australia/88/2019|Australia|01/30/2019	Australia	01/30/2019	3C.2a1b+131K	Australia	EPI_ISL_346258
A/South_Australia/9/2019|Australia|01/07/2019	Australia	01/07/2019	3C.2a1b+131K	Australia	EPI_ISL_346313
A/South_Australia/93/2019|Australia|03/01/2019	Australia	03/01/2019	3C.2a1b+131K	Australia	EPI_ISL_354751
A/South_Australia/94/2019|Australia|02/27/2019	Australia	02/27/2019	3C.2a1b+131K	Australia	EPI_ISL_354752
A/South_Australia/96/2019|Australia|02/26/2019	Australia	02/26/2019	3C.2a1b+131K	Australia	EPI_ISL_354753
A/South_Australia/97/2019|Australia|02/23/2019	Australia	02/23/2019	3C.2a1b+131K	Australia	EPI_ISL_354754
A/South_Australia/98/2018|Australia|09/28/2018	Australia	09/28/2018	3C.2a2	Australia	EPI_ISL_332742
A/South_Australia/99/2019|Australia|02/28/2019	Australia	02/28/2019	3C.2a1b+135K	Australia	EPI_ISL_354755
A/South_Dakota/04/2019|NorthAmerica|01/13/2019	NorthAmerica	01/13/2019	3C.2a1b+131K	Global	EPI_ISL_344628
A/South_Dakota/53/2018|NorthAmerica|11/27/2018	NorthAmerica	11/27/2018	3C.2a1b+131K	Global	EPI_ISL_334135
A/St_Petersburg/RII-530/2018|Europe|05/03/2018	Europe	05/03/2018	3C.2a2		EPI_ISL_330032

A/Stavropol/2V/2018|Europe|09/07/2018	Europe	09/07/2018	3C.2a1b+135K	Global	EPI_ISL_331623
A/Stockholm/20/2018|Europe|09/05/2018	Europe	09/05/2018	3C.2a1b+135K	Global	EPI_ISL_329741
A/Stockholm/22/2018|Europe|09/30/2018	Europe	09/30/2018	3C.2a1b+135K	Global	EPI_ISL_332334
A/Surat_Thani/83/2018|Asia|04/02/2018	Asia	04/02/2018	3C.2a2		EPI_ISL_329807
A/Sydney/10/2018|Australia|01/21/2018	Australia	01/21/2018	3C.2a2		EPI_ISL_312165
A/Sydney/10/2019|Australia|01/13/2019	Australia	01/13/2019	3C.2a1b+131K	Australia	EPI_ISL_346290
A/Sydney/100/2018|Australia|06/25/2018	Australia	06/25/2018	3C.2a1b+135N		EPI_ISL_331773
A/Sydney/1000/2018|Australia|03/14/2018	Australia	03/14/2018	3C.2a1b+135K		EPI_ISL_313813
A/Sydney/1002/2018|Australia|06/08/2018	Australia	06/08/2018	3C.2a1b+135K		EPI_ISL_320399
A/Sydney/1006/2019|Australia|01/14/2019	Australia	01/14/2019	3C.2a1b+135K	Australia	EPI_ISL_345228
A/Sydney/1008/2019|Australia|02/18/2019	Australia	02/18/2019	3C.2a1b+131K	Australia	EPI_ISL_354460
A/Sydney/1011/2019|Australia|03/04/2019	Australia	03/04/2019	3C.2a1b+131K	Australia	EPI_ISL_354750
A/Sydney/1017/2018|Australia|08/22/2018	Australia	08/22/2018	3C.2a2		EPI_ISL_331783
A/Sydney/1020/2018|Australia|08/29/2018	Australia	08/29/2018	3C.2a1b+135N		EPI_ISL_330731
A/Sydney/1038/2018|Australia|11/09/2018	Australia	11/09/2018	3C.2a2	Australia	EPI_ISL_339056
A/Sydney/11/2018|Australia|01/19/2018	Australia	01/19/2018	3C.2a2		EPI_ISL_312166
A/Sydney/11/2019|Australia|01/14/2019	Australia	01/14/2019	3C.2a1b+131K	Australia	EPI_ISL_346291
A/Sydney/12/2018|Australia|01/13/2018	Australia	01/13/2018	3C.2a1b+135N		EPI_ISL_312167
A/Sydney/12/2019|Australia|01/15/2019	Australia	01/15/2019	3C.2a1b+131K	Australia	EPI_ISL_346260
A/Sydney/121/2018|Australia|08/29/2018	Australia	08/29/2018	3C.2a1b+135N		EPI_ISL_331774
A/Sydney/13/2018|Australia|01/03/2018	Australia	01/03/2018	3C.2a1b+135K		EPI_ISL_312168
A/Sydney/15/2018|Australia|03/03/2018	Australia	03/03/2018	3C.2a2		EPI_ISL_314608
A/Sydney/16/2019|Australia|01/18/2019	Australia	01/18/2019	3C.2a1b+131K	Australia	EPI_ISL_346292
A/Sydney/161/2018|Australia|09/04/2018	Australia	09/04/2018	3C.2a1b+135N	Australia	EPI_ISL_332728
A/Sydney/166/2018|Australia|09/15/2018	Australia	09/15/2018	3C.2a1b+135N	Australia	EPI_ISL_332729
A/Sydney/17/2018|Australia|03/05/2018	Australia	03/05/2018	3C.2a2		EPI_ISL_313816
A/Sydney/17/2019|Australia|01/18/2019	Australia	01/18/2019	3C.2a1b+131K	Australia	EPI_ISL_346261
A/Sydney/172/2018|Australia|09/19/2018	Australia	09/19/2018	3C.2a2	Australia	EPI_ISL_332730
A/Sydney/179/2018|Australia|10/07/2018	Australia	10/07/2018	3C.2a2	Australia	EPI_ISL_331776
A/Sydney/18/2018|Australia|03/06/2018	Australia	03/06/2018	3C.2a2		EPI_ISL_314607
A/Sydney/18/2019|Australia|01/18/2019	Australia	01/18/2019	3C.2a1b+131K	Australia	EPI_ISL_346293
A/Sydney/182/2018|Australia|10/18/2018	Australia	10/18/2018	3C.2a2	Australia	EPI_ISL_332734
A/Sydney/19/2018|Australia|02/18/2018	Australia	02/18/2018	3C.2a3		EPI_ISL_314610
A/Sydney/19/2019|Australia|01/19/2019	Australia	01/19/2019	3C.2a1b+131K	Australia	EPI_ISL_346262

A/Sydney/20/2018|Australia|02/16/2018	Australia	02/16/2018	3C.2a1b+135K		EPI_ISL_314609
A/Sydney/20/2019|Australia|01/19/2019	Australia	01/19/2019	3C.2a1b+135K	Australia	EPI_ISL_346294
A/Sydney/21/2019|Australia|01/20/2019	Australia	01/20/2019	3C.2a1b+135K	Australia	EPI_ISL_346295
A/Sydney/22/2018|Australia|03/03/2018	Australia	03/03/2018	3C.2a1b+135N		EPI_ISL_338709
A/Sydney/23/2018|Australia|02/26/2018	Australia	02/26/2018	3C.2a2		EPI_ISL_314606
A/Sydney/24/2018|Australia|02/03/2018	Australia	02/03/2018	3C.2a2		EPI_ISL_314605
A/Sydney/24/2019|Australia|01/20/2019	Australia	01/20/2019	3C.2a1b+135K	Australia	EPI_ISL_346263
A/Sydney/25/2018|Australia|01/27/2018	Australia	01/27/2018	3C.2a2		EPI_ISL_314604
A/Sydney/25/2019|Australia|01/21/2019	Australia	01/21/2019	3C.2a1b+131K	Australia	EPI_ISL_346296
A/Sydney/28/2019|Australia|03/03/2019	Australia	03/03/2019	3C.2a2	Australia	EPI_ISL_354438
A/Sydney/3/2018|Australia|01/26/2018	Australia	01/26/2018	3C.2a2		EPI_ISL_312180
A/Sydney/34/2018|Australia|04/13/2018	Australia	04/13/2018	3C.2a1b+135K		EPI_ISL_321455
A/Sydney/35/2019|Australia|02/14/2019	Australia	02/14/2019	3C.2a1b+131K	Australia	EPI_ISL_353805
A/Sydney/36/2019|Australia|02/15/2019	Australia	02/15/2019	3C.2a1b+135K	Australia	EPI_ISL_354744
A/Sydney/37/2019|Australia|02/18/2019	Australia	02/18/2019	3C.2a1b+135K	Australia	EPI_ISL_354778
A/Sydney/4/2018|Australia|01/30/2018	Australia	01/30/2018	3C.2a2		EPI_ISL_312161
A/Sydney/4/2019|Australia|01/10/2019	Australia	01/10/2019	3C.2a1b+131K	Australia	EPI_ISL_346288
A/Sydney/43/2019|Australia|03/15/2019	Australia	03/15/2019	3C.2a1b+131K	Australia	EPI_ISL_354745
A/Sydney/45/2019|Australia|02/17/2019	Australia	02/17/2019	3C.2a1b+131K	Australia	EPI_ISL_353806
A/Sydney/47/2018|Australia|06/07/2018	Australia	06/07/2018	3C.2a2		EPI_ISL_321589
A/Sydney/5/2018|Australia|01/26/2018	Australia	01/26/2018	3C.2a1b+135K		EPI_ISL_312162
A/Sydney/515/2018|Australia|04/21/2018	Australia	04/21/2018	3C.2a2		EPI_ISL_322339
A/Sydney/52/2019|Australia|03/08/2019	Australia	03/08/2019	3C.2a1b+131K	Australia	EPI_ISL_354717
A/Sydney/521/2018|Australia|04/10/2018	Australia	04/10/2018	3C.2a2		EPI_ISL_329736
A/Sydney/53/2019|Australia|03/08/2019	Australia	03/08/2019	3C.2a1b+131K	Australia	EPI_ISL_356798
A/Sydney/54/2019|Australia|03/11/2019	Australia	03/11/2019	3C.2a1b+131K	Australia	EPI_ISL_354719
A/Sydney/56/2019|Australia|03/12/2019	Australia	03/12/2019	3C.2a1b+131K	Australia	EPI_ISL_354718
A/Sydney/6/2019|Australia|01/10/2019	Australia	01/10/2019	3C.2a1b+131K	Australia	EPI_ISL_346289
A/Sydney/67/2018|Australia|07/02/2018	Australia	07/02/2018	3C.2a2		EPI_ISL_321450
A/Sydney/68/2018|Australia|07/02/2018	Australia	07/02/2018	3C.2a2		EPI_ISL_321451
A/Sydney/7/2018|Australia|01/19/2018	Australia	01/19/2018	3C.2a2		EPI_ISL_312163
A/Sydney/724/2019|Australia|02/11/2019	Australia	02/11/2019	3C.2a1b+135K	Australia	EPI_ISL_354442
A/Sydney/728/2019|Australia|02/15/2019	Australia	02/15/2019	3C.2a1b+135K	Australia	EPI_ISL_354437
A/Sydney/735/2019|Australia|02/22/2019	Australia	02/22/2019	3C.2a1b+131K	Australia	EPI_ISL_354435

A/Sydney/740/2019|Australia|02/26/2019	Australia	02/26/2019	3C.3a	Australia	EPI_ISL_351027
A/Sydney/781/2019|Australia|02/28/2019	Australia	02/28/2019	3C.2a1b+131K	Australia	EPI_ISL_356592
A/Sydney/8/2018|Australia|01/22/2018	Australia	01/22/2018	3C.2a1b+135K		EPI_ISL_312164
A/Sydney/8/2019|Australia|01/10/2019	Australia	01/10/2019	3C.2a1b+131K	Australia	EPI_ISL_346259
A/Sydney/82/2019|Australia|05/05/2019	Australia	05/05/2019	3C.2a1b+131K	Australia	EPI_ISL_362098
A/Sydney/83/2018|Australia|07/04/2018	Australia	07/04/2018	3C.2a2		EPI_ISL_322733
A/Sydney/93/2018|Australia|06/04/2018	Australia	06/04/2018	3C.2a2		EPI_ISL_322734
A/Sydney/98/2018|Australia|04/04/2018	Australia	04/04/2018	3C.2a2		EPI_ISL_331792
A/Taiwan/79440/2018|Asia|07/05/2018	Asia	07/05/2018	3C.2a2		EPI_ISL_330520
A/Taiwan/81988/2019|Asia|03/13/2019	Asia	03/13/2019	3C.2a1b+131K	Global	EPI_ISL_355213
A/Tanger/1449/2018|Africa|03/20/2018	Africa	03/20/2018	3C.2a1b+135K		EPI_ISL_314186
A/Tanzania/2941/2018|Africa|05/06/2018	Africa	05/06/2018	3C.2a3		EPI_ISL_319709
A/Tasmania/1001/2018|Australia|10/02/2018	Australia	10/02/2018	3C.2a2	Australia	EPI_ISL_332745
A/Tasmania/1009/2019|Australia|05/01/2019	Australia	05/01/2019	3C.2a1b+131K	Australia	EPI_ISL_362092
A/Tasmania/501/2019|Australia|01/28/2019	Australia	01/28/2019	3C.2a1b+135K	Australia	EPI_ISL_346264
A/Tasmania/502/2019|Australia|01/05/2019	Australia	01/05/2019	3C.2a1b+131K	Australia	EPI_ISL_346265
A/Tasmania/503/2019|Australia|01/03/2019	Australia	01/03/2019	3C.2a1b+131K	Australia	EPI_ISL_346266
A/Tasmania/506/2019|Australia|03/12/2019	Australia	03/12/2019	3C.2a1b+131K	Australia	EPI_ISL_356598
A/Tasmania/507/2019|Australia|02/04/2019	Australia	02/04/2019	3C.2a1b+131K	Australia	EPI_ISL_356597
A/Tasmania/511/2019|Australia|03/15/2019	Australia	03/15/2019	3C.2a1b+131K	Australia	
A/Tasmania/516/2019|Australia|02/15/2019	Australia	02/15/2019	3C.2a1b+131K	Australia	
A/Tauranga/7/2018|Oceania|07/11/2018	Oceania	07/11/2018	3C.2a2		EPI_ISL_329737
A/Temuco/79791/2018|SouthAmerica|09/03/2018	SouthAmerica	09/03/2018	3C.2a2	Global	EPI_ISL_347103
A/Temuco/86102/2018|SouthAmerica|08/17/2018	SouthAmerica	08/17/2018	3C.2a2		EPI_ISL_347105
A/Temuco/88991/2018|SouthAmerica|08/11/2018	SouthAmerica	08/11/2018	3C.2a2		EPI_ISL_355841
A/Temuco/91589/2018|SouthAmerica|09/09/2018	SouthAmerica	09/09/2018	3C.2a2	Global	EPI_ISL_347091
A/Tennessee/42/2018|NorthAmerica|01/10/2018	NorthAmerica	01/10/2018	3C.2a2		EPI_ISL_360850
A/Tennessee/48/2018|NorthAmerica|02/12/2018	NorthAmerica	02/12/2018	3C.3a		EPI_ISL_360861
A/Tennessee/55/2018|NorthAmerica|11/02/2018	NorthAmerica	11/02/2018	3C.3a	Global	EPI_ISL_333468
A/Texas/07/2018|NorthAmerica|01/08/2018	NorthAmerica	01/08/2018	3C.2a2		EPI_ISL_305084
A/Texas/132/2018|NorthAmerica|09/17/2018	NorthAmerica	09/17/2018	3C.2a2	Global	EPI_ISL_331136
A/Texas/133/2018|NorthAmerica|09/22/2018	NorthAmerica	09/22/2018	3C.2a1b+135K	Global	EPI_ISL_331121
A/Timor-Leste/24/2018|Oceania|03/09/2018	Oceania	03/09/2018	3C.2a1b+135K		EPI_ISL_339060
A/Townsville/04/2018|Australia|03/12/2018	Australia	03/12/2018	3C.2a2		EPI_ISL_313811

A/Townsville/10/2018|Australia|05/31/2018	Australia	05/31/2018	3C.2a2		EPI_ISL_320402
A/Townsville/1000/2018|Australia|02/16/2018	Australia	02/16/2018	3C.2a2		EPI_ISL_314622
A/Townsville/1002/2018|Australia|08/22/2018	Australia	08/22/2018	3C.2a2		EPI_ISL_330730
A/Townsville/1005/2018|Australia|12/12/2018	Australia	12/12/2018	3C.2a1b+131K	Australia	EPI_ISL_339268
A/Townsville/13/2019|Australia|02/25/2019	Australia	02/25/2019	3C.3a	Australia	EPI_ISL_354433
A/Townsville/15/2019|Australia|02/11/2019	Australia	02/11/2019	3C.2a1b+131K	Australia	EPI_ISL_356685
A/Townsville/16/2019|Australia|02/13/2019	Australia	02/13/2019	3C.2a1b+131K	Australia	EPI_ISL_354429
A/Townsville/23/2018|Australia|07/21/2018	Australia	07/21/2018	3C.2a1b+135K		EPI_ISL_322727
A/Townsville/24/2018|Australia|08/07/2018	Australia	08/07/2018	3C.2a2		EPI_ISL_339059
A/Townsville/39/2018|Australia|10/10/2018	Australia	10/10/2018	3C.2a1b+131K	Australia	EPI_ISL_332638
A/Townsville/4/2019|Australia|01/22/2019	Australia	01/22/2019	3C.2a1b+131K	Australia	EPI_ISL_354432
A/Townsville/40/2018|Australia|11/16/2018	Australia	11/16/2018	3C.2a1b+131K	Australia	EPI_ISL_339242
A/Townsville/43/2018|Australia|11/28/2018	Australia	11/28/2018	3C.2a1b+131K	Australia	EPI_ISL_339271
A/Townsville/44/2018|Australia|11/21/2018	Australia	11/21/2018	3C.2a1b+131K	Australia	EPI_ISL_339272
A/Townsville/46/2018|Australia|12/28/2018	Australia	12/28/2018	3C.2a1b+131K	Australia	EPI_ISL_339274
A/Tyumen/RII-01/2018|Europe|06/11/2018	Europe	06/11/2018	3C.2a2		EPI_ISL_330675
A/Tyumen/RII-2/2018|Europe|06/11/2018	Europe	06/11/2018	3C.2a2		EPI_ISL_330033
A/Tyumen/RII-4678S/2018|Europe|06/11/2018	Europe	06/11/2018	3C.2a2		EPI_ISL_322983
A/Tyumen/RII-4680S/2018|Europe|06/11/2018	Europe	06/11/2018	3C.2a2		EPI_ISL_322443
A/Ulaanbaatar/163/2019|Asia|01/09/2019	Asia	01/09/2019	3C.2a1b+131K	Global	EPI_ISL_341450
A/Usk/3844/2019|Europe|05/07/2019	Europe	05/07/2019	3C.2a1b+131K	Global	EPI_ISL_357339
A/Usk/3849/2019|Europe|05/07/2019	Europe	05/07/2019	3C.2a1b+131K	Global	EPI_ISL_357370
A/Usk/3851/2019|Europe|05/07/2019	Europe	05/07/2019	3C.2a1b+131K	Global	EPI_ISL_357372
A/Valdiva/100553/2018|SouthAmerica|12/11/2018	SouthAmerica	12/11/2018	3C.2a2	Global	EPI_ISL_351875
A/Valladolid/17/2019|Europe|01/12/2019	Europe	01/12/2019	3C.3a	Global	EPI_ISL_355122
A/Valparaiso/54264/2018|SouthAmerica|06/28/2018	SouthAmerica	06/28/2018	3C.2a2		EPI_ISL_330281
A/Venezuela/29/2018|SouthAmerica|11/23/2018	SouthAmerica	11/23/2018	3C.2a1b+135K	Global	EPI_ISL_348139
A/Vermont/06/2019|NorthAmerica|02/06/2019	NorthAmerica	02/06/2019	3C.2a1b+135K	Global	EPI_ISL_353696
A/Vermont/09/2019|NorthAmerica|02/11/2019	NorthAmerica	02/11/2019	3C.2a1b+135K	Global	EPI_ISL_353636
A/Vermont/22/2018|NorthAmerica|04/25/2018	NorthAmerica	04/25/2018	3C.2a2		EPI_ISL_312821
A/Victoria/1/2019|Australia|01/04/2019	Australia	01/04/2019	3C.2a1b+131K	Australia	EPI_ISL_339260
A/Victoria/1003/2018|Australia|10/04/2018	Australia	10/04/2018	3C.2a2	Australia	EPI_ISL_332645
A/Victoria/1004/2018|Australia|10/03/2018	Australia	10/03/2018	3C.2a1b+135K	Australia	EPI_ISL_332744
A/Victoria/17/2018|Australia|09/14/2018	Australia	09/14/2018	3C.2a1b+135K	Australia	EPI_ISL_330116

A/Victoria/2/2019|Australia|01/04/2019	Australia	01/04/2019	3C.2a1b+131K	Australia	EPI_ISL_340091
A/Victoria/20/2019|Australia|04/11/2019	Australia	04/11/2019	3C.2a1b+131K	Australia	EPI_ISL_356601
A/Victoria/2028/2019|Australia|01/16/2019	Australia	01/16/2019	3C.2a1b+131K	Australia	EPI_ISL_354499
A/Victoria/2036/2018|Australia|08/17/2018	Australia	08/17/2018	3C.2a2		EPI_ISL_323107
A/Victoria/2038/2018|Australia|08/22/2018	Australia	08/22/2018	3C.2a1b+135K		EPI_ISL_330734
A/Victoria/2042/2018|Australia|08/28/2018	Australia	08/28/2018	3C.2a1b+135N		EPI_ISL_331775
A/Victoria/2051/2018|Australia|09/08/2018	Australia	09/08/2018	3C.2a1b+131K	Australia	EPI_ISL_331781
A/Victoria/2059/2018|Australia|09/16/2018	Australia	09/16/2018	3C.2a2	Australia	EPI_ISL_331782
A/Victoria/2067/2019|Australia|02/05/2019	Australia	02/05/2019	3C.2a1b+131K	Australia	EPI_ISL_354424
A/Victoria/2072/2019|Australia|02/09/2019	Australia	02/09/2019	3C.2a1b+131K	Australia	EPI_ISL_354425
A/Victoria/2073/2019|Australia|02/10/2019	Australia	02/10/2019	3C.2a1b+131K	Australia	EPI_ISL_354436
A/Victoria/2077/2019|Australia|02/12/2019	Australia	02/12/2019	3C.2a1b+131K	Australia	EPI_ISL_354426
A/Victoria/2080/2019|Australia|02/13/2019	Australia	02/13/2019	3C.2a3	Australia	EPI_ISL_354427
A/Victoria/2086/2019|Australia|02/18/2019	Australia	02/18/2019	3C.3a	Australia	EPI_ISL_354423
A/Victoria/2087/2019|Australia|02/20/2019	Australia	02/20/2019	3C.2a1b+131K	Australia	EPI_ISL_354428
A/Victoria/2088/2018|Australia|10/02/2018	Australia	10/02/2018	3C.2a2	Australia	EPI_ISL_341295
A/Victoria/2091/2019|Australia|02/24/2019	Australia	02/24/2019	3C.2a1b+131K	Australia	EPI_ISL_354733
A/Victoria/2093/2018|Australia|10/03/2018	Australia	10/03/2018	3C.2a1b+135K	Australia	EPI_ISL_334091
A/Victoria/2094/2019|Australia|02/24/2019	Australia	02/24/2019	3C.2a1b+131K	Australia	EPI_ISL_354746
A/Victoria/2095/2019|Australia|02/26/2019	Australia	02/26/2019	3C.2a1b+135K	Australia	EPI_ISL_354734
A/Victoria/2100/2019|Australia|03/15/2019	Australia	03/15/2019	3C.2a1b+131K	Australia	
A/Victoria/2103/2019|Australia|03/15/2019	Australia	03/15/2019	3C.2a1b+131K	Australia	
A/Victoria/2104/2018|Australia|10/08/2018	Australia	10/08/2018	3C.2a1b+135K	Australia	EPI_ISL_334089
A/Victoria/2108/2019|Australia|03/15/2019	Australia	03/15/2019	3C.2a1b+131K	Australia	
A/Victoria/2118/2018|Australia|10/22/2018	Australia	10/22/2018	3C.2a3	Australia	EPI_ISL_339074
A/Victoria/2120/2018|Australia|10/24/2018	Australia	10/24/2018	3C.2a1b+131K	Australia	EPI_ISL_338269
A/Victoria/2123/2018|Australia|10/28/2018	Australia	10/28/2018	3C.2a1b+131K	Australia	EPI_ISL_339057
A/Victoria/2139/2018|Australia|11/11/2018	Australia	11/11/2018	3C.2a1b+131K	Australia	EPI_ISL_339228
A/Victoria/2143/2018|Australia|11/13/2018	Australia	11/13/2018	3C.2a1b+131K	Australia	EPI_ISL_339264
A/Victoria/2147/2018|Australia|11/19/2018	Australia	11/19/2018	3C.2a1b+135K	Australia	EPI_ISL_339229
A/Victoria/2149/2018|Australia|11/20/2018	Australia	11/20/2018	3C.2a1b+131K	Australia	EPI_ISL_339265
A/Victoria/2152/2018|Australia|11/21/2018	Australia	11/21/2018	3C.2a1b+135K	Australia	EPI_ISL_339230
A/Victoria/2153/2019|Australia|03/15/2019	Australia	03/15/2019	3C.2a1b+131K	Australia	
A/Victoria/2158/2019|Australia|03/15/2019	Australia	03/15/2019	3C.2a1b+131K	Australia	

A/Victoria/2161/2019|Australia|03/15/2019	Australia	03/15/2019	3C.2a1b+131K	Australia	
A/Victoria/2166/2018|Australia|12/02/2018	Australia	12/02/2018	3C.2a1b+135K	Australia	EPI_ISL_339235
A/Victoria/23/2018|Australia|10/01/2018	Australia	10/01/2018	3C.2a1b+131K	Australia	EPI_ISL_331784
A/Victoria/250/2019|Australia|04/01/2019	Australia	04/01/2019	3C.2a1b+131K	Australia	EPI_ISL_356615
A/Victoria/250/2019|Australia|04/11/2019	Australia	04/11/2019	3C.2a1b+131K	Australia	EPI_ISL_356615
A/Victoria/2500/2018|Australia|12/31/2018	Australia	12/31/2018	3C.2a1b+131K	Australia	EPI_ISL_339052
A/Victoria/2506/2018|Australia|12/31/2018	Australia	12/31/2018	3C.2a1b+131K	Australia	EPI_ISL_339053
A/Victoria/28/2018|Australia|10/02/2018	Australia	10/02/2018	3C.2a1b+131K	Australia	EPI_ISL_331785
A/Victoria/40/2018|Australia|11/07/2018	Australia	11/07/2018	3C.2a1b+131K	Australia	EPI_ISL_340096
A/Victoria/703/2019|Australia|04/07/2019	Australia	04/07/2019	3C.3a	Australia	EPI_ISL_356797
A/Victoria/708/2018|Australia|09/08/2018	Australia	09/08/2018	3C.2a1b+135K	Australia	EPI_ISL_331779
A/Victoria/708/2019|Australia|04/07/2019	Australia	04/07/2019	3C.2a1b+131K	Australia	EPI_ISL_356627
A/Victoria/718/2018|Australia|08/29/2018	Australia	08/29/2018	3C.2a1b+135K		EPI_ISL_331778
A/Victoria/731/2018|Australia|09/10/2018	Australia	09/10/2018	3C.2a2	Australia	EPI_ISL_331780
A/Victoria/743/2018|Australia|11/25/2018	Australia	11/25/2018	3C.2a1b+131K	Australia	EPI_ISL_338271
A/Victoria/747/2018|Australia|11/13/2018	Australia	11/13/2018	3C.2a2	Australia	EPI_ISL_338272
A/Victoria/753/2018|Australia|11/07/2018	Australia	11/07/2018	3C.2a1b+131K	Australia	EPI_ISL_336142
A/Victoria/8/2019|Australia|01/29/2019	Australia	01/29/2019	3C.2a1b+135N	Australia	EPI_ISL_346299
A/Victoria/9/2019|Australia|01/30/2019	Australia	01/30/2019	3C.2a1b+135N	Australia	EPI_ISL_342171
A/Victoria/901/2018|Australia|08/23/2018	Australia	08/23/2018	3C.2a2		EPI_ISL_330732
A/Victoria/908/2018|Australia|09/27/2018	Australia	09/27/2018	3C.2a2	Australia	EPI_ISL_332675
A/Victoria/909/2018|Australia|10/27/2018	Australia	10/27/2018	3C.2a2	Australia	EPI_ISL_332743
A/Victoria/909/2019|Australia|02/28/2019	Australia	02/28/2019	3C.2a1b+131K	Australia	EPI_ISL_351028
A/Victoria/912/2018|Australia|12/07/2018	Australia	12/07/2018	3C.2a3	Australia	EPI_ISL_338279
A/Victoria/913/2019|Australia|03/18/2019	Australia	03/18/2019	3C.2a1b+131K	Australia	EPI_ISL_354732
A/Victoria/925/2019|Australia|03/27/2019	Australia	03/27/2019	3C.2a1b+131K	Australia	EPI_ISL_356636
A/Victoria/927/2019|Australia|03/29/2019	Australia	03/29/2019	3C.2a1b+131K	Australia	EPI_ISL_356637
A/Victoria/928/2019|Australia|03/23/2019	Australia	03/23/2019	3C.2a1b+131K	Australia	EPI_ISL_356638
A/Victoria/934/2019|Australia|03/29/2019	Australia	03/29/2019	3C.3a	Australia	EPI_ISL_356630
A/Vietnam/205/2018|Asia|09/07/2018	Asia	09/07/2018	3C.2a1b+131K	Global	EPI_ISL_336736
A/Vietnam/214/2018|Asia|08/01/2018	Asia	08/01/2018	3C.2a1b+131K		EPI_ISL_335609
A/Virginia/03/2019|NorthAmerica|01/08/2019	NorthAmerica	01/08/2019	3C.3a	Global	EPI_ISL_348131
A/Wallis_Futuna/2/2019|Australia|03/15/2019	Australia	03/15/2019	3C.2a1b+131K	Australia	
A/Washington/186/2018|NorthAmerica|10/26/2018	NorthAmerica	10/26/2018	3C.2a2	Global	EPI_ISL_336759

A/Wellington/10/2019|Oceania|03/23/2019	Oceania	03/23/2019	3C.2a1b+131K	Global	EPI_ISL_356626
A/Wellington/11/2019|Oceania|03/28/2019	Oceania	03/28/2019	3C.2a1b+131K	Global	EPI_ISL_356620
A/Wellington/15/2019|Oceania|03/21/2019	Oceania	03/21/2019	3C.2a1b+131K	Global	EPI_ISL_356621
A/Wisconsin/385/2019|NorthAmerica|03/24/2019	NorthAmerica	03/24/2019	3C.3a	Global	EPI_ISL_362422
A/Wisconsin/404/2019|NorthAmerica|05/01/2019	NorthAmerica	05/01/2019	3C.3a	Global	EPI_ISL_357875
A/Wisconsin/452/2018|NorthAmerica|03/02/2018	NorthAmerica	03/02/2018	3C.2a2		EPI_ISL_361524
A/Wisconsin/96/2018|NorthAmerica|08/04/2018	NorthAmerica	08/04/2018	3C.2a1b+135N		EPI_ISL_330496
A/Wisconsin/99/2018|NorthAmerica|08/17/2018	NorthAmerica	08/17/2018	3C.2a1b+135N		EPI_ISL_330883
A/YAMAGATA/189/2018|Asia|05/21/2018	Asia	05/21/2018	3C.2a2		EPI_ISL_320564
A/Yunnan-Wuhua/19/2019|Asia|01/02/2019	Asia	01/02/2019	3C.2a1b+135K	Global	EPI_ISL_337308
A/Zambia/4-352/2018|Africa|09/03/2018	Africa	09/03/2018	3C.2a2	Global	EPI_ISL_361964
A/Zaragoza/1512154/2018|Europe|06/09/2018	Europe	06/09/2018	3C.2a2		EPI_ISL_330681
A/Zaragoza/1528894/2018|Europe|07/09/2018	Europe	07/09/2018	3C.2a2		EPI_ISL_330683
A/Zaragoza/489/2018|Europe|07/16/2018	Europe	07/16/2018	3C.2a2		EPI_ISL_330686
A/Zaragoza/490/2018|Europe|05/17/2018	Europe	05/17/2018	3C.2a2		EPI_ISL_330690
A/Zaragoza/492/2018|Europe|07/12/2018	Europe	07/12/2018	3C.2a2		EPI_ISL_330698
A/Zaragoza/493/2018|Europe|08/23/2018	Europe	08/23/2018	3C.2a2		EPI_ISL_330700

Supplementary Table 2 Final Fold X values

This supplementary material is hosted by *Eurosurveillance* as supporting information alongside the article "Intense interseasonal influenza outbreaks in Australia in 2018-9" on behalf of the authors who remain responsible for the accuracy and appropriateness of the content. The same standards for ethics, copyright, attributions and permissions as for the article apply. *Eurosurveillance* is not responsible for the maintenance of any links or email addresses provided therein

Subtype	Subtitution	Vaccine-WT reference	Mean ddG (kcal/mol)	S.D.
H3	E62G	A1a/A2re	-1.46	0.02
H3	K92R	A1a/A2re	-0.58	0.26
H3	T131K	A1a	-0.57	0.10
H3	Q197R	A1a/A2re	-0.45	0.25
H3	K207R	A1a	0.06	0.07
H3	S219F	A1a/A2re	-0.99	0.02
H3	H311Q	A1a/A2re	0.43	0.22
H3	V347M	A1a/A2re	-0.05	0.00
H3	E479G	A1a	1.39	0.03
H3	E484G	A1a	-2.65	0.11
H3	V529I	A1a/A2re	Position not observed in structure.	
H3	N121K	A2re	-0.35	0.14
H3	N171K	A2re	-0.56	0.04
H3	S96N	A2re	0.12	0.13
H3	I406V	A2re	0.68	0.00
H3	K142G	A2re	-0.01	0.01
H3	Q261R	A2re	-0.76	0.16
H1pdm09	S74R	A/Michigan/45/2015	1.10	0.08
H1pdm09	N129D	A/Michigan/45/2015	0.32	0.01
H1pdm09	S164T	A/Michigan/45/2015	0.71	0.14
H1pdm09	S183P	A/Michigan/45/2015	-2.96	0.09

H1pdm09	T185I	A/Michigan/45/2015	-0.89	0.00
H1pdm09	R223Q	A/Michigan/45/2015	-1.00	0.33
H1pdm09	N260D	A/Michigan/45/2015	-0.93	0.09
H1pdm09	I295V	A/Michigan/45/2015	0.74	0.03
H1pdm09	K504R	A/Michigan/45/2015	Position not observed in structure.	

*H3 vaccine reference: A1a = A/Singapore/Infimh-16-0019/2016; A2re = A/Switzerland/8060/2017

*PBD = 4O5N (H3); 3UBQ (H1pdm09)

*Stabilising ddG values highlighted in green/destabilising values in red.
