# Supplementary material for: Intense interseasonal influenza outbreaks, Australia, 2018/19
Source: Euro Surveill. 2019 Aug 15;24(33):1900421. doi: 10.2807/1560-7917.ES.2019.24.33.1900421 (PMC6702793; doi:10.2807/1560-7917.ES.2019.24.33.1900421)
Supplement: Supplementary Figure [file 19-00421_BARR_SupplementaryFigure5c.pdf]

This supplementary material is hosted by Eurosurveillance as supporting information alongside the article “Intense interseasonal influenza outbreaks in Australia in 2019-9” on behalf of the authors who remain responsible for the accuracy and appropriateness of the content. The same standards for ethics, copyright, attributions and permissions as for the article apply. Eurosurveillance is not responsible for the maintenance of any links or email addresses provided therein.

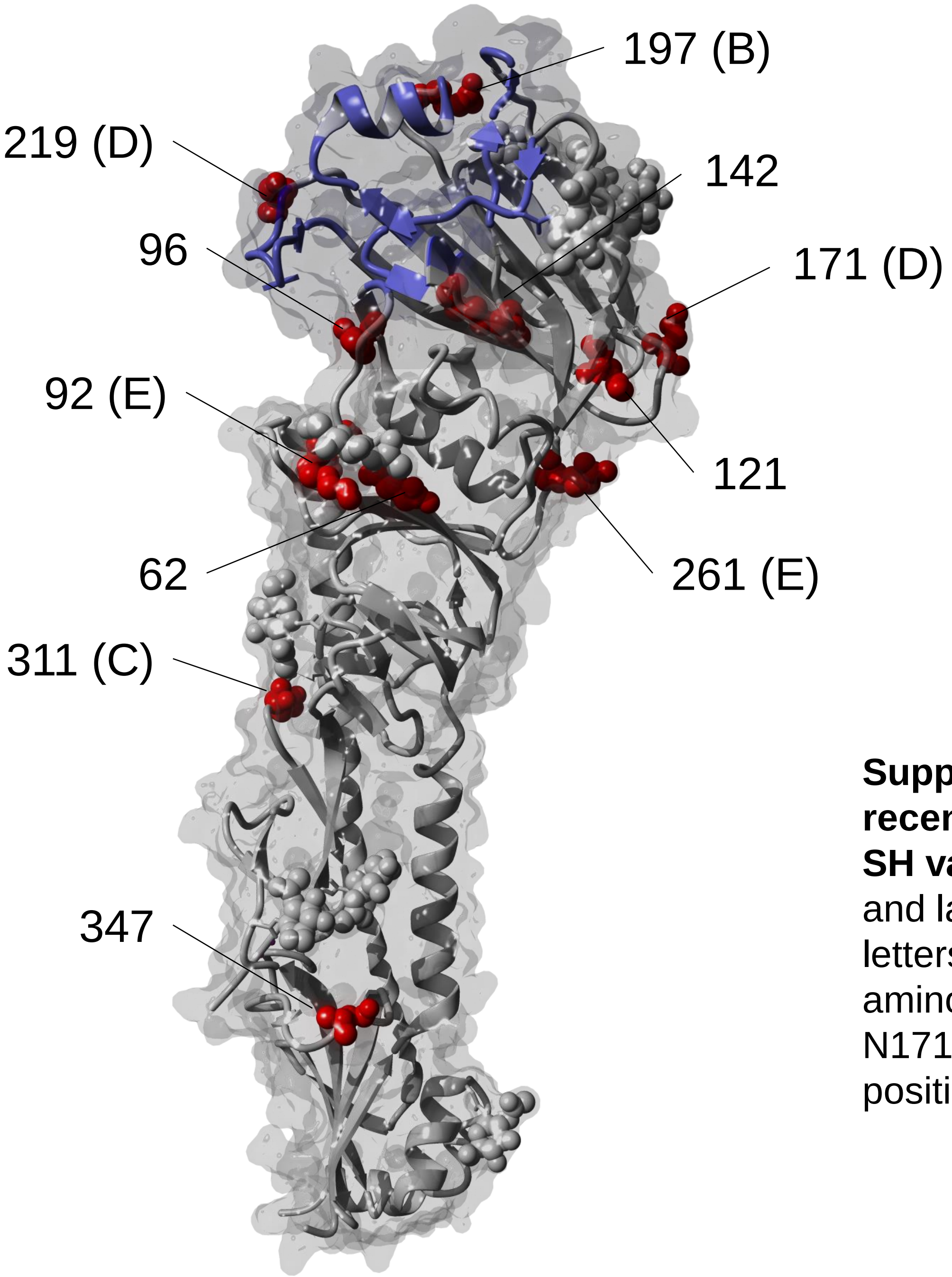

Supplementary Figure 5C: Amino acid differences in recent in HA of the recent 3C2a1b+131K H3 clade viruses relative to the 2019 Australian and SH vaccine A/Switzerland/8060/2017 (H3N2). Changes are shown in red and labelled according to H3 position numbering without signal peptide, letters in brackets indicate if this change was in a known antigenic site. The amino acid substitutions are as follows: E62G, K92R, S96N, N121K, K142G, N171K, Q197R, S219F, Q261R, H311Q, V347M, I406V, and V529I. Note that position 406 is not observed in the HA structure (PDB: 4WEA).
